# The SET29 and SET7 proteins of *Leishmania donovani* exercise non-redundant convergent as well as collaborative functions in moderating the parasite’s response to oxidative stress

**DOI:** 10.1016/j.jbc.2025.108208

**Published:** 2025-01-20

**Authors:** Varshni Sharma, Jyoti Pal, Vishal Dashora, Somdeb Chattopadhyay, Yogita Kapoor, Biplab Singha, G. Aneeshkumar Arimbasseri, Swati Saha

**Affiliations:** 1 Department of Microbiology, University of Delhi South Campus, New Delhi, India; 2 National Institute of Immunology, New Delhi, India; 3 Centre for Cellular and Molecular Biology, Hyderabad, India; 4 Academy of Scientific and Innovative Research (AcSIR), Ghaziabad, India

**Keywords:** *Leishmania donovani*, trypanosome, SET domain, SET proteins, SET29, SET7, oxidative stress, protozoan parasite, BiFC in *leishmania*

## Abstract

SET proteins are lysine methyltransferases. In investigating *Leishmania donovani* SET29, we found depletion of LdSET29 by two-thirds did not affect promastigote growth, nor alter the parasite’s response to UV-induced or HU-induced stress, but made it more tolerant to H_2_O_2_-induced oxidizing environment. The deviant response to oxidative stress was coupled to lowered accumulation of reactive oxygen species, which was linked to enhanced scavenging activity. The *set29* mutants’ response to H_2_O_2_ exposure was similar to that of *set7* mutants, prompting an investigation into genetic and physical interactions between the two proteins. While neither protein could rescue the aberrant phenotypes of the other *set* mutant, the two proteins interacted physically *in vitro* and *in vivo*. Transcriptome analyses revealed that neither protein regulated global gene expression, but LdSET7 controlled transcript levels of a limited number of genes, including several peroxidases. In working towards identifying targets through which SET7/SET29 mediate the cell’s response to an oxidative milieu, we found HSP60/CNP60 and TCP1 to be possible candidates. LdHSP60 has earlier been implicated in the regulation of the response of virulent promastigotes to H_2_O_2_ exposure, and LdTCP1 has previously been found to have a protective effect against oxidative stress. *set7* and *set29* mutants survived more proficiently in host macrophages as well. The data suggest an alliance between LdSET29 and LdSET7 in mounting the parasite’s response to oxidative stress, each protein playing its own distinctive role. They ensure the parasite not only establishes infection but also maintains the balance with host cells to enable the persistence of infection.

The group of diseases called Leishmaniases primarily burden the world’s poor, whose immune systems are too weak to combat infection, and who do not have access to sanitary living conditions. Endemic in 90 countries, leishmaniasis is manifested as cutaneous leishmaniasis (CL), subcutaneous leishmaniasis, and visceral leishmaniasis (VL). More than a billion people live in the areas to which Leishmaniases are endemic, thus putting them at risk of infection; however, most people who get infected do not develop the disease. Every year ∼30,000 new VL cases and >1 million new CL cases are reported (https://www.who.int/health-topics/leishmaniasis). The majority of VL cases occur in Eastern Africa, South America, and the Indian subcontinent. In the battle against VL (caused by *Leishmania donovani* in India), fresh threats have emerged from rising cases of drug resistance, post kala-azar dermal leishmaniasis (PKDL), and *Leishmania*-HIV co-infections. The pathogen is a digenetic protozoan, shuttling between the sandfly and mammalian hosts, and in the absence of a vaccine, the control of the insect host population remains the most effective method for prevention.

*Leishmania* species (like other trypanosomatids) display an unusual mode of genome organization and gene transcription, with functionally unrelated genes lying in long unidirectional clusters that are typically transcribed polycistronically and constitutively (1). Gene expression in trypanosomatids is regulated by epigenetic mechanisms, post-transcriptional processes including regulation of mRNA stability by RNA-binding proteins (RBPs), and translational control (2, 3, 4), and in case of *Leishmania* species that have plastic genomes and exhibit mosaic aneuploidy, gene dosage plays an important role as well (5, 6). No consensus sequences have been identifiable at Transcriptional Start Regions (TSRs) in trypanosomatids, but TSRs are enriched in GT-rich elements and three epigenetic marks: H2A.Z hyperacetylation, H4K10 acetylation, and H3K4 methylation, and while the roles of the acetylation events and the enzymes that mediate them have been uncovered, the role of the H3K4 methylation event in regulating transcription and the enzyme which mediates it remains unknown (7, 8, 9, 10). Histone lysine residues are typically methylated by SET domain proteins. The effects of SET proteins go beyond epigenetic regulation through histone modifications, as they target non-histone substrates as well (reviewed in (11, 12)). Through the methylation of transcriptional activators/repressors, they upregulate as well as downregulate gene expression (13). They have been found to methylate RNA-binding proteins as well (14), and with the identification of a large number of lysine methylations in RNA binding proteins in human cells, they are believed to play an important role in controlling transcript stability (15). SET protein-mediated lysine methylations also play a role in regulating protein stability and activity (16, 17).

The family of trypanosomatids, to which *Leishmania* species belong, includes several closely related unicellular parasitic pathogens, the best studied among them being the African trypanosome *Trypanosoma brucei* which causes sleeping sickness. Trypanosomatid SET domain proteins have been examined to a limited extent only in *T. brucei*, and although 29 SET domain proteins have been annotated in the *T. brucei* genome and their subcellular localizations determined (18, 19, 20), their target substrates have not been identified. The only TbSET protein characterized to date is the nuclear TbSET27, found to be a part of a multi-protein complex (SPARC) that localizes to Transcription Start Regions (TSRs) and playing a role in regulating the accuracy of transcription start events (21). Orthologs of all 29 TbSET proteins are identifiable in *Leishmania* species, and the *Leishmania* and *T. brucei* orthologs share a high percent of sequence identity. The functional role of the *L. donovani* SET protein LdSET7 has been reported (22). LdSET7 moderates the parasite’s response to an oxidative environment in *in vitro* cultures and in mammalian host cells. The L. *tarentolae* SET protein LtaP35.2400 has been identified to be a part of a multiprotein complex, whose other component JBP3 modulates transcription termination events (23).

The present study investigates the role of the *L. donovani* SET protein, LdSET29. Our results suggest that the *set29* gene is essential for parasite cell survival. Interestingly, partial depletion of LdSET29 has a seemingly opposite effect, with depletion of LdSET29 up to ∼ one-third of its normal expression levels not having any significant impact on *in vitro* growth of the parasites under normal conditions. However, depletion of LdSET29 to even half its normal expression levels enables the parasite to tolerate much higher levels of oxidative stress. Further investigations suggest that LdSET29 may work alongside LdSET7 in moderating the parasite’s response to an oxidative environment, through the concerted targeting of certain substrates.

## Results

### *L. donovani* SET29 protein is ubiquitous in the promastigote cell

In initiating studies on *L.donovani* SET proteins we selected six proteins based on their subcellular localization, picking a mix of nuclear and cytosolic proteins. In this study working towards determining the functional role of LdSET29, we began with cloning the *set29* gene and analyzing the expression of the protein in *Leishmania* parasites. In the absence of *L. donovani* 1S genome sequence information, the Ld1S *set29* gene was amplified off its genomic DNA using primers designed against the LdBPK_212120.1 gene ((24); https://tritrypdb.org; Methods), and the ∼1.16 kb amplicon obtained was cloned and sequenced (GenBank Accession no: OR479703). Analysis of its derived amino acid sequence using Clustal Omega and Blastp ((25); https://blast.ncbi.nlm.nih.gov.in) revealed the protein to share 48% to 50% identity over 98% coverage with *Trypanosoma* SET29 proteins, and 90% to 100% identity over 100% coverage with SET29 protein of other *Leishmania* species (Fig. S1*A*). Two conserved domains were identified in LdSET29: a PHD (plant homeo domain) finger in the N-terminal part of the protein which may play a role in facilitating interactions with its substrate protein, and a SET domain in the C-terminal half of the protein which typically catalyzes the methyltransferase reaction (Fig. S1*B*). The SET domain harbored all the motifs characteristically found in this domain.

To analyze the expression of LdSET29 in *Leishmania* cells, we used antibodies raised against the recombinant protein to probe *Leishmania* whole cell extracts. For this, the *set29* gene was expressed in *E. coli*, the recombinant LdSET29 protein was purified to near homogeneity (Experimental procedures; Fig. S2*A*) and mice were immunized (Experimental procedures). The antisera were sensitive enough to detect 1 to 2 ng recombinant protein (Fig. S2*B*), detecting expression of LdSET29 in lysates isolated from 2 × 10^7^ cells (Fig. S2*C*). LdSET29 was equivalently expressed in logarithmically growing and stationary phase promastigotes (Fig. S2*D*). *Leishmania* parasites exist in the sandfly’s midgut as extracellular flagellate non-infective procyclic promastigotes, later developing into infective metacyclic promastigotes that migrate to the insect’s salivary glands. The metacyclics get released into the mammalian host’s bloodstream with the insect bite, whereupon they are phagocytosed by macrophages within which they take up residence. LdSET29 was expressed to similar extents in procyclics and metacyclics (Fig. S2*E*).

As the antibodies did not stain cells in immunofluorescence experiments, the subcellular localization of LdSET29 was analyzed by expressing it in *Leishmania* promastigotes in fusion with an HA tag at its C-terminus (Experimental procedures; Fig. S3*A*), and examining the cells using immunofluorescence microscopy with the help of anti-HA antibodies. Using kinetoplast morphology and segregation pattern as cell cycle stage markers in individual cells (26), the LdSET29-HA protein was found to be evenly distributed across the cytosol as well as the nucleus at all stages of the cell cycle (Fig. S3*B*), similar to the distribution pattern of the TbSET29 protein which was found to be cytosolic as well as nuclear (19), but unlike LdSET7 which is predominantly cytosolic with nuclear localization being only weakly detected (22). Thus, we concluded that LdSET29 was expressed in all stages of the promastigote parasite, and was found throughout the cell.

### Partial depletion of LdSET29 makes the parasites more tolerant to an oxidative milieu

**Figure 1:**
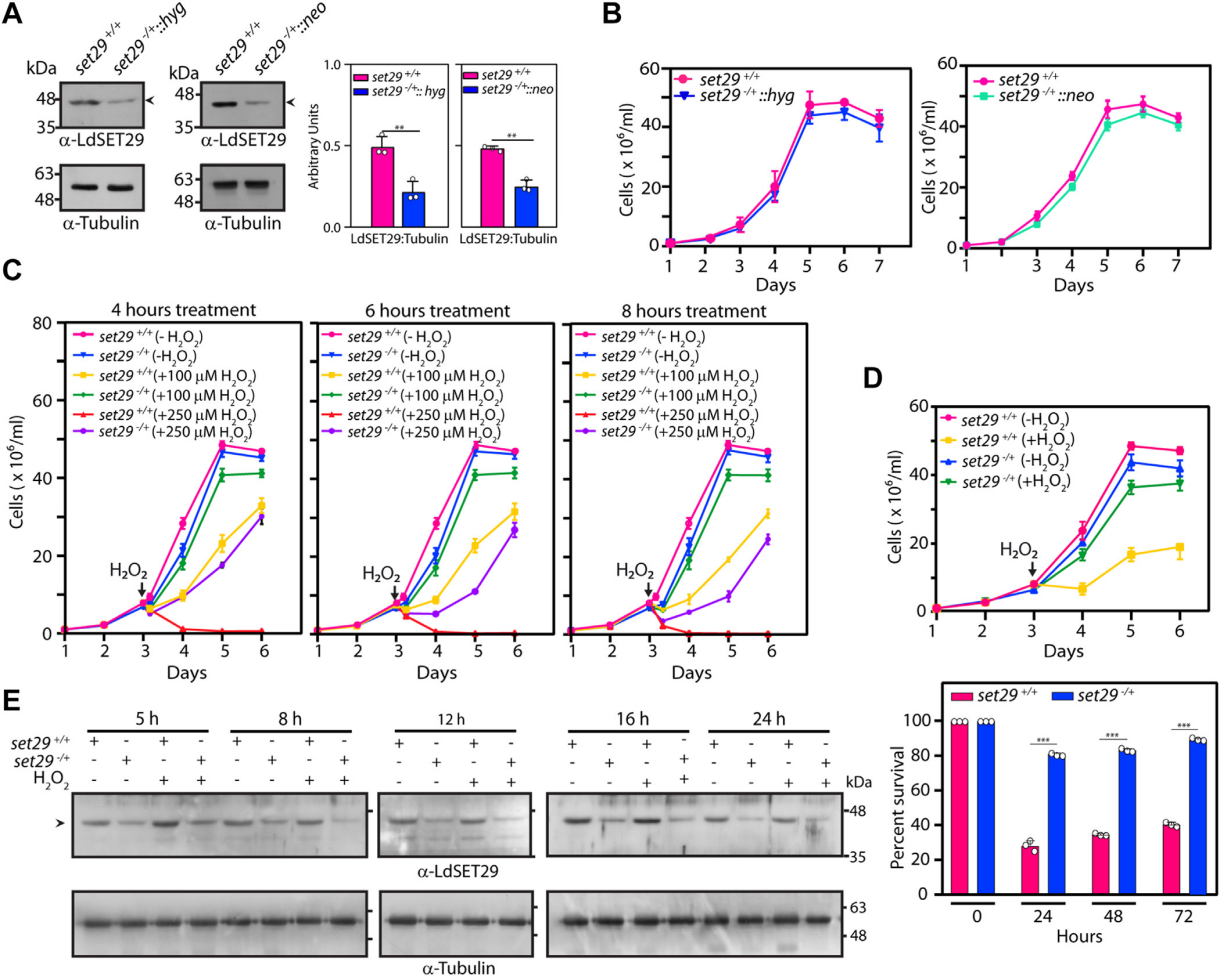
**Analysis of *set29***^***−/+***^**promastigotes.***A*, Western blot analysis of *Leishmania* promastigote lysates isolated from 1 × 10^8^ logarithmically growing cells, using anti-SET29 antibodies (1:1000 dilution). Tubulin served as loading control. ImageJ analysis (https://imagej.net/ij/) was used for quantitation. Bar graphs represent mean values of three experiments, with error bars depicting standard deviation. *Open circles* on bar graphs mark values of individual experiments. Statistical significance was evaluated using two-tailed unpaired student’s *t* test. ∗∗ represents *p* value <0.005. *B*, analysis of growth of parasites. *Left panel: set29*^*−/+*^*::hyg* heterozygous knockout cells. *Right panel*: *set29*^*−/+*^*::neo* heterozygous knockout cells. Each of the two growth analysis experiments was performed thrice, with two technical replicates in each biological. Values shown are mean values of the three experiments, with error bars depicting standard deviation. *C*, effect of H_2_O_2_ exposure on growth of *set29*^*−/+*^ promastigotes. Cultures (*set29*^*+/+*^ and *set29*^*−/+*^ parasites) were initiated from stationary phase promastigotes, with 0 to 250 μM H_2_O_2_ being added on Day 3 (48 h after initiation). Each of the two cultures were divided into multiple parts at the time of addition of H_2_O_2_, with one part being continued as the untreated cells and the other parts receiving 100 μM or 250 μM H_2_O_2_. After exposure to H_2_O_2_ for 4 h, 6 h or 8 h, the cultures were replenished with fresh H_2_O_2_ -free M199. Cells were counted every 24 h from the start of H_2_O_2_ exposure. The experiment was performed three times. Values shown are mean values of the three experiments, with error bars depicting standard deviation. The experimental data for each H_2_O_2_ incubation time is shown in a separate panel for easier observation, and hence the (-H_2_O_2_) graph lines are the same in all three panels. *D*, effect of prolonged H_2_O_2_ exposure on growth of *set29*^*−/+*^ promastigotes. Cultures (*set29*^*+/+*^ and *set29*^*−/+*^ parasites) were initiated from stationary phase promastigotes, with 100 μM H_2_O_2_ being added on Day 3 (48 h after initiation). Each of the two cultures were divided into two parts at the time of addition of H_2_O_2_, with one part being continued as the untreated cells and the other part receiving 100 μM H_2_O_2_. H_2_O_2_-treated cultures were maintained by replenishing the H_2_O_2_ every 10 h. *Upper panel*: Growth analysis. *Lower panel*: Percent survival of cells in H_2_O_2_ -treated cultures in comparison with untreated cultures, calculated by dividing the number of cells in treated cultures by the number of cells in untreated cultures and multiplying by 100. The experiment was performed three times, with two technical replicates for each biological. Values shown are mean values of the three experiments, with error bars depicting standard deviation. *Open circles* on bar graphs mark values of individual experiments. Statistical significance was determined using the two-tailed unpaired student’s *t* test. ∗∗∗: *p* value <0.0005. *E*, analysis of whole cell lysates of 8 x 10^7^ promastigotes after exposure to 100 μM H_2_O_2_ for 5 h, using anti-SET29 antibodies (1:1000 dilution). Tubulin served as loading control. The indicated times are with reference to start of H_2_O_2_ exposure. *set29*^*+/+*^ cells: Ld1S::hyg cells. *set29*^*−/*+^ cells: *set29-*heterozygous knockout cells with one genomic allele replaced with the hygromycin resistance cassette.

To examine the physiological relevance of LdSET29 we adopted the strategy of making *set29* genomic knockouts and analyzing the phenotypes obtained since these parasites lack canonical RNAi machinery. The *set29* gene lies on chromosome 21, typically a disomic chromosome in this organism that has a plastic genome, and where some chromosomes have been reported to be trisomic (5). Thus, we worked towards sequentially replacing the two *set29* genomic alleles by homologous recombination (detailed in Experimental procedures). Two heterozygous knockout lines were made, wherein one *set29* allele in Ld1S was replaced with either a hygromycin resistance cassette or a neomycin resistance cassette, with the authenticity of recombination at both ends being checked by PCRs across the deletion junctions (Fig. S4, *A* and *B*). Western blot analyses of whole cell lysates of these two mutant cell types (named *set29*^*−/+*^*::hyg* and *set29*^*−/+*^*::neo* respectively) revealed that LdSET29 expression was reduced to ∼ 50% of the levels detected in wild-type cells (Fig. 1*A*). The growth patterns of the mutant promastigotes resembled that of wild-type promastigotes despite reduced levels of LdSET29 (Fig. 1*B*). This was also reflected in the cell cycle progression pattern of *set29*^*−/+*^ promastigotes, where flow cytometry analyses of cells synchronized at the G1/S boundary with hydroxyurea (26), determined that *set29*^*−/+*^ cells traversed S phase, G2/M, and returned into G1, at a pace comparable with that of *set29*^*+/+*^ cells (Fig. S5*A* shows data acquired using *set29*^*−/+*^*::hyg* cells). Responses to UV irradiation and hydroxyurea-induced chronic stress were similar in *set29*^*−/+*^ and *set29*^*+/*^^*+*^ parasites (Experimental procedures, Fig. S5, *B* and *C*).

To examine the response of *set29*^*−/+*^ promastigotes to an H_2_O_2_-induced oxidizing environment, logarithmically growing *set29*^*−/+*^ promastigotes were exposed to H_2_O_2_ (100 or 250 μM) for variable times (4–8 h) before refeeding them and monitoring their recovery and growth (detailed in Experimental procedures). As evident from Figure 1*C* (left panel), even a 4-h exposure to 100 μM H_2_O_2_ partially compromised *set29*^*+/+*^ cell growth, and *set29*^*+/+*^ cells did not recover from exposure to 250 μM H_2_O_2_. Contrastingly, *set29*^*−/+*^ cells were almost unaffected by 100 μM H_2_O_2_ even after an 8-h exposure and recovered even after an 8-h exposure to 250 μM H_2_O_2_ (Fig. 1*C*, right panel). *set29*^*−/+*^ parasites exhibited high tolerance to up to 350 μM H_2_O_2_, but were unable to recover from exposure to a higher H_2_O_2_ concentration (Fig. S5*D*). The mutant parasites showed high tolerance to continued exposure to 100 μM H_2_O_2_ as well (Fig. 1*D*). Western blot analyses of isolated whole cell lysates revealed that the levels of LdSET29 did not change in either cell type in response to H_2_O_2_ exposure (Fig. 1*E*), nor was there any discernible change in the distribution pattern of the protein within the cell in response to H_2_O_2_ (Fig. S5*E*). Taken together, the data in Figures 1 and S5 demonstrated that the reduction of LdSET29 protein levels to ∼50% did not significantly impact growth and cell cycle parameters of promastigotes under normal *in vitro* conditions, nor did have any noteworthy impact on the parasite’s response to UV irradiation or HU-induced chronic stress, however, the parasites gained high tolerance to an H_2_O_2_-induced oxidizing environment.

### The *set29* gene appears to be essential for cell survival

**Figure 2:**
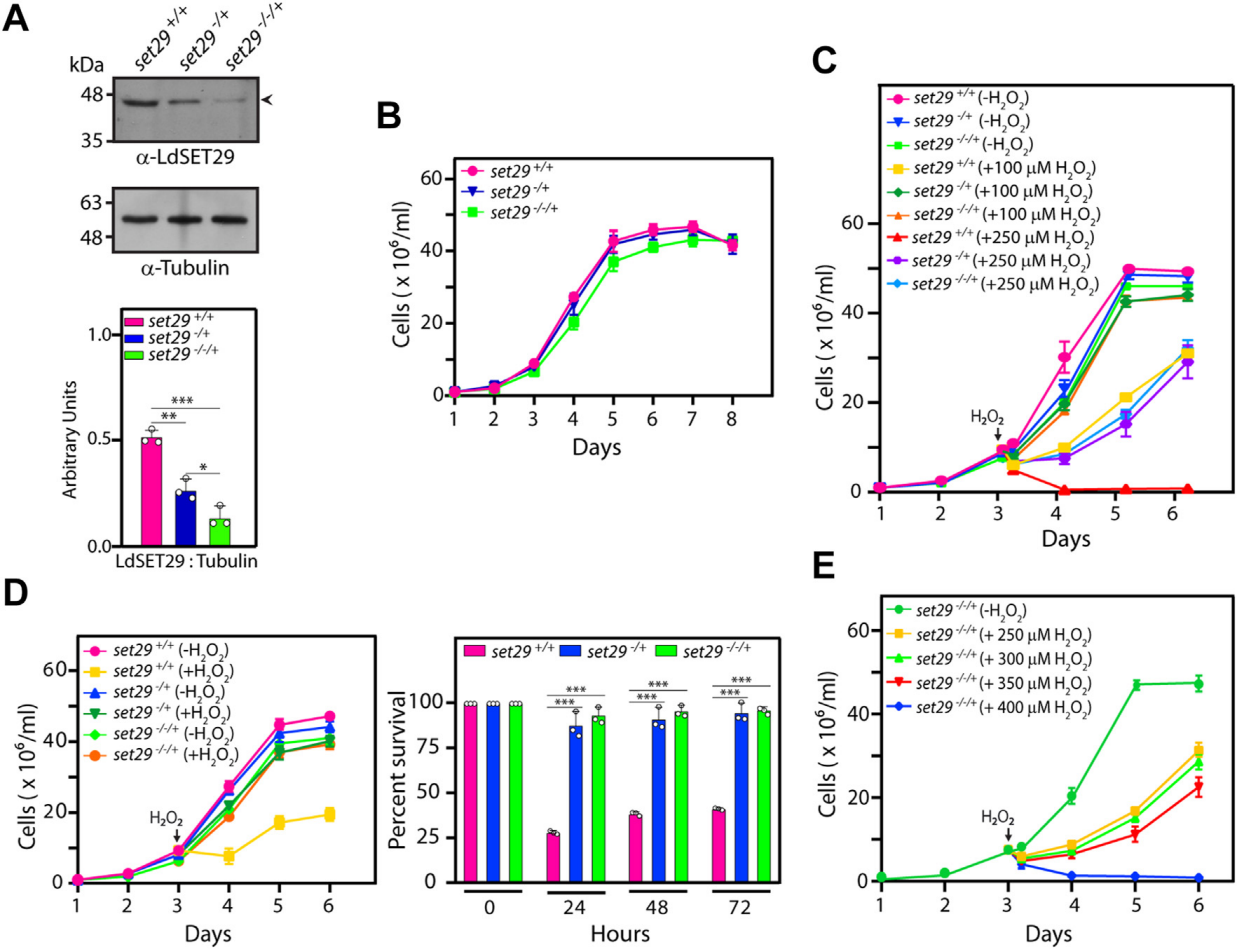
**Analysis of *set29***^***−/−/+***^**promastigotes.***A*, Western blot analysis of *Leishmania* promastigote lysates isolated from 1 × 10^8^ logarithmically growing cells, using anti-SET29 antibodies (1:1000 dilution). Tubulin served as loading control. ImageJ analysis was used for quantitation. Bar graphs represent mean values of three experiments, with error bars depicting standard deviation. Open circles on bar graphs mark values of individual experiments. Statistical significance was evaluated using two-tailed unpaired student’s *t* test. ∗∗∗ represents *p* value <0.0005, ∗∗ represents *p* value <0.005, ∗ represents *p* value <0.05. *B*, analysis of growth of parasites. The experiment was performed thrice, with two technical replicates in each biological. Values shown are mean values of the three experiments, with error bars depicting standard deviation. *C*, effect of H_2_O_2_ exposure on growth of *set29*^*−/−/+*^ promastigotes. Cultures (*set29*^*+/+*^*, set29*^*−/+*^*, set29*^*−/−/+*^ parasites) were initiated from stationary phase promastigotes, with 0 to 250 μM H_2_O_2_ being added on Day 3 (48 h after initiation). Each of the three cultures were divided into three parts at the time of addition of H_2_O_2_, with one part being continued as the untreated cells and the two other parts receiving 100 μM or 250 μM H_2_O_2_. After exposure to H_2_O_2_ for 5h, the cultures were replenished with fresh H_2_O_2_ -free M199. Cells were counted every 24 h from start of H_2_O_2_ exposure. The experiment was performed three times. Values shown are mean values of the three experiments, with error bars depicting standard deviation. *D*, effect of prolonged H_2_O_2_ exposure on growth of *set29*^*−/−/+*^ promastigotes. Cultures (*set29*^*+/+*^*, set29*^*−/+*^ and *set29*^*−/−/+*^ parasites) were initiated from stationary phase promastigotes, with 100 μM H_2_O_2_ being added on Day 3 (48 h after initiation). Each of the three cultures were divided into two parts at the time of addition of H_2_O_2_, with one part being continued as the untreated cells and the other part receiving 100 μM H_2_O_2_. H_2_O_2_-treated cultures were maintained by replenishing the H_2_O_2_ every 10 h. *Left panel*: Growth analysis. *Right panel*: Percent survival of cells in H_2_O_2_ -treated cultures in comparison with untreated cultures, calculated by dividing the number of cells in treated cultures by the number of cells in untreated cultures and multiplying by 100. The experiment was performed three times, with two technical replicates for each biological. Values shown are mean values of the three experiments, with error bars depicting standard deviation. *Open circles* on bar graphs mark values of individual experiments. Statistical significance was determined using the two-tailed unpaired student’s *t* test. ∗∗∗: *p* value <0.0005. *E*, effect of exposure to higher concentrations of H_2_O_2_ on the growth of *set29*^*−/−/+*^ promastigotes. Cultures of *set29*^*−/−/+*^ parasites were initiated from stationary phase promastigotes, with H_2_O_2_ being added on Day 3 (48 h after initiation). The culture was divided into five parts at the time of addition of H_2_O_2_, with one part being continued as the untreated cells and the other parts receiving 250 μM to 400 μM H_2_O_2_. After exposure to H_2_O_2_ for 5 h, the cultures were replenished with fresh H_2_O_2_ -free M199. Cells were counted every 24 h from the start of H_2_O_2_ exposure. The experiment was performed three times. Values shown are mean values of the three experiments, with error bars depicting standard deviation. *set29*^*+/+*^ cells: Ld1S::hyg cells. *set29*^*−/*+^ cells: *set29-*heterozygous knockout cells with one genomic allele replaced with the hygromycin resistance cassette. *set29*^*−/−/+*^cells: *set29* mutant cells with two *set29* alleles knocked out but still carrying the *set29* gene.

The second genomic allele in *set29*^*−/+*^*::hyg* was legitimately replaced with a neomycin resistance cassette (Fig. S6). While authentication at the 5′ and 3′ ends revealed that both genomic alleles had been replaced accurately, we found to our surprise that the *set29* gene had not been eliminated from the cells (Fig. S6, gel of *set29* PCR), suggesting that an additional copy of the *set29* gene was present in the genome. This finding was in synchrony with the fact that the genome of this parasite is plastic, with all 36 chromosomes having the capacity to become aneuploid (5, 27, 28). Western blot analysis of whole cell lysates isolated from these cells (named *set29*^*−/−/+*^) revealed that LdSET29 expression was ∼ 30% that in wild type cells (Fig. 2*A*). Our repeated efforts to create a true *set29*-null using either the *set29*^−/+^ or *set29*^−/−/+^ cells as the starting background strain failed (we attempted to replace the remaining *set29* gene in *set29*^−/−/+^ cells with a blasticidin resistance cassette), suggesting that this gene may be essential for cell survival.

The *set29*^*−/−/+*^ promastigotes grew at almost the same rate as the *set29*^*−/+*^ and *set29*^*+/+*^ promastigotes in normal *in vitro* cultures (Fig. 2*B*), and exhibited a response similar to *set29*^*−/+*^ promastigotes upon 5 h exposures to 100 or 250 μM H_2_O_2_ (Fig. 2*C*) as well as upon continued exposure to 100 μM H_2_O_2_ (Fig. 2*D*). *set29*^*−/−/+*^ parasites displayed high tolerance to H_2_O_2_ exposure up to concentrations of 350 μM for 5 h, but could not recover from exposure to higher concentrations of H_2_O_2_ (Fig. 2*E*). Collectively, the data in Figures 1 and 2 indicated that while ∼30% of the usual LdSET29 levels (as seen in *set29*^*−/−/+*^) was adequate for normal *in vitro* growth and propagation of promastigotes, depletion of LdSET29 to ∼50% of the usual levels (as in *set29*^*−/+*^) was sufficient to have a significant bearing on the parasite’s response to an oxidative milieu, and this response was not heightened upon further depletion of the protein.

### High tolerance of LdSET29-depleted promastigotes to an H_2_O_2_ -induced oxidative environment is coupled to the absence of detectable DNA damage

**Figure 3:**
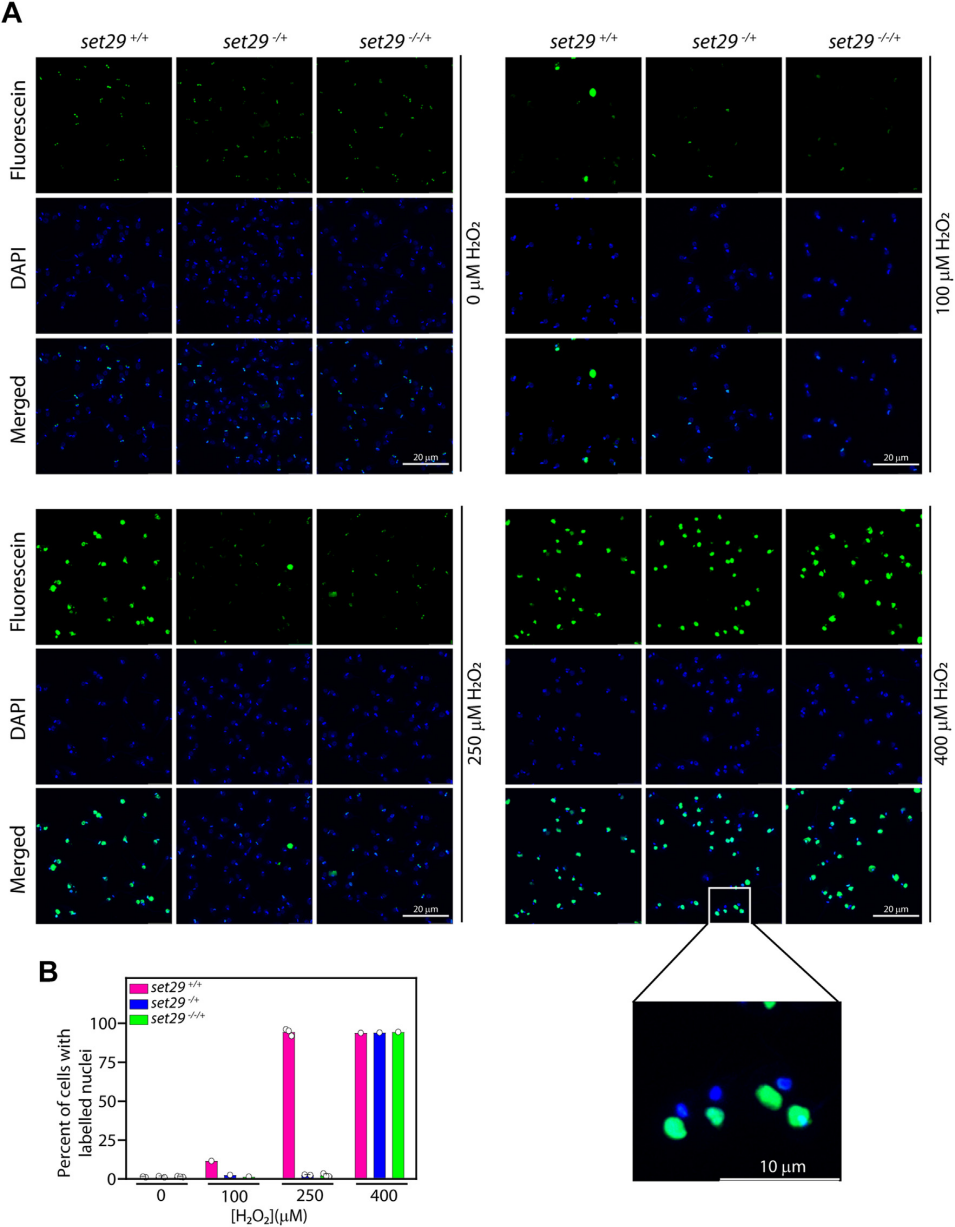
**Analysis of DNA damage in *set29* mutant parasites in response to H**_**2**_**O**_**2**_**exposure.** Cultures (*set29*^*+/+*^*, set29*^*−/+*^ and *set29*^*−/−/+*^ parasites) were initiated from stationary phase promastigotes, with 100 to 400 μM H_2_O_2_ being added on Day 3 (48 h after initiation). TUNEL assay reactions were performed on the cells after a 5 h exposure to H_2_O_2_. *A*, reactions were analysed microscopically by observing cells using a 100× (in oil) objective and capturing images of Z-stacks using the Leica TCS SP8 confocal microscope. Images were analysed using the LAS X software. DAPI: stains nucleus and kinetoplast. Fluorescein: labels the free 3′OH at DNA ends created by strand breaks. Magnification bar: 20 μm. *B*, analysis of cells carrying fluorescein-labelled nuclei in the experimental data acquired above. Between 110 to 170 promastigotes of each type were analyzed, and the percent of total cells carrying labeled nuclei was calculated. *Open circles* on bar graphs mark values of individual experiments. *set29*^*+/+*^ cells: Ld1S::hyg cells. *set29*^*−/*+^ cells: *set29-*heterozygous knockout cells with one *set29* allele knocked out. *set29*^*−/−/+*^cells: *set29* mutant cells with two *set29* alleles knocked out.

H_2_O_2_-induced genotoxic stress causes the formation of oxidative lesions and strand breaks in DNA. When TUNEL assays were carried out to examine the extent of DNA damage in promastigotes that had been exposed to H_2_O_2_ for 5 h, it was observed that while nuclei of untreated cells remained by-and-large undamaged in all three cell types analyzed (*set29*^*+/+*^*, set29*^*−/+*^ and *set29*^*−/−/+*^), following exposure to 100 μM H_2_O_2_ ∼10 to 15% *set29*^*+/+*^ nuclei were strongly labelled with fluorescein-dUMP while nuclei of *set29* mutant cells remained primarily unlabeled (Fig. 3, *A* and *B*). The difference between *set29*^*+/+*^ cells and LdSET29-depleted cells was starkly evident upon exposure to 250 μM H_2_O_2_: while ∼90 to 95% *set29*^*+/+*^ cells exhibited nuclear DNA damage, *set29* mutants showed no evidence of DNA damage (Fig. 3, *A* and *B*). However, upon exposure to 400 μM H_2_O_2,_ *set29* mutants also displayed severe DNA damage.

**Figure 4:**
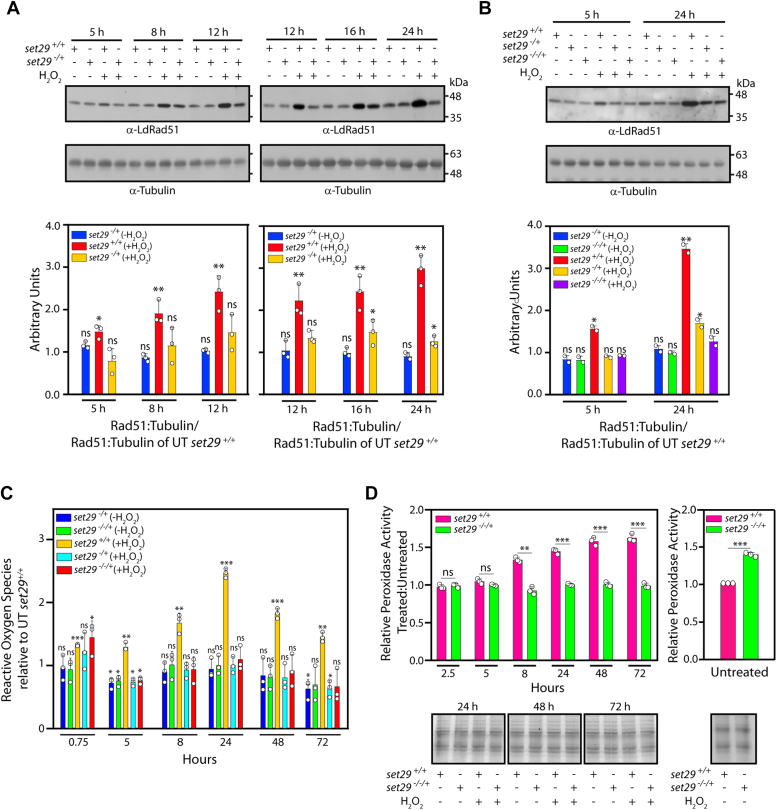
**Effectof SET29 depletion on DNA damage response and ROS production.***A* and *B*, analysis of effects of H_2_O_2_ exposure on DNA damage response in *set29* mutant parasites. Promastigote cultures were seeded from stationary phase cultures, and 48 h later the cultures were split into two, with half the cultures being continued as untreated cells, and the other half of the cultures being exposed to 100 μM H_2_O_2_ for 5 h before refeeding the cells with fresh H_2_O_2_ -free medium. Whole cell lysates were isolated from 4 × 10^7^ cells at various times thereafter, and analyzed by western blotting using anti-RAD51 antibodies ((22), 1:1000 dilution). Tubulin served as loading control. Indicated times refer to number of hours after start of H_2_O_2_ exposure. At each time, lysates were isolated from untreated as well as treated cells. The experiments were done thrice. ImageJ analysis was used for quantitation. RAD51:Tubulin ratios of the treated wild type, and untreated as well as treated *set29* mutants, were normalized to the RAD51:Tubulin of untreated wild type cells. UT: untreated. Bar graphs in *A*, represent mean values of three experiments, while bar graphs– in *B*, represent mean values of two experiments. *Open circles* on bar graphs mark values of individual experiments. Error bars depict standard deviation. Statistical significance was evaluated using two-tailed unpaired student’s *t* test. ∗∗: *p* value <0.005, ∗: *p* value <0.05, ns: statistically not significant. *A*, analysis of *set29*^*−/*+^ cells. *B*, analysis of *set29*^*−/−/+*^cells. *C*, analysis of ROS accumulation in H_2_O_2_-treated wild type, and untreated as well as treated *set29* mutant parasites, relative to untreated wild type parasites. Promastigote cultures (*set29*^*+/+*^, *set29*^*−/*+^, and *set29*^*−/−/+*^) were initiated as above, and cultures split into two 48 h later, where half of each culture was left untreated and the other half was exposed to 100 μM H_2_O_2_ for 45 min before refeeding with fresh H_2_O_2_-free medium. Cells were sampled for DCFDA assay at different times thereafter. The indicated times refer to time after start of H_2_O_2_ exposure. At each time, both untreated and treated cells were sampled. The experiment was done thrice, with technical duplicates of all samples in each experiment. Bar graphs represent mean values of three experiments, with error bars depicting standard deviation. *Open circles* on bar graphs mark values of individual experiments. Statistical significance was evaluated using two-tailed unpaired student’s *t* test. ∗∗∗: *p* value <0.0005, ∗∗: *p* value <0.005, ∗: *p* value <0.05, ns: statistically not significant. *D*, analysis of peroxidase activity. Promastigote cultures (*set29*^*+/+*^ and *set29*^*−/−/+*^) were initiated and exposed to H_2_O_2_ for 5 h, and cells sampled at different times for Amplex Red assay. *Upper left panel*: Activity in H_2_O_2_-treated parasites in comparison to untreated parasites. *Upper right panel*: activity in untreated *set29*^*−/−/+*^ parasites in comparison to that in untreated *set29*^*+/+*^ parasites. *Lower panels*: Coomassie-stained gel panels of cell inputs used in the reactions (input loading controls). The experiment was done thrice. Bar graphs represent mean values of three experiments, with error bars depicting standard deviation. *Open circles* on bar graphs mark values of individual experiments. Statistical significance was evaluated using two-tailed unpaired student’s *t* test. ∗∗∗: *p* value <0.0005, ∗∗: *p* value <0.005, ns: statistically not significant. *set29*^*+/+*^ cells: Ld1S::hyg cells. *set29*^*−/*+^ cells: *set29* mutant cells with one *set29* allele knocked out. *set29*^*−/−/+*^cells: *set29* mutant cells with two *set29* alleles knocked out.

In the absence of detectable DNA damage at H_2_O_2_ concentrations up to 250 μM in LdSET29-depleted cells, we investigated if *set29* mutant cells displayed an enhanced DNA damage response leading to hyper-efficient repair. As DNA double-strand breaks in *Leishmania* are primarily repaired using the HR pathway (29), we used RAD51 as a marker for analyzing the activation of the damage response. Thus, cells treated with H_2_O_2_ (100 μM) for 5 h were analyzed for RAD51 activation. We found that, contrary to an early or overactive HR-mediated DNA repair system coming into play, the activation of RAD51 in *set29* mutants was actually poorer than in *set29*^*+/+*^ cells, with modest levels of RAD51 activation only being detected 16 h after exposure to H_2_O_2_ in *set29* mutants (Fig. 4, *A* and *B*). These data reinforced the fact that downregulation of LdSET29 to ∼50% wild type levels was sufficient to trigger an aberrant parasite response to H_2_O_2_ -induced genotoxic stress in *in vitro* promastigote cultures.

### The deviant response of *set29* mutant parasites to H_2_O_2_ exposure is linked to the absence of detectable ROS activation

DNA suffers damage in response to H_2_O_2_ exposure due to oxidation of DNA bases, as well as free radical attack on the sugar-phosphate backbone by the reactive oxygen species (ROS). In the absence of detectable DNA damage in LdSET29-depleted promastigotes treated with up to 250 μM H_2_O_2_, we assessed the levels of intracellular ROS in these cells using the DCFDA assay (22, 30). This assay exploits the fact that dichlorodihydrofluorescein diacetate dye taken in by cells gets deacetylated by intracellular esterases, with the resulting dichlorodihydrofluorescein being oxidized by the cellular ROS to form dichlorofluorescein (DCF), whose production is monitored by analyzing fluorescence emission. To determine the levels of ROS activation in response to H_2_O_2_ treatment, logarithmically growing *set29* mutant parasites were incubated in H_2_O_2_ (100 μM) before refeeding the cells and sampling at various times thereafter (described in Experimental procedures). A sharp difference in the pattern of ROS production in *set29* mutant parasites exposed to H_2_O_2_ (compared to similarly exposed wild-type parasites) was observed. While wild-type parasites exhibited a steady activation of ROS up to 24 h after H_2_O_2_ exposure before a gradual decrease, *set29* mutant parasites displayed a limited ROS activation response initially, which rapidly came down to basal levels (Fig. 4*C*).

Unlike mammalian cells, trypanosomatids lack catalase and selenium-dependent peroxidases that are typically used for hydroperoxide detoxification and use an alternative scavenging system that is trypanothione-dependent. The cytosolic trypanothione peroxidase system has three primary components: trypanothione reductase (TR), tryparedoxin (TXN), and tryparedoxin peroxidase (cTxnPx: cytosolic tryparedoxin peroxidase). The concerted activities of these three components lead to the flow of reducing equivalents from NADPH, through trypanothione and tryparedoxin to tryparedoxin peroxidase, which then catalyzes the reduction of hydroperoxides, thus detoxifying the cell. In the absence of detectable induction of ROS in *set29* mutant parasites upon H_2_O_2_ exposure, we investigated the possibility of peroxidase activity being upregulated in LdSET29-depleted cells. Peroxidase activity was analyzed by treating the parasites with H_2_O_2_ (100 μM) before refeeding and sampling cells at various time intervals thereafter for performing the Amplex Red assay (described in Experimental procedures; (22, 31)). Peroxidase activity causes the Amplex Red reagent to react with H_2_O_2_ to produce the red-fluorescent resorufin. The assay revealed that, relative to untreated cells, peroxidase activity increased till up to 72 h after exposure to H_2_O_2_ in wild type parasites, but not in *set29* mutant parasites, which displayed no activation of peroxidase activity relative to untreated cells (Fig. 4*D*, left panel). On comparing levels of peroxidase activity in untreated *set29* mutant parasites *versus* wild-type parasites, however, we found peroxidase activity to be modestly higher in the mutant parasites (Fig. 4*D*, right panel), suggesting that LdSET29 depletion might perturb the regulation of peroxidase activity. The higher levels of peroxidase activity in *set29* mutant cells could thus be one of the factors contributing to a muted ROS activation response upon H_2_O_2_ exposure.

### The aberrant phenotypes associated with LdSET29 depletion are rescued by ectopic expression of LdSET29

**Figure 5:**
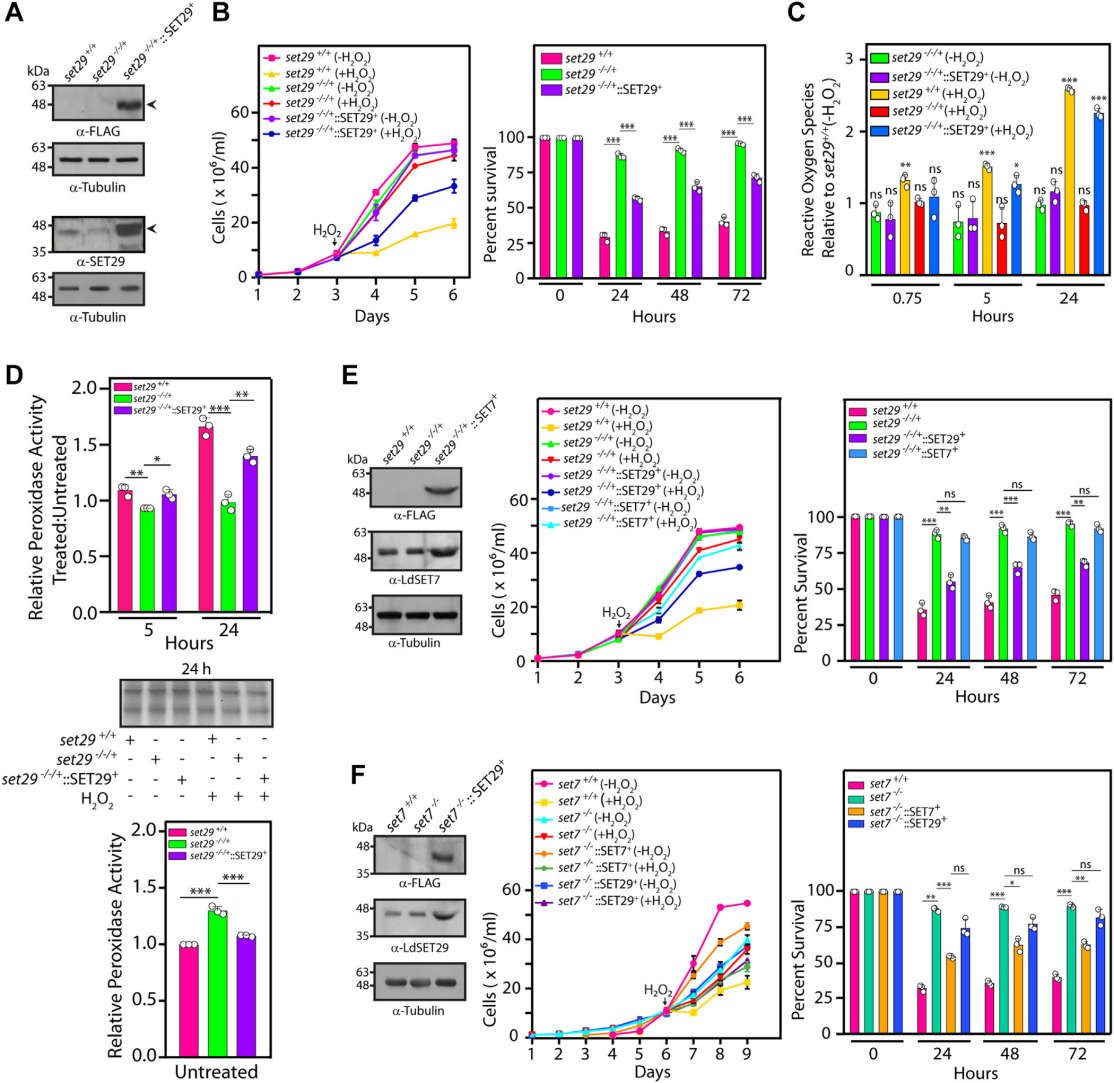
**Analysis of*****set29***^***−/−/+***^***::*****SET29**^**+**^**parasites**. *A*, Western blot analysis of *Leishmania* promastigote lysates isolated from 8 × 10^7^ logarithmically growing cells. *First panel*: Analysis using anti-FLAG antibodies (1:1000 dilution). *Third panel*: Analysis using anti-SET29 antibodies (1:1000 dilution). *Second and fourth panels*: Tubulin loading control. *B*, effect of prolonged H_2_O_2_ exposure on growth. Cultures (*set29*^*+/+*^, *set29*^*−/−/+*^ and *set29*^*−/−/+*^::SET29^+^parasites) were initiated from stationary phase promastigotes, with 100 μM H_2_O_2_ being added on Day 3 (48 h after initiation). At the time of addition of H_2_O_2_ each culture was divided into two parts, with one part being continued as the untreated cells and the other part receiving 100 μM H_2_O_2_. H_2_O_2_-treated cultures were maintained by replenishing the H_2_O_2_ every 10 h. *Left panel*: Growth analysis. *Right panel*: Percent survival of cells in H_2_O_2_ -treated cultures in comparison with untreated cultures, calculated by dividing the number of cells in treated cultures by the number of cells in untreated cultures and multiplying by 100. The experiment was performed three times, with two technical replicates for each biological. Values shown are mean values of the three experiments, with error bars depicting standard deviation. *Open circles* on bar graphs mark values of individual experiments. Statistical significance was determined using the two-tailed unpaired student’s *t* test. ∗∗∗: *p* value <0.0005, ns: not significant. *C*, analysis of ROS accumulation. ROS levels were determined in H_2_O_2_ -treated wild type, and untreated as well as treated *set29* mutant and rescue cells, relative to untreated wild type cells, as detailed in the legend of Figure 4*C*. UT: untreated. The experiment was done thrice, with technical duplicates of all samples in each experiment. Bar charts represent data that is the average of three experiments. *Open circles* on bar graphs mark values of individual experiments. Error bars depict standard deviation, and statistical significance was determined using the two-tailed unpaired student’s *t* test. ∗∗∗: *p* value <0.0005, ∗∗: *p* value <0.005, ∗: *p* value <0.05, ns: statistically not significant. *D*, analysis of peroxidase activity. Promastigote cultures (*set29*^*+/+*^, *set29*^*−/−/+*^ and *set29*^*−/−/+*^::SET29^+^) were initiated and exposed to H_2_O_2_ for 5 h, and cells sampled at different times for Amplex Red assay. *Upper panel*: Activity in H_2_O_2_-treated parasites in comparison to untreated parasites. *Middle panel*: Coomassie-stained gel panels of cell inputs used in the reactions (input loading controls). *Lower panel*: activity in untreated *set29*^*−/−/+*^ or *set29*^*−/−/+*^::SET29^+^ parasites in comparison to that in untreated *set29*^*+/+*^ parasites. The experiment was done thrice. Bar graphs represent mean values of three experiments, with error bars depicting standard deviation. *Open circles* on bar graphs mark values of individual experiments. Statistical significance was evaluated using two-tailed unpaired student’s *t* test. ∗∗∗: *p* value <0.0005, ∗∗: *p* value <0.005, ∗: *p* value <0.05. *E* and *F*, analysis of genetic interactions between *set29* and *set7. E*, *Left panel*: Western blot analysis of whole cell lysates isolated from 8 × 10^7^ logarithmically growing promastigotes, probed with anti-FLAG (1:1000) and anti-SET7 (1:2500) antibodies. *Middle* and *right panels*: Analysis of growth and survival of promastigotes after prolonged exposure to 100 μM H_2_O_2._ Cultures (*set29*^*+/+*^, *set29*^*−/−/+*^, *set29*^*−/−/+*^::SET29^+^ and *set29*^*−/−/+*^::SET7^+^ parasites) were treated with 100 μM H_2_O_2_, as earlier. H_2_O_2_-treated cultures were maintained by replenishing the H_2_O_2_ every 10 h. *Middle panel*: Growth analysis. *Right panel*: Percent survival of cells in H_2_O_2_ -treated cultures in comparison with untreated cultures *F*, *Left panel*: Western blot analysis of whole cell lysates isolated from 8 × 10^7^ logarithmically growing promastigotes, probed with anti-FLAG (1:1000) and anti-SET29 (1:1000) antibodies. *Middle* and *right panels*: Analysis of growth and survival of promastigotes after prolonged exposure to 100 μM H_2_O_2_. Cultures (*set7*^*+/+*^, *set7*^*−/−*^, *set7*^*−/−*^::SET7^+^ and *set7*^*−/−*^::SET29^+^ parasites) were treated with 100 μM H_2_O_2_, as earlier. H_2_O_2_-treated cultures were maintained by replenishing the H_2_O_2_ every 10 h. *Middle panel*: Growth analysis. *Right panel*: Percent survival of cells in H_2_O_2_ -treated cultures in comparison with untreated cultures. The experiments in *E* and *F* were performed three times, with two technical replicates for each biological. Values shown are mean values of the three experiments, with error bars depicting standard deviation. *Open circles* on bar graphs mark values of individual experiments. Statistical significance was determined using the two-tailed unpaired student’s *t* test. ∗∗∗: *p* value <0.0005, ∗∗: *p* value <0.005, ∗: *p* value <0.05, ns: not significant. *set29*^*+/+*^ cells: Ld1S::hyg cells. *set29*^*−/−/+*^cells: *set29* mutant cells with two *set29* alleles knocked out. *set29*^*−/−/+*^*::*SET29^+^ cells: *set29*^*−/−/+*^ cells expressing LdSET29 episomally. *set29*^*−/−/+*^*::*SET7^+^ cells: *set29*^*−/−/+*^ cells expressing LdSET7 episomally. *set7*^*+/+*^ cells: Ld1S::neo-hyg cells. *set7*^*−/−*^ cells: *set7*-nulls. *set7*^*−/−*^::SET7^+^ cells: *set7*-nulls expressing SET7-FLAG episomally. *set7*^*−/−*^::SET29^+^ cells: *set7*-nulls expressing SET29-FLAG episomally.

To confirm that the characteristics of LdSET29-depleted cells were not due to secondary effects of mutations that may have occurred elsewhere in the genome we expressed LdSET29-FLAG episomally in *set29*^*−/−/+*^ cells (Fig. 5*A*) and monitored the growth of these parasites in an H_2_O_2_-induced oxidative milieu. A partial rescue of the growth phenotype was obtained (Fig. 5*B*). Likewise, when ROS activation and peroxidase activity in response to H_2_O_2_ exposure was analyzed, partial rescues were evident (Fig. 5, *C* and *D*). While the reasons for obtaining a partial rescue were not certain, a possible cause could be variability in the expression of LdSET29-FLAG in the different cells of the population. These data suggested that the phenotypes of LdSET29-depleted cells were genuinely attributable to the knockout of the *set29* genes.

### LdSET29 and LdSET7 proteins interact with each other *in vitro* as well as *in vivo*

The behavior of *set29* mutant parasites in H_2_O_2_-induced oxidizing environment strongly resembled that of *set7* mutant parasites, which also demonstrated higher tolerance to oxidative stress, displaying a muted ROS activation response to H_2_O_2_ exposure coupled to modestly higher basal peroxidase activity (22). This led us to consider the possibility of LdSET7 and LdSET29 working together to temper the parasite’s response to an oxidative milieu. To study genetic interactions between *set7* and *set29* we overexpressed the LdSET7 protein in *set29*^*−/−/+*^ cells, and the LdSET29 protein in *set7*-nulls, to assess if either protein was able to rescue the aberrant phenotype associated with the knockout of the other gene. However, when *set29*^*−/−/+*^ parasites overexpressing LdSET7 (Fig. 5*E*, left panel) and the *set7-*null parasites overexpressing LdSET29 (Fig. 5*F*, left panel) were subjected to prolonged H_2_O_2_ exposure and their growth and survival monitored, in neither case was a rescue of the mutant phenotype detectable (Fig. 5, *E* and *F*, middle and right panels).

**Figure 6:**
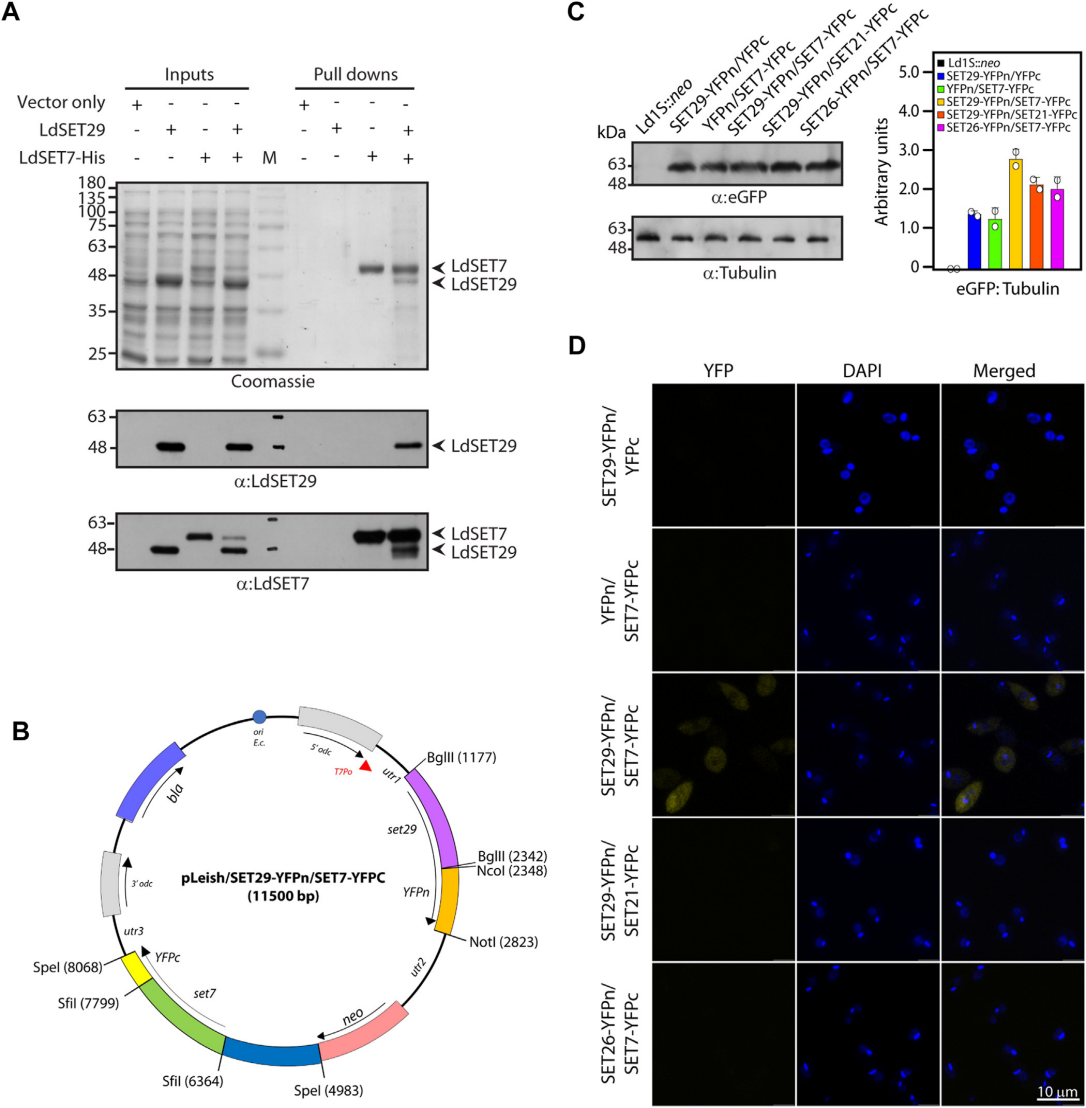
**Analysis of physical interactions between LdSET29 and LdSET7.***A*, analysis of pulldowns carried out from *E*. *coli* lysates expressing LdSET29 (non-tagged) and LdSET7 (His-tagged) using cobalt affinity beads. The bead-bound fractions were eluted in 250 mM imidazole. 1/20 of the eluates were analyzed in Coomassie-stained gels. 1/200 of the eluates were analyzed in western blots. The blot was first probed with anti-SET29 antibodies (1:2500 dil) and then with anti-SET7 antibodies (1:2500 dil). The experiment was done three times and one data set is shown here. *B*, schematic outline of pLeish/YFPn/YFPc carrying *set29* and *set7* genes. *C*, Western blot analysis of whole cell lysates isolated from 1.5 × 10^8^ logarithmically growing transfectant promastigotes harboring various BiFC plasmids, using anti-GFP antibodies (1:2000 dil; Invitrogen Cat. No. CAB4211). ImageJ analysis was used for quantitation. Bar graphs represent mean values of two experiments, with error bars depicting standard deviation. *Open circles* on bar graphs mark values of individual experiments. *D*, analysis of *in vivo* interactions between LdSET29 and LdSET7 using BiFC. Bimolecular fluorescence was analyzed by excitation at 513 nm and monitoring emission at 527 nm. Cells were observed with a 100× (in oil) objective using a Leica TCS SP8 confocal microscope. Images were captured by Z-stacking and analyzed using LAS X software. Magnification bar: 10 μm. Ld1S:neo: Ld1S cells carrying *neo*^r^ gene. SET29-YFPn/YFPc: Transfectants harboring the BiFC vector with LdSET29 expressed in fusion with the N-terminal fragment of YFP. YFPn/SET7-YFPc: Transfectants harboring the BiFC vector, with LdSET7 expressed in fusion with the C-terminal fragment of YFP. SET29-YFPn/SET7-YFPc: Transfectants harboring the BiFC vector, with LdSET29 expressed in fusion with the N-terminal fragment of YFP and LdSET7 expressed in fusion with the C-terminal fragment of YFP. SET29-YFPn/SET21-YFPc: Transfectants harboring the BiFC vector, with LdSET29 expressed in fusion with the N-terminal fragment of YFP and LdSET21 expressed in fusion with the C-terminal fragment of YFP. SET26-YFPn/SET7-YFPc: Transfectants harboring the BiFC vector, with LdSET26 expressed in fusion with the N-terminal fragment of YFP and LdSET7 expressed in fusion with the C-terminal fragment of YFP.

To investigate the possibility of the two proteins physically interacting with each other to work in a heteromeric partnership, the *set7* and *set29* genes were expressed in *E. coli* cells using the pET-Duet vector, such that LdSET7 was expressed with a hexa-His N-terminal tag while LdSET29 was expressed without any tag. LdSET7 was pulled down from extracts of these *E. coli* cells using cobalt affinity beads, and the pulldown fraction was analyzed by Coomassie-staining of SDS-PAGE as well as by Western blotting (described in Experimental procedures). As apparent in Figure 6*A*, LdSET29 was pulled down along with LdSET7, with the interaction being detected by Coomassie staining and affirmed by western blotting. To evaluate if the two proteins interacted with each other in *Leishmania* cells we resorted to carrying out bimolecular fluorescence complementation (BiFC) assays. The assay is based on the principle that Yellow Fluorescent Protein (YFP) can be split into two fragments which do not individually fluoresce, but which can complement each other through a physical association of the two fragments, leading to emission of yellow fluorescence. The physical association of the two fragments would only be brought about, if the two proteins they are fused with interact with each other. The BiFC vector for use in *Leishmania* parasites, pLeish/YFPn/YFPc, was constructed as described in Methods (Fig. S7), and the *set7* and *set29* genes cloned such that LdSET7 would be expressed in fusion with the C-terminal fragment (YFPc) and LdSET29 would be expressed in fusion with the N-terminal fragment (YFPn) (Fig. 6*B*). The plasmid constructs were transfected into *Leishmania* promastigotes, and Western blot analyses of whole cell lysates carried out with anti-GFP antibodies to screen transfectant promastigotes for expression of the different fusion proteins (Fig. 6*C*). When the transfectant promastigotes were observed microscopically, while none of the control cells demonstrated BiFC (Figs. 6*D* and S8*A*), cells harboring the pLeish/SET29-YFPn/SET7-YFPc plasmid displayed yellow fluorescence, signifying an interaction between LdSET29 and LdSET7 (Fig. 6*D*). The degree of BiFC varied across the cells of the population, reflecting variations in extent of the interaction from cell to cell. Contrastingly, transfectant promastigotes harboring either pLeish/SET29-YFPn/SET21-YFPc or pLeish/SET26-YFPn/SET7-YFPc did not demonstrate BiFC (Fig. 6*D*), signifying that the detected interaction of LdSET7 and LdSET29 was genuine. Fluorescence was visible primarily in the cytosol, in keeping with the fact that while LdSET29 is ubiquitous in the cell, LdSET7 is predominantly cytosolic (22). The strength of the interaction remained variable across the cells of the population even in response to H_2_O_2_ exposure, with no ostensible increase in fluorescence (data not shown). Considering these results, we concluded that LdSET29 and LdSET7 may work in collaboration to moderate the cell’s response to an oxidative milieu.

### Analysis of transcriptomes of *set29*^*−/−/+*^ and *set7*^*−/−*^ promastigotes challenged by oxidative stress

**Figure 7:**
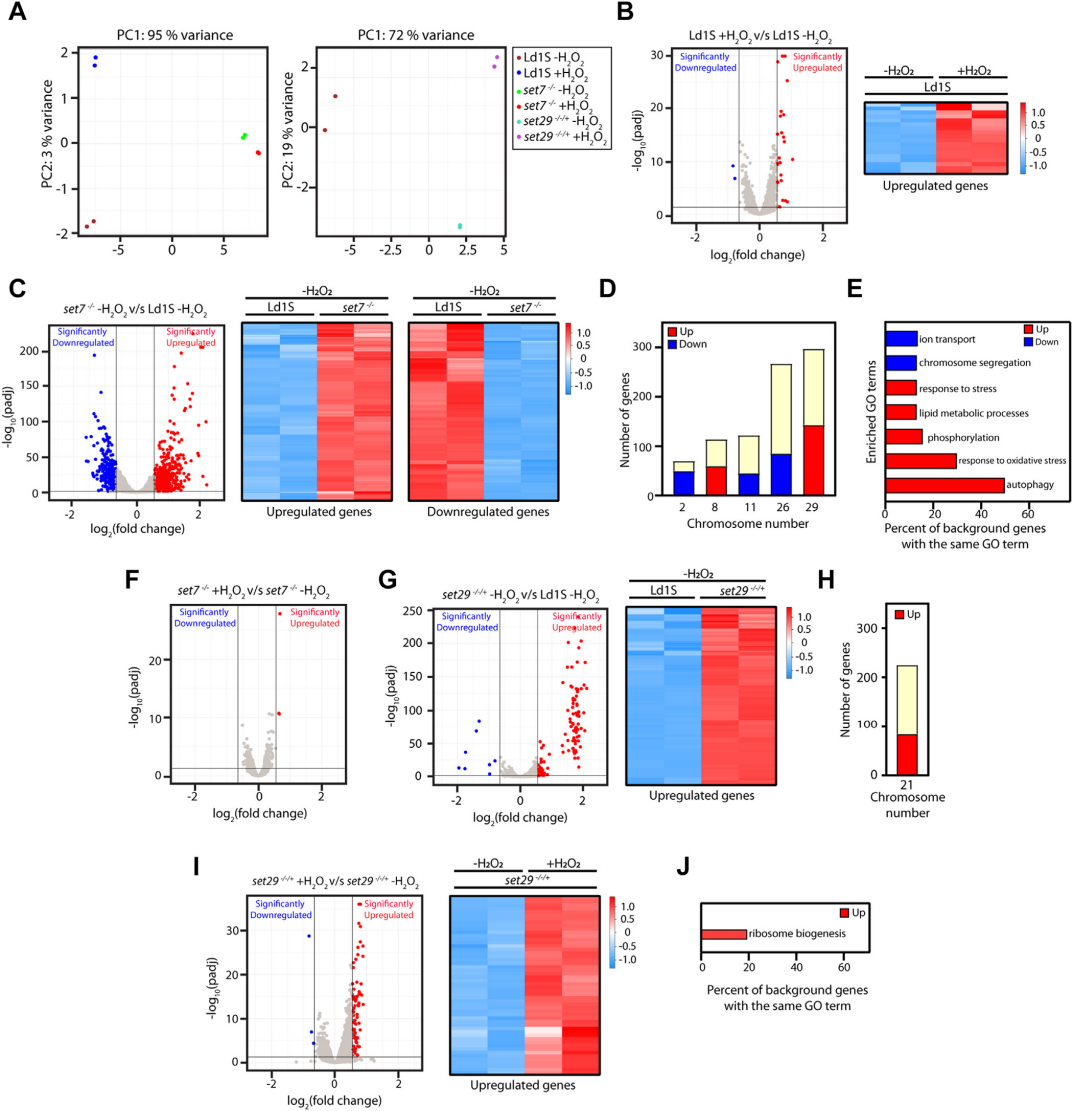
**Analysis of transcriptomes of *set7* and *set29* mutant parasites by RNA-seq.***A*, principal Component (PC) analysis of datasets obtained from RNA-seq. *Left panel*: Analysis of first experimental set. *Right panel*: Analysis of second experimental set. *B*, *left panel*: Volcano plot depicting differentially expressed genes (DEGs) in wild type parasites exposed to H_2_O_2_*versus* untreated parasites. *Blue*: downregulated genes. *Red*: upregulated genes. padj <0.05, log_2_ fold change ≤−0.7 used as cut-off for significant downregulation and ≥0.6 used as cut-off for significant upregulation. *Right panel*: Heat map showing the row-wise deviation from average expression of the DESeq2 normalized counts for genes upregulated in H_2_O_2_-treated cells. Colour key indicates fold change relative to mean of all samples. *C*, *left panel*: Volcano plot depicting differentially expressed genes (DEGs) in *set7*-nulls *versus* wild type parasites (both untreated). *Blue*: downregulated genes. *Red*: upregulated genes. padj <0.05, log_2_ fold change ≤−0.7 used as cut-off for significant downregulation and ≥0.6 used as cut-off for significant upregulation. *Middle and right panels*: Heat maps showing the row-wise deviation from average expression of the DESeq2 normalized counts for genes upregulated (*middle panel*) and downregulated (*right panel*) in *set7*-nulls. Colour keys indicates fold change relative to mean of all samples. *D*, bar graph depicting chromosomes carrying large clusters of downregulated (*blue*) and upregulated (*red*) genes in *set7*-nulls compared to wild type parasites. Bars represent total number of genes in the chromosomes, with *blue* and *red* representing the number of downregulated and upregulated genes. *E*, analysis of DEGs in *set7*-nulls compared to wild type parasites using GO enrichment. Bar graphs depict DEGs (*blue*: downregulated, *red*: upregulated) that are categorized by a GO term, as a percent of the total genes in the organism that are categorized by that term. *F*, Volcano plot depicting differentially expressed genes (DEGs) in *set7*-nulls exposed to H_2_O_2_*versus* untreated *set7*-nulls. *Red*: upregulated genes. padj <0.05, log_2_ fold change ≥0.6 used as cut-off for significant upregulation. *G*, *left panel*: Volcano plot depicting differentially expressed genes (DEGs) in *set29* mutants *versus* wild type parasites (both untreated). *Blue*: downregulated genes. *Red*: upregulated genes. padj < 0.05, log_2_fold change ≤−0.7 used as cut-off for significant downregulation and ≥ 0.6 used as cut-off for significant upregulation. *Right panel*: Heat map showing the row-wise deviation from average expression of the DESeq2 normalized counts for genes upregulated in *set29* mutant parasites. Colour key indicates fold change relative to mean of all samples. *H*, bar graph depicting chromosome carrying large clusters of upregulated (*red*) genes. Bar represents total number of genes in the chromosome, with *red* representing the number of upregulated genes. *I*, *left panel*: Volcano plot depicting differentially expressed genes (DEGs) in *set29* mutants exposed to H_2_O_2_*versus* untreated *set29* mutants. *Blue*: downregulated genes. *Red*: upregulated genes. padj <0.05, log_2_ fold change ≤−0.7 used as cut-off for significant downregulation and ≥0.6 used as cut-off for significant upregulation. *Right panel*: Heat map showing the row-wise deviation from average expression of the DESeq2 normalized counts for genes upregulated in *set29* mutant parasites treated with H_2_O_2_. Colour key indicates fold change relative to mean of all samples. *J*, analysis of DEGs in *set29* mutants challenged with H_2_O_2_ exposure compared to untreated *set29* mutants using GO enrichment. Bar graph depicts upregulated DEGs that are categorized by a GO term, as a percent of the total genes in the organism that are categorized by that term.

While the two proteins may work together at least in part, although *set7* is not essential to the cell *set29* appears to be–suggesting that they must have divergent functions as well. Towards examining these aspects, we analyzed global gene expression in wild type *set7*-null and *set29*^*−/−/+*^ promastigotes. Furthermore, we examined the effect of H_2_O_2_ - induced oxidative stress on global gene expression in all three parasite types. Two sets of experiments, each with two biological replicates, were carried out. The first set of experiments included wild type and *set7*-nulls (untreated and H_2_O_2_-treated) while the second set of experiments included wild type and *set29*^*−/−/+*^ parasites (untreated and H_2_O_2_-treated). Principal component analysis revealed that in the first set of experiments the data of two biologicals of a parasite and treatment type were suitably clustered in all instances (Fig. 7*A*, left panel). However, in the second set of experiments the data of the two biologicals of H_2_O_2_-treated wild type parasites displayed high divergence and this dataset was excluded from further analysis (Fig. 7*A*, right panel). The data were analyzed against the annotated LdBPK282A1 genome sequence available in the Tritryp database (www.tritrypdb.org). Reads were found to map against a total of 8135 genes in all samples. Low abundance reads (<200 for a gene across all samples of an experimental set) and short reads (<25 nt) were eliminated from the analysis. In comparing data sets to identify differentially expressed genes (DEGs) we applied a cut-off of an average log_2_ fold change of ≤ −0.7 for evaluating downregulation of gene expression and an average log_2_ fold change of ≥0.6 for evaluating upregulation of gene expression, with a padj cut-off <0.05.

On comparing H_2_O_2_-treated with untreated wild-type parasites, it was found that only ∼20 genes were differentially regulated (Fig. 7*B*). As mentioned earlier, the *Leishmania* genome is organized unusually, with large numbers of functionally unrelated genes being clustered unidirectionally. The clusters are typically coordinately transcribed polycistronically, followed by trans-splicing of a short leader sequence at the 5′ end and polyadenylation at the 3′ end of individual coding units in the transcripts. As the ∼20 DEGs were scattered throughout the genome, their regulation was most likely post-transcriptional. None of the genes in the trypanothione peroxidase pathway were upregulated in response to H_2_O_2_ exposure. This was in keeping with our earlier findings showing the same by real-time PCR analyses of the genes of this pathway (22), thus indicating that the previously reported activation of expression of the trypanothione peroxidase protein in promastigotes exposed to H_2_O_2_ (32) is not due to elevated transcript levels.

When comparing the transcriptome of *set7*^*−/−*^ parasites with that of wild-type parasites (untreated), it was found that elimination of the *set7* gene impacted the transcript levels of ∼1000 genes, with ∼275 genes showing a decrease and ∼710 genes showing an increase in transcript levels (Fig. 7*C*). Interestingly, the transcript levels of genes encoding all four core histones increased ∼ two-fold in *set7*-nulls. The *txnPx* gene (encoding tryparedoxin peroxidase) transcripts also increased ∼ two-fold upon *set7* elimination. These data were in keeping with our earlier findings using real-time PCR analyses of all the genes of the pathway (22), thus validating the RNA-seq results. The increase in transcript levels of these and other peroxidase-encoding genes in *set7*-nulls (genes listed under the ontology term “response to oxidative stress” in Table S1) could be the reason for the previously reported enhanced basal levels of peroxidase activity in these cells (22). We found that ∼30% of the upregulated genes lay on two chromosomes, chromosomes 8 and 29 (Figs. 7*D* and S9*A*), while ∼63% of the downregulated genes lay on three chromosomes, chromosomes 2, 11 and 26 (Figs. 7*D* and S9*B*). Other than these, the DEGs were scattered around the genome, indicating that LdSET7 did not play a role in modulating global gene transcription. Interestingly, the transcript levels of six genes encoding RNA binding proteins were elevated in *set7*-nulls (Table S1), implicating a possible role for one or more of these RBPs in modulating transcript stability. On carrying out a Gene Ontology enrichment analysis of the DEGs, we found that groups of genes that are annotated as protein phosphorylation genes, autophagy genes, and stress genes (including oxidative stress) had elevated transcript levels, while groups of genes annotated as ion transport genes and chromosome segregation genes had lower transcript levels (Fig. 7*E* and Table S1). These genes were generally scattered around the genome. The transcriptome of *set7*^*−/−*^ parasites did not alter upon exposure to H_2_O_2_ (Fig. 7*F*).

A comparison of the transcriptome of *set29*^*−/−/+*^ parasites with that of wild-type parasites (untreated) revealed that by and large there was no impact on global gene expression upon *set29* downregulation. Expression levels of ∼115 genes were enhanced (Fig. 7*G*) and most of these genes lay on chromosome 21, which carries the *set29* gene (Figs. 7*H* and S9*C*). Unlike *set7*^*−/−*^ parasites which showed no transcriptome changes in response to H_2_O_2_ exposure, *set29*^*−/−/+*^ parasites exhibited an increase in transcript levels of ∼80 genes (Fig. 7*I*) which were scattered throughout the genome. The members of the trypanothione peroxidase pathway were not among the DEGs in either untreated or treated parasites. The expression of the genes of the trypanothione peroxidase pathway was checked by real-time PCR as well and the results obtained validated the RNA-seq data (Fig. S8*B*). Gene Ontology enrichment analysis of the DEGs revealed that a group of genes annotated as genes involved in ribosome biogenesis were among those with elevated transcripts (Fig. 7*J* and Table S2).

The data in Figs. 7 and S9 as well as Tables S1 and S2, signified that LdSET7 and LdSET29 did not impact global gene expression, while also suggesting the possibility of SET7 and SET29 playing a role in specific transcriptional events at particular chromosomes (chromosomes 2, 8,11, 26, 29 in case of SET7 and chromosome 21 in case of SET29). Further, LdSET7 may control the regulation of >500 genes scattered around the genome *via* a post-transcriptional process, for example, through one or more RNA-binding proteins. The results indicated that while LdSET29 and LdSET7 may work together to moderate the parasite response to oxidative stress with each protein playing a distinctive role, they had otherwise divergent functions. While the transcriptome analysis of *set7*-nulls suggested that at least one of the ways by which LdSET7 may mediate its effect in moderating the stress response, maybe *via* control of transcript levels of genes encoding peroxidases (including the *txnPx* gene), LdSET29 did not exercise its effect through this route.

### Analysis of the genomes of *set29* and *set7* mutant promastigotes

The large number of DEGs in *set7*-nulls lying in polycistronic clusters over five chromosomes (Figs. 7*D* and S9, *A* and *B*) and in *set29*^−/−/+^ mutant lying on one chromosome (Figs. 7*H* and S9*C*) suggested the possibility of LdSET7 and LdSET29 playing a role in modulating transcriptional events on specific chromosomes. SET proteins have previously been found to have both, a stimulatory as well as a suppressive effect on gene transcription. The mammalian SMYD2 methylates p53 to repress p53-dependent transcription (33) but methylates Rb to activate E2F1-dependent transcription (34). Thus, it was possible that LdSET7 may play a role in activating transcription of genes on chromosomes 2, 11 and 26 (Fig. S9*B*) and in repressing transcription of genes on chromosomes 8 and 29 (Fig. S9*A*), through hitherto unidentified factors. Likewise, LdSET29 could be repressing transcription of genes of specific clusters on chromosome 21 (Fig. S9*C*). However, this would be an unusual finding in this organism where no consensus sequences have been identifiable at Transcriptional Start Regions (TSRs), although GT-rich elements at TSRs have been found to play a role in driving transcription in *T. brucei* (9). The transcriptional activators typically found in other eukaryotes have not been identifiable in trypanosomatids either.

**Figure 8:**
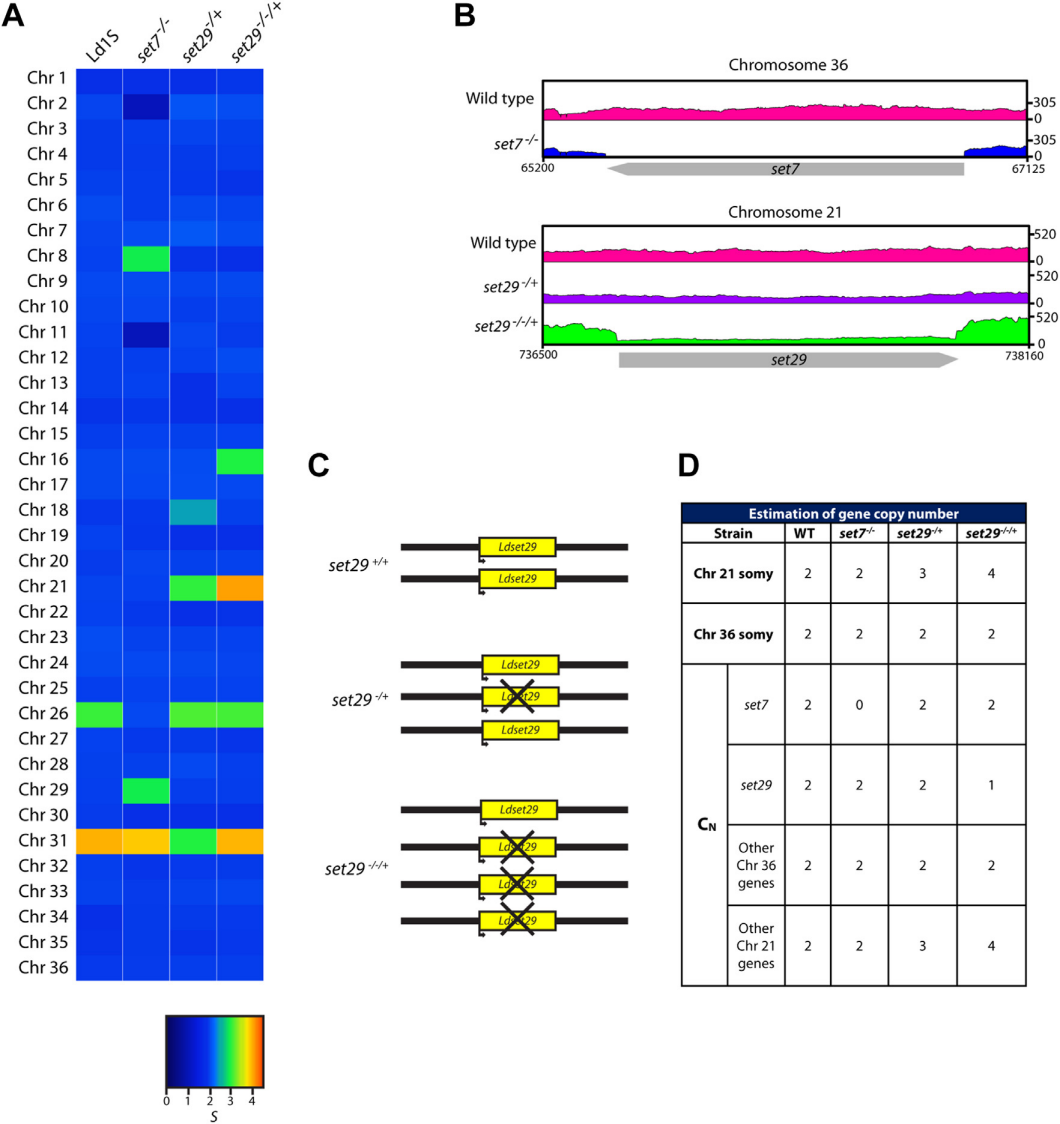
**Analysis of genomes of *set7* and *set29* mutant parasites by WGS.***A*, heat maps depicting median normalized read depths of all 36 chromosomes, of the four cell types indicated along the top of the panel. Color key indicates normalized chromosome read depths *S.* Monosomy: *dark blue*. Disomy: *light blue*. Trisomy: *green*. Tetrasomy: *orange*. *B*, *upper two panels*: *set7* sequencing tracks on chromosome 36 in wild type and *set7*-null parasites. *Lower three panels*: *set29* sequencing tracks on chromosome 21 in wild type and *set29* mutant parasites. X axis: genome window defined by the indicated positions. Y axis: read depth *C*, schematic representation of number of *set29* alleles and chromosome 21 copies in wild type and *set29* mutants. *D*, estimation of copy number of genes on chromosomes 21 and 36. Copy number of genes was determined based on chromosome somy (Fig. 8*A*) as well as the ratios arrived at in Table S3, as described in Experimental procedures.

Keeping in mind the fact that the *Leishmania* genome is plastic and known to exhibit aneuploidy, particularly in the promastigote form (5, 35), we considered the possibility of specific chromosomes presenting somy changes in the *set7* and *set29* mutants, which would account for the chromosome-specific upregulation/downregulation of gene clusters detected through RNA-seq analyses. Accordingly, the genomes of wild-type Ld1S, *set7*^−/−^, *set29*^−/+^ and *set29*^−/−/+^ promastigotes were subjected to bulk WGS, and the data were analyzed, as detailed in Methods. We found that most of the chromosomes in our laboratory Ld1S strain were disomic, with only one chromosome being trisomic: chromosome 26. One chromosome, chromosome 31, was tetrasomic (first track in heatmap of Figure 8*A*). While these observations were in keeping with earlier findings by Dumetz *et al.* (5) who also found the Ld1S chromosome 26 to be trisomic and chromosome 31 to be tetrasomic, unexpectedly, our laboratory Ld1S strain exhibited much lower chromosomal aneuploidy compared to the Ld1S strain ploidy presented by Dumetz *et al.*, where eight more chromosomes were trisomic. On comparing the methodology used, it was found that Dumetz *et al.* sequenced genomes of parasites that had undergone 22 *in vitro* passages. In using *Leishmania* parasites (wild type or mutants) for laboratory experimentation, we use parasites that have undergone 6 to 9 *in vitro* passages at the maximum. It is possible that further passaging would lead to greater aneuploidy. Considering that environmental factors also play a role in inducing aneuploidy, Dumetz *et al.* report the culturing of the parasites in M199 supplemented with 20% FCS, whereas we culture the parasites in M199 supplemented with 10% FBS. These factors could explain the differences in aneuploidy observed in our data set *versus* the data published by Dumetz *et al.*

On analyzing the genome sequence of the *set7*^*−/−*^ mutant, we found that the five chromosomes whose gene clusters were upregulated/downregulated based on RNA-seq analysis, actually presented somy changes in the *set7* mutant as compared to wild type parasites (Fig. 8*A*, second track). Gene clusters that were upregulated lay on two chromosomes, chromosomes 8 and 29 (Fig. S9*A*). Both these chromosomes (that were disomic in the wild type Ld1S) were trisomic in the *set7*^*−/−*^ parasites. Gene clusters that were downregulated lay on three chromosomes: chromosomes 2, 11 and 26 (Fig. S9*B*). All these three chromosomes underwent a reduction in somy, with chromosomes 2 and 11 now monosomic and the trisomic chromosome 26 now disomic. Thus, it appears that LdSET7 does not play a role in chromosome-specific transcriptional regulation, but rather, gene dosage is governing altered transcript levels in case of these chromosomes. As expected, the *set7* gene was absent in the mutant line (Fig. 8*B*, upper two tracks). The expansion and reduction in chromosome numbers in *Leishmania* species has been proposed to be a coping mechanism by which altered gene dosage helps the parasite overcome adverse conditions (36). While the *set7* gene was found to be inessential *set7*^*−/−*^ parasites grew much slower (22), suggesting the parasites were experiencing an internal stress. It is possible that additional aneuploidy in *set7*^−/−^ parasites favored adaptation to the loss of the *set7* gene. However, the batch of upregulated genes falling under Gene Ontology Term “response to oxidative stress” (Table S1) do not lie on the chromosomes that have expanded in number, in fact, one of them lies on the reduced chromosome 26. Thus, the phenotypes we detected with the *set7* mutant did not appear to be directly linked to alterations in gene dosage due to somy changes.

Analysis of the *set29* mutants revealed that both mutants had undergone chromosome somy changes, though these were limited in comparison to the *set7* mutant. Both *set29* mutants displayed an increase in chromosome 21 copies. While chromosome 21 (disomic in wild type Ld1S and the *set7*-null) was trisomic in *set29*^*−/+*^ (Fig. 8*A*, third track), it was tetrasomic in *set29*^−/−/+^ (Fig. 8*A*, fourth track). Chromosome 21 carries the *set29* gene, and the finding that this chromosome expanded in number as we knocked out the *set29* genomic alleles step-wise may reflect the essential nature of the SET29 protein. On analyzing the WGS data, we concluded based on the read-depth ratios (analysis method detailed in Experimental procedures; Table S3) that while the wild type (and *set7*-null mutant) harbored two alleles of the *set29* gene over two copies of chromosome 21, the *set29*^−/+^ mutant had two alleles of *set29* over the three copies of chromosome 21 and the *set29*^−/−/+^ mutant had one allele of *set29* over the four copies of chromosome 21 (Fig. 8*B*, three lower tracks, Fig. 8*C*). Contrastingly, all other similarly analyzed chromosome 21 genes had as many alleles as copies of the chromosome in the particular cell types (Figs. 8*D*, S10, and Table S3). The *set7* gene as well as other genes on chromosome 36 were also maintained at the same copy number as the number of chromosome 36 copies in each of the cell types (two copies of chromosome 36 in all cases), barring the absence of *set7* in *set7*^−/−^ cells (Table S3 and Fig. 8*D*). The expression levels of LdSET29 in *set29*^−/+^ and *set29*^−/−/+^ cells (Figs. 1*A* and 2*A*) indicated that LdSET29 protein expression levels were not directly proportionate to gene dosage: while both, wild type and *set29*^−/+^ cells have two *set29* alleles, expression in *set29*^−/+^ cells was ∼50% wild-type levels (Figs. 1*A* and 2*A*). *set29*^−/−/+^ cells, on the other hand, while harboring only one *set29* allele, expressed levels of LdSET29 ∼30% wild type levels (Fig. 2*A*). The reason for this differential gene expression in relation to the number of *set29* alleles remains unclear at this time. Interestingly, while chromosome 16 is trisomic in *set29*^−/−/+^ cells, only nine genes showed upregulation in RNA-seq data. Both *set29* mutants exhibited similar behavior in their growth and response to oxidative stress (Figure 1, Figure 2, Figure 3, Figure 4). While partial rescue of the aberrant phenotypes upon ectopic expression of the protein in *set29*^*−/−/+*^ parasites suggests that the step-wise expansion in the numbers of chromosome 21 may not directly regulate the two *set29* mutants’ differential behavior with respect to wild-type cells, the possibility of altered gene dosages due to expansion in chromosome 21 numbers impacting the behaviour of the parasite must be considered.

Taken together, the data from RNA-seq analyses and bulk WGS analyses revealed that neither LdSET7 nor LdSET29 regulate global gene expression, but LdSET7 modulates expression levels of a subset of genes, most likely post-transcriptionally. While both mutants present chromosome somy changes, the altered response to oxidizing milieu does not appear to be directly linked to these changes, as the candidate genes (which may modulate the cell’s stress response) that are upregulated in *set7* mutants do not lie on these chromosomes. In the case of *set29* mutants, the altered stress response could not be directly attributed to any differences in transcript levels. However, the possibility of altered gene dosages due to somy changes in specific chromosomes regulating the stress response in the *set* mutants cannot be ruled out.

### Depletion of LdSET29 or LdSET7 enhances parasite survival in mammalian host cells and this is coupled to decreased levels of ROS in these cells

**Figure 9:**
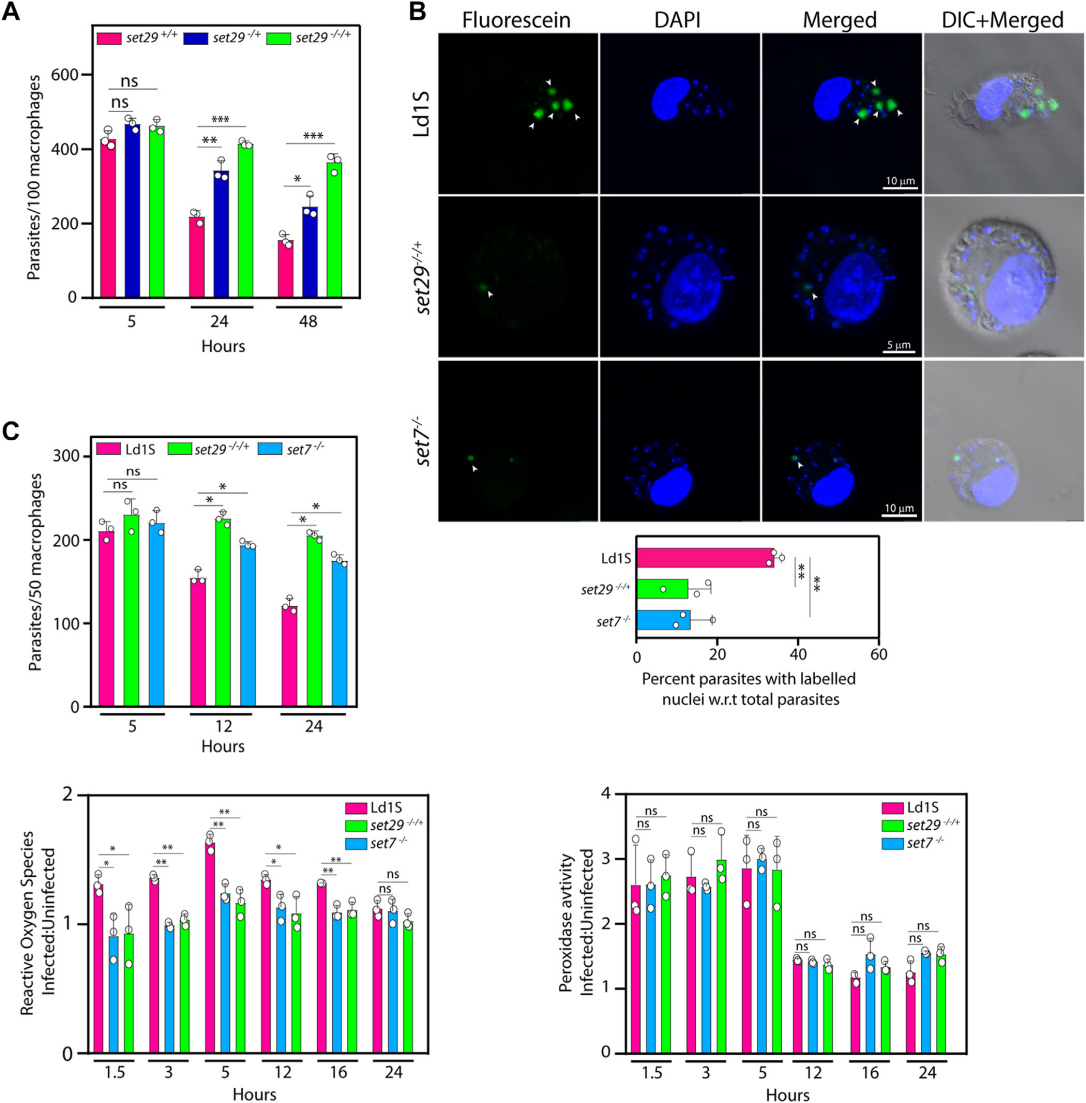
**Analysis of *set* mutant parasite-host cell interactions.***A*, analysis of survival of *set29* mutant parasites in host macrophages. Parasites were scored by counting their DAPI-stained nuclei within the host cells using Z-stack imaging in a confocal microscope. The experiment was performed thrice and average values are plotted in the bar graph. *Open circles* on bar graphs mark values of individual experiments. Error bars depict standard deviation and two-tailed unpaired student’s *t* test was used to assess statistical significance. ∗∗∗: *p* value <0.0005. ∗∗: *p* value <0.005. ∗: *p* value <0.05. ns: statistically not significant. *B*, analysis of nuclear DNA damage in intracellular parasites by TUNEL assays. The assays were performed 12 h after the start of infection. Infected macrophages were analyzed by capturing images of Z-stacks in a Leica TCS SP8 confocal microscope. Intracellular parasites were analyzed by scoring their fluorescein-labelled and DAPI-stained nuclei using Z-stack imaging, and calculating the percent of nuclei that were fluorescein-labelled. The experiment was done thrice. Bar graphs represent mean values of the three experiments, with error bars depicting standard deviation. *Open circles* on bar graphs mark values of individual experiments. Statistical significance was determined using the two-tailed unpaired student’s *t* test. ∗∗: *p* value <0.005. Types of parasites infecting the macrophages are indicated along the *left* of the figure panels. *Arrowheads* indicate fluorescein-labelled parasite nuclei. Cell sizes are indicated by magnification bars. *C*, *upper panel*: Analysis of survival of *set29* and *set7* mutant parasites in host macrophages, done as in (*A*) above. *Lower left panel*: Analysis of ROS accumulation in these infected macrophages. ROS accumulation was determined in infected host cells relative to uninfected host cells using the DCFDA assay, at various times after the start of infection. *Lower right panel*: Simultaneous analysis of peroxidase activity in infected macrophages. Peroxidase activity was determined in infected host cells relative to uninfected host cells using the Amplex Red assay, at various times after the start of infection. The experiment was done thrice, with technical duplicates of all samples in each experiment. Bar charts represent data that is the average of three experiments. *Open circles* on bar graphs mark values of individual experiments. Error bars depict standard deviation, and statistical significance was determined using the two-tailed unpaired student’s *t* test. ∗∗*p* value <0.005, ∗*p* value <0.05, ns: statistically not significant. *set29*^*+/+*^: Ld1S::hyg parasites. *set29*^*−/*+^: *set29* mutant parasites with one *set29* allele knocked out. *set29*^*−/−/+*^: *set29* mutant parasites with two *set29* alleles knocked out. *set7*^*−/−*^*: set7* mutant parasites with both *set7* alleles knocked out.

Upon *Leishmania* infection of mammalian host macrophages, the parasite is challenged by an oxidative intracellular environment. Considering the high tolerance of *set29* mutant promastigotes to *in vitro* oxidative stress, we examined the survival of these parasites within host macrophages. When the *set29* mutant parasites were infected into murine macrophages it was observed that LdSET29-depleted parasites infected the host cells comparably to wild-type cells (Fig. 9*A*, 5h time point), but survived and propagated more competently than wild type parasites thereafter (Fig. 9*A*, 24 h and 48 h time-points), with *set29*^*−/−/+*^ cells surviving more proficiently than *set29*^*−/+*^ cells. Thus, LdSET29 plays a role in regulating the parasite’s response to the hostile intracellular environment it encounters in the host.

Previous data from our lab showed that the elimination of the *set7* gene also boosted parasite survival within the host cells (22). The effect of the inhospitable intracellular environment of macrophages on parasite DNA damage was assessed 12 h after the start of infection using the TUNEL assay (detailed in Experimental procedures). As seen in Figure 9*B*, the percentage of intracellular *set7* and *set29* mutant parasites harboring damaged nuclear DNA was <half the percentage of intracellular wild-type parasites harboring the same. Thus, depletion of LdSET7 or LdSET29 apparently protected the parasites from the usual ROS response mounted by host macrophages upon *Leishmania* infection. *Leishmania* metacyclics have been reported to elicit only a measured ROS response from macrophages upon infection, with amastigotes triggering a further mitigated response. The relative magnitudes of ROS accumulated in host macrophages infected by mutant *versus* wild-type parasites (Fig. 9*C*, upper panel) were estimated using the DCFDA assay (Experimental procedures), and relative peroxidase activity was simultaneously analyzed using the Amplex Red Assay (Experimental procedures). We found ROS to be significantly higher in macrophages infected with wild type parasites as compared to those infected with *set* mutant parasites at 5h after start of infection, with ROS levels dropping thereafter (Fig. 9*C*, lower left panel). However, the differential levels of ROS accumulation in macrophages infected with mutant *versus* wild-type parasites were not attributable to higher scavenging activity in the former, with peroxidase activity in macrophages being similarly activated upon infection with wild-type, *set7* or *set29* mutant parasites (Fig. 9*C*, lower right panel). Thus, it appears that the *set7* and *set29* mutants dampen the elicited ROS response *per se* upon infection of macrophages.

While macrophages employ ROS as a protective mechanism against *Leishmania,* whose uptake triggers an oxidative burst in the host cell, one of the ways by which *Leishmania* parasites combat ROS is by inhibiting the formation of active NADPH Oxidase Complex at the phagosome membrane, thereby decreasing ROS production in the parasitophorous vacuoles within which they reside (37). While we found no evidence of either LdSET7 or LdSET29 being secreted into the host cytosol, it is possible that these proteins regulate NADPH Oxidase Complex formation indirectly by targeting one or more parasite membrane proteins, which include a metalloprotease (gp63) and a kinase (protein kinase C) that have both been found to moderate host-*Leishmania* interactions post-infection (reviewed in (38)). Such a SET7/SET29-mediated effect would be repressive, with depletion of either protein leading to enhanced activity and therefore further suppression of NADPH Oxidase Complex formation/activity. Further in-depth studies need to be carried out to investigate these possibilities.

### Identification of possible target substrates of LdSET7 and LdSET29 in *Leishmania* promastigotes

**Figure 10:**
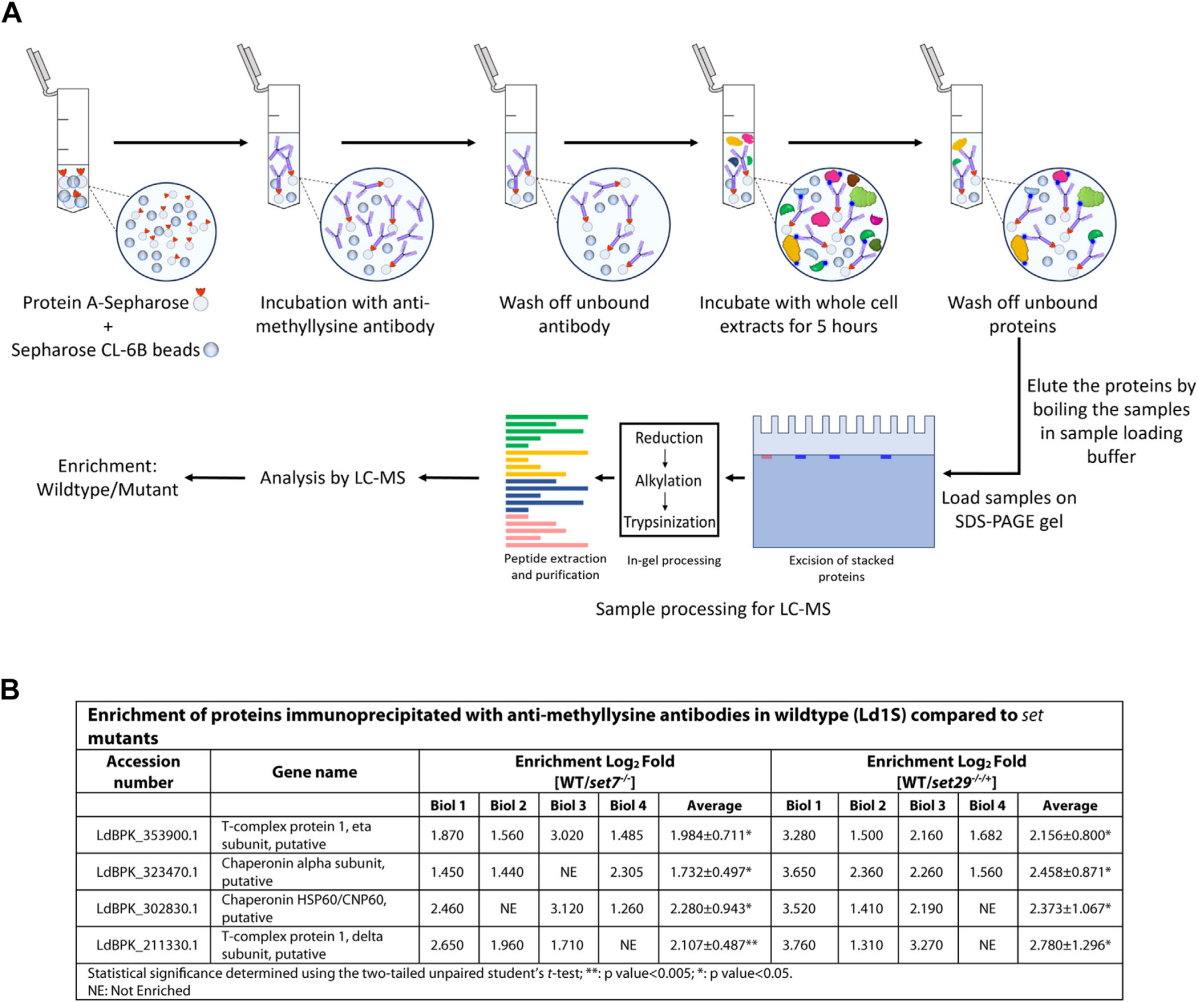
**Determination of possible target substrates.***A*, scheme of the workflow used to identify possible targets of LdSET7 and LdSET29. *B*, proteins immunoprecipitated with anti-methyllysine antibodies, that are enriched in wild type parasites over *set* mutants. Enrichment (log_2_-fold) values are presented for each biological. Statistical significance was evaluated using the two-tailed unpaired student’s *t* test. ∗∗: *p* value <0.005, ∗: *p* value <0.05. NE, not enriched.

Our data thus far implicated the increase in basal levels of peroxidase activity in the *set* mutants to be at least one of the reasons for the altered stress response in these parasites. While in *set7*-nulls this could be attributed to elevated levels of transcripts of some of the genes involved in the response to oxidative stress, this was not the case in *set29* mutants. Considering that SET domain proteins are known to mediate their effects *via* the methylation of specific lysine residues on their target proteins, and these methylations may have a stimulatory or repressive effect on the target protein’s expression/activity, we turned to working toward identifying possible target substrates of LdSET7 and LdSET29. Thus, we attempted to uncover and compare the complement of lysine-methylated proteins in wild-type and *set* mutant cells. For this, we adopted the approach of immunoprecipitating lysine-methylated proteins from lysates of wild type and *set* mutant parasites, and identifying them by mass spectrometry. Accordingly, lysates were isolated from logarithmically growing Ld1S, *set7*^*−/−*^ and *set29*^*−/−/+*^ promastigotes (cultures in mid-log phase were used), anti-methyllysine antibodies used to carry out immunoprecipitation reactions, and the immunoprecipitates subjected to tryptic digestion followed by LC-MS analyses, as detailed in Methods (Fig. 10*A*). Four separate experiments were performed, and in each experiment immunoprecipitates of all three cell types were similarly analyzed in parallel. On examining the profiles of methylated proteins immunoprecipitated from wild type (Ld1S) cells, 19 proteins were found to be immunoprecipitated in all four biologicals and 14 additional proteins were immunoprecipitated in three biologicals (Tables S4–S8). These proteins were also immunoprecipitated from *set7*^−/−^ and *set29*^−/−/+^ cells in four/three/two biologicals.

We were unable to identify any proteins that were exclusively immunoprecipitated from wild-type cell lysates in a reproducible manner. This was not entirely unexpected, taking into account the existence of multiple lysine methylation sites on proteins, with different lysines being targeted by different methylases, and different methylation events known to have different effects on protein function. In examining the enrichment of methylated proteins in immunoprecipitates from wild-type cells over immunoprecipitates from *set* mutant cells, we set an average log_2_ fold enrichment limit of ≥1.5. Thus, we identified four proteins that were enriched in wild type over both *set* mutants in either all four or three out of four biologicals (Fig. 10*B*).

Considering the phenotypes of both *set* mutants vis-à-vis response to oxidizing environment, coupled to the fact that LdSET7 and LdSET29 interact with each other, the data in Figure 10*B* suggests that T-complex protein 1 (TCP1) and Hsp60/CNP60 may be the target substrates *via* which SET7/SET29 act concertedly to mediate the parasite’s response to H_2_O_2_ exposure. Heat shock proteins are upregulated in response to environmental stress, elevated temperatures and oxidative milieu. These molecular chaperones help maintain protein homeostasis under adverse conditions (39). Previous studies have found the levels of Hsp60 to be elevated in *L. donovani* promastigotes that were exposed to H_2_O_2_ (40). The study proposed that Hsp60 plays a role in helping the parasite adapt to the altered oxidative environment, a critical aspect for supporting virulence in the mammalian host considering the intracellular macrophage milieu. This conclusion was bolstered by the findings that Hsp60 upregulation in response to H_2_O_2_-induced oxidative stress was detected only in virulent but not in avirulent promastigotes. The multi-subunit TCP1 is also known to help maintain protein homeostasis through its chaperone activity. Earlier studies have demonstrated *L. donovani* TCP1 to participate in safeguarding promastigotes from miltefosine-induced oxidative stress by mitigating ROS levels, an effect it mediates through the upregulation of trypanothione peroxidase protein expression (41). Due to the non-availability of antibodies that would react with the *Leishmania* proteins we were unable to check Hsp60 and TCP1 levels in the *set* mutants. We suggest that SET7/SET29-mediated methylation of Hsp60 and TCP1 may modulate their expression/stability/activity, thus controlling the parasite’s response to H_2_O_2_ exposure in *in vitro* promastigote cultures and in the host cell. This modulation may be perturbed in absence of SET7/SET29-mediated methylation, leading to excessive parasite proliferation and an imbalance in the host cell-parasite relationship.

## Discussion

SET domain proteins modulate several cellular processes through their targets, including histones, transcription factors, proteins involved in cell cycle control, and proteins modulating DNA repair to name a few. Of the 29 SET proteins annotated in *T. brucei*, the consolidated efforts of Sunter *et al.*, Staneva *et al.*, and more recently Billington *al.*, have revealed eight to be nuclear in the blood form (BF) parasites, four of which are nuclear in the procyclic form (PF) parasites also, while six other SET proteins are nuclear only in the PFs (18, 19, 20). None are exclusively nuclear. The large number of non-nuclear SET proteins identified in this organism suggests that several cytosolic proteins may be their targets. LdSET29, like TbSET29, is ubiquitously found within promastigotes (Fig. S3), and thus it may target nuclear and/or cytosolic proteins. In attempting to decipher the physiological role of LdSET29, we found that the parasite’s response to H_2_O_2_ exposure in *in vitro* promastigote cultures was significantly altered in SET29-depleted (*set29*^*−/+*^ and *set29*^*−/−/+*^) parasites. Enhanced survival of the mutants was coupled to reduced ROS levels, and a modest perturbation of the regulation of peroxidase activity (Figure 1, Figure 2, Figure 3, Figure 4). Furthermore, considering that the response of *set29* mutant parasites to an oxidative environment was very similar to that of *set7* mutant parasites, including the muted activation of ROS in response to H_2_O_2_ and loss of regulation of peroxidase activity, when we investigated genetic and physical interactions between LdSET7 and LdSET29 we found the two proteins interacted *in vitro* and *in vivo* (Fig. 6).

While LdSET7 is not essential to the cell under normal growth conditions (22), LdSET29 may be (this study). RNA-seq analysis revealed that LdSET7 and LdSET29 have divergent gene regulatory functions (Figs. 7 and S9). Neither protein appeared to regulate global transcriptional events, although LdSET7 modulated levels of transcripts of ∼500 genes independent of gene dosage (Figs. 7, 8, and S9). While both *set7* and *set29* mutant parasites showed upregulation of peroxidase activity in *in vitro* promastigote cultures ((22), this study), the mechanisms through which they exerted this effect differed in part. *set7* mutants displayed upregulation of *txnPx* transcripts as well as transcripts of other peroxidases ((22), Table S1). *set29* mutants, on the other hand, did not display any changes in levels of peroxidase transcripts, and thus seem to be regulating peroxidase activity either at the level of translational control or at the level of activity *per se*. TxnPx protein levels have been reported to be elevated in response to H_2_O_2_ exposure in *Leishmania* promastigotes (32), and as wild-type cells do not display upregulation of *txnPx* transcripts under these conditions (our findings from RNA-seq), the upregulation of TxnPx protein expression is due to a second tier of control. Thus far we have no evidence of LdSET7/SET29 regulating this second tier of control. While in case of both *set7* and *set29* mutants, ectopic expression of the SET7 and SET29 proteins in the respective mutants led to partial rescues of the mutants’ phenotypes, as both mutants displayed somy changes of certain chromosomes indirect effects due to alterations in gene dosage cannot be ruled out.

In working toward identifying possible target substrates through which SET7 and SET29 may mediate their effects, we have used a combination of immunoprecipitations (of proteins marked with methyllysine modification marks) and mass spectrometry. Based on the results obtained (Fig. 10*B*), we propose that SET7/SET29 may act concertedly on Hsp60 and T-complex protein 1, modulating their expression/activity. A large number of post-translational modifications have been identified in human Hsp60, including phosphorylation, acetylation, ubiquitination and methylation (42). While three sites of lysine methylation have been identified, their functional relevance remains uncovered. These sites are not conserved in LdHsp60. Considering the possibility of multiple lysines being methylated in LdHsp60, at least one of them may be targeted by SET7/SET29. *L. donovani* Hsp60 expression is upregulated in response to H_2_O_2_-induced oxidizing environment in virulent but not avirulent promastigotes, implicating a role for Hsp60 in helping the parasite overcome the effects of oxidative stress and ROS (40). *L. donovani* TCP1 has also been implicated in the modulation of the parasite’s response to miltefosine-induced oxidative stress, working by the alleviation of ROS levels through increased trypanothione peroxidase protein expression (41).

We hypothesize that by targeting Hsp60 and TCP1, LdSET7/SET29 may modulate their expression levels and/or activity, supporting a measured parasite response to oxidative stress that helps hold the internal ROS equilibrium, which would allow the parasite to proliferate in a restrained manner while maintaining ROS at suitable levels for normal intracellular signaling. The life cycle of promastigotes *in vivo* involves a series of events that commences with the differentiation of the aflagellate amastigotes ingested with bloodmeal, into flagellate promastigotes that spend a major part of their life in the sandfly’s mid-gut, before eventually migrating to the salivary glands. While there is no direct evidence demonstrating an oxidative milieu in the insect mid-gut, nor is there evidence of induction of oxidative stress/ROS production in the insect midgut in response to *Leishmania* infection, it has been proposed that the biochemical and energy changes accompanying the differentiation of amastigotes to promastigotes may be coupled to ROS production within the parasite and a corresponding induction of oxidative stress (43, 44). In this scenario, LdSET29/LdSET7 could act to moderate the parasite response to this stress.

Macrophages are central to parasite elimination as well as to parasite survival. The oxidative burst produced within macrophages upon *Leishmania* infection has a two-pronged effect: on the one hand, the ROS attack the parasite and subdue the infection, while on the other hand, the oxidative stress thus induced activates the host anti-oxidant response to protect it from the damaging oxidative burst. The final outcome of whether infection wins over the host defense mechanisms or *vice versa* depends on a number of factors including, most importantly, the host's immune response. The *set29* and *set7* mutant parasites survived much more proficiently than wild-type parasites in macrophages (Fig. 9, *A* and *C*, (22)), similarly dampening the elicited ROS responses in the macrophages upon infection, as compared to wild-type parasites (Fig. 9*C*) -supporting the possibility of a coactive role for LdSET29 and LdSET7 in amastigotes also. Collectively, our results indicate that LdSET29 and LdSET7 proteins play a role in tuning the balance between the parasite and the host cell in this complex relationship.

The findings from this study indicate that LdSET29 and LdSET7 act collaboratively in moderating the parasite’s response to an oxidative environment, and suggest that while each has a distinctive role (only SET7 modulates transcript levels of a number of genes), they may also work concertedly in the proteins they target (Hsp60 and TCP1). LdSET7 and LdSET29 could be working in part as a heteromer. The mammalian SET domain proteins G9a and GLP have been isolated as G9a-GLP heteromers from mouse cells. While the G9a-GLP heteromer mediates H3K9 dimethylation and the corresponding downstream effects *in vivo* (45), G9a and GLP also exhibit independent functions, with each of the two proteins having independent functions in muscle development and terminal differentiation (46), and only GLP can mediate H3K23 trimethylation (47). The data presented in this paper are among the first obtained towards analyzing the roles of the many SET proteins in *Leishmania* species. Only some of the SET proteins are nuclear, yet a large number of methylations have been identified in deposited histones in trypanosomatids (48, 49). This raises the possibility of SET proteins targeting multiple histone residues. The large repertoire of SET proteins also suggests the possibility of functional redundancy. Further studies are expected to open up new avenues in epigenetic research in these unusual organisms.

## Experimental procedures

### *Leishmania* cultures and treatments

*L. donovani* 1S (Ld1S) promastigotes were maintained at 26 °C in M199 (Lonza, Switzerland and Sigma Aldrich) to which fetal bovine serum (10%; Invitrogen), adenine, and hemin (Sigma Aldrich) were added (50). Procyclics and metacyclics were separated as described (51). Whole-cell lysates were isolated using the M-PER reagent (Thermo Fisher Scientific). To study the growth patterns of promastigotes as earlier (52), cultures were initiated at 1 × 10^6^ cells/ml from stationary phase cultures. Hydroxyurea-based synchronization of promastigotes followed by flow cytometry analyses were performed as described (26). Promastigotes were transfected and clonal lines selected for as earlier (52). The effect of UV irradiation was studied as described earlier (52). The effect of hydroxyurea-induced chronic stress was studied by initiating cultures at 1 × 10^6^ cells/ml from stationary phase cultures and dividing the culture into four parts 48 h later, adding 0.25, 0.5 and 1 mM hydroxyurea (HU) to three parts and continuing one part without HU (control). Cells were counted every 24 h thereafter. The effect of hydrogen peroxide on growth was examined by initiating promastigote cultures at 1 × 10^6^ cells/ml from stationary phase cultures and dividing up the culture 48 h later when cells were at a density of ∼7 to 9 × 10^6^ cells/ml. While one-third to one-fifth of the culture was continued without any treatments as control, the remaining parts were treated with 100 to 400 μM hydrogen peroxide for 4 to 8 h before refeeding the cells with fresh H_2_O_2_-free medium and continuing incubation at 26 °C. Cells were counted every 24 h thereafter.

### Cloning of *set29* gene

The *set29* gene was amplified off Ld1S genomic DNA using end primers SET29-F (5′- GAGAATTCACCATGGCCACCATGTCACCAGTGCTG-3′) and SET29-R (5′-AGCTC GAGATCCATGGTTCCTGTCTCGTTTTTCCA-3′), and the amplicon cloned into the SmaI site of pUC19 for DNA sequencing. The *set29* gene was resected from the pUC/SET29 clone using EcoRI-XhoI enzymes and cloned into the corresponding sites in pASK-IBA43plus (IBA Life Sciences), generating plasmid pASK-SET29 for LdSET29 expression in *E. coli*. For expression in *Leishmania* cells *set29* was amplified using primers SET29-F and SET29-HA-R (5′-TACCATGGTTAAGCGTAATCTGGAACATCGTATGGGTATCCTGTCTCGTTTTTCCAA-3′), and the amplicon cloned into the NcoI site of the pLEXSY_I-neo3 vector (Jena Bioscience) to create plasmid pLEXSY/SET29-HA.

### Expression of LdSET29 in *E. coli* and *Leishmania* cells

For expression in *E. coli* the plasmid pASK-SET29 was transformed into BL21 CodonPlus cells, transformant cells grown to mid-log phase, and expression of recombinant LdSET29 induced using anhydrotetracycline (200 ng/ml) before continuing incubation at 16 °C for 18 h. For expression in *Leishmania* the plasmid pLEXSY/SET29-HA was transfected into logarithmically growing *Leishmania* promastigotes, and clonals selected for on M199 semi-solid medium carrying G418 (50 μg/ml). Clonals were expanded step-wise in M199 carrying G418 (100 μg/ml) and analyzed for SET29-HA expression by western blotting of whole cell lysates isolated from transfectant cells using anti-HA antibodies (Cell Signaling Technology; Catalog no: C29F4; 1:2500 dil).

### Raising antibodies to recombinant LdSET29

Recombinant LdSET29 was purified from whole cell lysates isolated from transformant *E. coli* cells expressing the protein, by lysing the cells using sonication, clarifying the lysate by high-speed centrifugation, and subjecting the lysate to successive affinity-based chromatography through cobalt metal affinity beads and Strep-Tactin II matrix. The purified protein was dialyzed against PBS, and each of the three mice was injected subcutaneously with 50 μg protein in Freund’s complete adjuvant. After two booster shots in Freund’s incomplete adjuvant, test bleeds were drawn to check the production and sensitivity of the antibodies. The antibodies were found to be of very weak titer and further boosters were administered to improve titer. The mice received a total of five boosters 10 days apart, before drawing antiserum for use. Protocols were approved by the Institutional Animal Ethics Committee of the National Institute of Immunology, New Delhi (IAEC #462/18) and executed according to the guidelines of the Committee for Control and Supervision of Experiments on Animals (CCSEA), Govt of India.

### Immunofluorescence analysis

Immunofluorescence analysis was carried out as described earlier (53). Briefly, 5–7 × 10^6^ logarithmically growing promastigotes were collected by centrifugation, washed with 1XPBS, fixed in 2% paraformaldehyde, and cells spread on poly-lysine coated coverslips before permeabilization with 0.1% TritonX-100. The permeabilized cells were blocked with 5% chicken serum, incubated with anti-HA antibody (1:100 dil; Cell Signaling Technology; Catalog no: C29F4), washed, incubated with Texas Red-labelled anti-rabbit secondary antibody (1:100 dil; Jackson ImmunoResearch Laboratories), washed, and mounted in DAPI-containing anti-fade medium (Vectashield from Vector laboratories). Cells were observed by excitation at 594 nm and monitoring emission at 615 nm, using a 100× (in oil) objective under a Leica TCS SP8 confocal microscope, and Z-stack images were collected and analyzed with the LAS X software.

### Creation of knockout and rescue lines

To make genomic knockouts of *set29*, donor plasmids were made by amplifying the ∼800 bp sequence stretches immediately upstream and downstream of the *set29* gene and cloning these 5′flank and 3′flank sequences upstream and downstream of suitable drug resistance cassettes. The upstream flank was amplified using primers SET29-5′Fl-F (5′-TAGCGGCCGCGATATCGCTTCTCCACTAAAG-3′) and SET29-5′Fl-R (5′-TAGCGGCC GCTGTAACAACCGAGAAGAGCC-3′), and cloned into the NotI site of pLEXSY-egfp-neo3 (Jena Biosciences, Germany) and pLEXSY-eGFP-hyg (54, 55). The downstream flank was amplified using primers SET29-3′Fl-F (5′-TAACTAGTTGATGTCCCCGTTTAC TCCAAAC-3′) and SET29-3′Fl-R (5′-TAACTAGTGATATCCAGGCACATCGCGTAGT-3′), and cloned into the SpeI site of the pLEXSY clones carrying the 5′flank sequences, that were generated above. The donor plasmids so generated were named pSET29/KO-hyg and pSET29/KO-neo respectively.

To knock out the first allele, the donor cassettes were released from pSET29/KO-hyg and pSET29/KO-neo by EcoRV digestion, and transfected separately into *L. donovani* promastigotes, and clonals selected for by plating on semi-solid M199 medium carrying hygromycin (16 μg/ml) and G418 (50 μg/ml), respectively. Colonies that appeared 12 to 14 days later were expanded in liquid culture under selection pressure (hygromycin: 32 μg/ml and G418: 100 μg/ml) and screened for authentic homologous recombination at both ends, by isolating their genomic DNA and carrying out PCRs across the deletion junctions. The *set29* heterozygous knockout was named *set29*^*−/+*^. To knock out the second allele, the donor cassette was similarly released from pSET29/KO-neo and transfected into *set29*^*−/+*^ cells that carried the hygromycin resistance cassette. Clonals were selected for on medium carrying both drugs, hygromycin (16 μg/ml) and G418 (50 μg/ml). Colonies were expanded and screened for homologous recombination at both ends, by PCRs across the deletion junctions using genomic DNA as template. The line so created was named *set29*^−/−/+^.

In absence of the successful creation of a true *set29*-null, the rescue line was made using the *set29*^−/−/+^ cells as the background strain. To express the *set29* gene in *set29*^−/−/+^ cells, the *set29* gene was cloned in fusion with a FLAG tag in pXG-FLAG(bleo) (52) using primers SET29-FLAG-F (5′- TAGATATCATGTCACCAGTGCT-3′) and SET29-FLAG-R (5′-TAGATATC TGTCTCGTTTTTCCA-3′), and the plasmid pXG-SET29-FLAG(bleo) so created was transfected into *set29*^*−/−/+*^ cells. Clonals were selected for by plating on medium carrying hygromycin (16 μg/ml), G418 (50 μg/ml), phleomycin (2.5 μg/ml), and colonies were expanded (phleomycin in liquid culture: 6 μg/ml; hygromycin and G418 as above) and screened for expression of SET29-FLAG using Western blot analysis of whole cell lysates isolated from them. One clone expressing SET29-FLAG robustly was used for further studies, and the line named *set29*^*−/−/+*^::SET29^+^.

### Promastigote TUNEL assay

H_2_O_2_-induced DNA strand breaks were labeled using the DeadEnd Fluorometric TUNEL System (Promega) as per the manufacturer’s instructions and as described earlier (22). Briefly, wild type and *set29* mutant parasite cells (treated/untreated) were collected by centrifugation, washed with 1XPBS, fixed with 2% paraformaldehyde, spread on poly-lysine coated coverslips, permeabilized using 1× PBS-0.2% Triton X-100, incubated with the tailing reaction mix for an hour, washed, and mounted in DAPI-containing anti-fade solution. Cells were observed using a 100× (in oil) objective under a Leica TCS SP8 confocal microscope. Z-stack images were captured and analyzed with the LAS X software.

### Measurement of ROS in promastigotes

Intracellular ROS levels were measured using the dichlorodihydrofluorescein diacetate (DCFDA) assay as earlier (22). Briefly, parasite cultures were initiated from stationary phase cultures as above and were treated for 45 min with H_2_O_2_ (100 μM) 48 h later (when cell density was ∼7–9 × 10^6^ cells/ml), before refeeding the cells with H_2_O_2_-free medium and continuing incubation at 26 °C. Aliquots of cells were drawn from the cultures at various times thereafter, and the DCFDA assay performed as detailed (22).

### Measurement of peroxidase activity in promastigotes

Peroxidase activity was measured using the Amplex Red assay as described earlier (22). Briefly, parasite cultures were initiated from stationary phase cultures as above and were treated with H_2_O_2_ (100 μM) 48 h later (when cells were at a density of ∼ 7–9 × 10^6^ cells/ml) for 5 h, before refeeding the cells with H_2_O_2_-free medium and continuing incubation at 26 °C. Aliquots of cells were drawn from the cultures at various times thereafter, and the Amplex Red assay performed as described (22).

### Pulldown assays

The *set7* and *set29* genes were cloned into the pET/Duet vector. The *set7* gene was cloned into the EcoRI-SalI sites of the vector, creating plasmid pET-Duet/SET7. The *set29* gene was cloned into the BglII site of the pET/Duet vector as well as the pET-Duet/SET7 plasmid, creating plasmids pET-Duet/SET29 and pET-Duet/SET7-SET29. The plasmids were transformed into *E. coli* BL21 Codon Plus cells and expression induced in transformant cells (100 ml logarithmically growing cultures) with IPTG (1 mM), followed by incubation at 16 °C for 18 to 20 h. Whole cell lysates were isolated from these cells (in 100 mM Tris.Cl (pH 8), 150 mM NaCl, 10% glycerol), and incubated with cobalt-agarose beads (200 μl TALON metal affinity resin) to allow for protein binding. The unbound proteins were washed off with high salt buffer (100 mM Tris.Cl (pH 8), 1M NaCl, 10% glycerol) and the bound fraction eluted in 200 μl of 100 mM Tris.Cl (pH 8), 300 mM NaCl, 250 mM imidazole and 10% glycerol. 1/20 of the eluate fractions were analyzed by SDS-PAGE followed by Coomassie staining. 1/200 of the eluate fractions were analyzed by SDS-PAGE followed by western blotting with anti-SET7 and anti-SET29 antibodies (each at 1:2500 dil).

### Creation of *Leishmania* bimolecular fluorescence vectors and cloning of *set* genes in them

The vector pLEXSY/YFPn was created by amplifying the N-terminal fragment of YFP off the vector pDH51-GW-YFPn (a kind gift from Prof Sanjay Kapoor, University of Delhi South Campus) using primers YFPn-F (5′-TATCCATGGTTCATATGGTGAGCAAGGGCGAG-3′) and YFPn-R (5′-TAGCGGCCGCTTAGGCGGTGATATAGACGT-3′) and cloning the fragment into the NcoI-NotI sites of pLEXSY-I-neo3 (Fig. S7*A*). The vector pLeish/YFPn/YFPc was created using a two-step approach. The C-terminal fragment of YFP was first amplified off the vector pDH51-GW-YFPc (also a gift from Prof Sanjay Kapoor) using primers YFPc-F (5′-TAGATATCGACAAGCAGAAGAACGGCATC-3′) and YFPc-R (5′-TAGATATCACTAGTTTACTTGTACAGCTCGTC-3′) and the fragment was cloned into the EcoRV-NruI sites of vector pXG-/GFP+ (a gift from Prof. Beverley; (56)), to generate plasmid pXG/YFPc (Fig. S7*B*). This was followed by resecting the fragment along with ∼1.3 kb upstream sequence using SpeI digestion (an SpeI site was incorporated into the YFPc-R primer), and the released fragment was cloned into the SpeI site of pLEXSY/YFPn to create vector pLeish/YFPn/YFPc (Fig. S7*C*).

The *set7* gene was amplified using primers SET7-YFPc-F (5′-TAGGCCGTGCCGGCCATGCCCATCAGCCAG-3′) and SET7-YFPc-R (5′-TAGGCCGG CACGGCCAGGAAGAAGAGGCTTC-3′) and cloned into the SfiI site of pLeish/YFPn/YFPc to create plasmid pLeish/YFPn/SET7-YFPc. The *set29* gene was amplified using primers SET29-YFPn-F (5′-TAAGATCTAATGTCACCAGTGCT-3′) and SET29-YFPn-R (5′-TAAGATCTTGTCTCGTTTTTCCA-3′) and cloned into the BglII sites of pLeish/YFPn/YFPc and pLeish/YFPn/SET7-YFPc, to create plasmids pLeish/SET29-YFPn/YFPc and pLeish/SET29-YFPn/SET7-YFPc respectively. The *set26* gene was amplified and cloned into the BglII site of pLeish/YFPn/YFPc to create plasmid pLeish/SET26-YFPn/YFPc, into which the *set7* gene was cloned at the SfiI site to create plasmid pLeish/SET26-YFPn/SET7-YFPc. The *set21* gene was amplified and cloned into the SfiI site of pLeish/YFPn/YFPc to create plasmid pLeish/YFPn/SET21-YFPc, into which the *set29* gene was cloned at the BglII site to create plasmid pLeish/SET29-YFPn/SET21-YFPc.

### Bimolecular fluorescence complementation

The various plasmid constructs (described above) were transfected into *L. donovani* promastigotes, and transfectants were analyzed by fluorescence microscopy. For this, 5 to 7 × 10^6^ logarithmically growing transfectant cells were fixed with 2% paraformaldehyde, and cells spread on poly-lysine coated coverslips before mounting in DAPI-containing anti-fade solution. Bimolecular fluorescence was analyzed by excitation at 513 nm and monitoring emission at 527 nm. Cells were observed with a 100× (in oil) objective using a Leica TCS SP8 confocal microscope. Images were captured by Z-stacking and analyzed using Leica LAS X software.

### Isolation of RNA and analysis by RNA-seq

Two sets of experiments were carried out for RNA-seq analysis of *set7* and *set29* mutant parasites. In the first set of experiments, wild type and *set7*^−/−^ parasites were exposed to 100 μM H_2_O_2_ for 5h (following the same treatment protocols as above) and allowed to recover for 3 h thereafter in H_2_O_2_-free medium before isolation of RNA. In the second set of experiments, wild-type and *set29*^−/−/+^ parasites were similarly exposed to 100 μM H_2_O_2_ for 5 h and allowed to recover for 3 h before isolation of RNA. Two biologicals were analyzed in each set of experiments. RNA was isolated using the PureLink RNA isolation kit (Invitrogen). On-column DNase I treatment (PureLink DNase Cat. no. 12185-010) was performed during the isolation to eliminate genomic DNA contamination in the isolated RNA, as per manufacturer’s instructions. RNA integrity was analyzed using the 4200 TapeStation system (Agilent Technologies). Sequencing was carried out at the Core Sequencing Facility, Centre for Cellular and Molecular Biology, India, using the Illumina NovaSeq 6000 sequencing system. RNA-seq library for the first set of experiments was prepared using the TruSeq RNA Library Prep Kit v2. RNA-seq library for the second set of experiments was prepared using the Illumina Stranded mRNA Prep kit.

To carry out data analysis, the reference genome sequence and gene annotation information for *L. donovani* LdBPK282A1 were downloaded from the TriTrypDB portal (57). Raw reads were pre-processed by trimming the adapter sequences with TrimGalore (v0.6.10) (https://github.com/FelixKrueger/TrimGalore), using the arguments: “--quality 20 --paired --length 25”. The paired end reads were then aligned to the indexed genome using STAR (v2.7.10b) (58), and the reads mapping to genomic features were counted in the alignment step using the “–quantMode GeneCounts” option. To identify the differentially expressed genes (DEGs), the per-sample raw count files generated by STAR were concatenated into a master count table, with rows and columns representing genes and samples respectively. An expression cut-off of 200 was empirically chosen based on the distribution of total fragment counts per gene across all samples: genes with total count less than 200 across all samples of an experimental set were filtered out. The differentially expressed genes within the sample sets were identified using the DESeq2 (59) package from Bioconductor (60). All genes with an absolute log_2_ fold change ≥0.6 and ≤−0.7 and an adjusted *p*-value less than 0.05 were considered for interpretation. All heatmaps were made with pheatmap (https://github.com/raivokolde/pheatmap) and all other data were plotted using ggplot2. In preparing heatmaps, the DESeq2-normalized read counts for a particular gene across all samples were subjected to a Z-score normalization. Z-scores for every gene across all four samples were calculated by subtracting the mean value of the gene (from values across all the samples) and dividing the obtained values by standard deviation. Computed Z-scores were used to plot the heat maps. Differentially expressed genes were analysed for Gene Ontology (GO) term enrichment for biological processes using TriTrypDB (v68), against the *L. donovani* BPK28A21 GO database. In carrying out the data analysis, a *p* value cutoff of <0.05 from Fisher’s exact test was used for selection of the enriched GO terms. The data plotted depicts the DEGs with the specific GO term as a percent of the total number of genes with the same GO term in the *L. donovani* BPK28A21 GO database.

### Whole-genome sequencing and analysis

Genomic DNA was isolated from wild type and *set* mutant promastigotes in mid-logarithmic phase of growth, using the DNeasy Blood and Tissue Kit (Qiagen cat no: 69504) as per the manufacturer’s instructions, with the DNA being eluted from the columns using 10 mM Tris.Cl (pH 8.5). Libraries for whole genome sequencing were prepared using the Illumina DNA Prep Kit with 100 ng input genomic DNA, as per the manufacturer’s instructions. The libraries were quantitated to determine DNA concentrations (in ng/μl) using the Qubit dsDNA HS Assay Kit (Thermo Fisher Scientific). Sizes of fragments in the generated libraries were assessed using the Agilent TapeStation System, and the average library fragment size (in bp) was determined. The libraries were suitably diluted to a concentration of 4 nM for paired-end sequencing (2 × 151 bp read length) using the Novaseq 6000 platform.

In analyzing the genome sequence data of each cell type (wild type and three *set* mutants) WGS paired-end reads were pre-processed to remove adaptor sequences, followed by alignment of the reads to the *L. donovani* reference genome LdBPK282A1 obtained from TriTrypDB (www.tritrypdb.org; (35, 57)) using BWA-MEM v0.7.18 (61). Duplicate and low-quality alignments assigned as such by the sequencing platform were excluded, and the remaining reads were sorted by coordinates using SAMtools v1.20 (62). For each genome sequence data set, the read depth per base per chromosome was obtained by running SAMtools depth with the -aa flag.

Somy estimations were carried out as described earlier (63). Briefly, each chromosome was first partitioned into 5 kb bins, and total read depth for each bin was calculated. The median and median absolute deviation of these values were then used to filter out any outlier bins, by retaining only those within ± 1 MAD of the median value. The median chromosomal read depth was then recalculated as the median of the retained bins. Length bias-corrected depth value for each chromosome was determined by dividing the median depth by the median of medians of depths of the 14 nearest chromosomes by size (including itself). Somy (S) was calculated by taking twice the value of this ratio and binning them: monosomy, S ≤ 1.5; disomy, 1.5< S ≤ 2.5; trisomy, 2.5 < S ≤ 3.5; tetrasomy, 3.5 < S ≤ 4.5. To estimate the number of copies of a specific gene, we determined the ratio *‘r’* of the read depth of the gene to the median read depth of that chromosome. Copy number ‘*C*_*N*_’ was estimated using *C*_*N*_ = *r* x *S* where *S* was the somy of that chromosome.

### Macrophage infections

Infections of murine macrophage J774A.1 cells with *Leishmania* parasites were carried out as detailed (54). Briefly, macrophages were plated 24 h before infection in DMEM on poly-lysine coated coverslips in 6-well dishes. For a 5-h infection period, the macrophages were incubated with metacyclic parasites in serum-free medium (host cell: parasite ratio of 1:10). The non-internalized parasites were removed by aspiration and the macrophages refed with complete medium. Infections were analyzed microscopically at various times thereafter. Slides were prepared by fixing the cells in 4% paraformaldehyde before mounting in a DAPI-containing Vectashield mounting medium (Thermo). Intracellular parasites were scored using their nuclei as marker, by confocal microscopy with the Leica TCS SP8 confocal microscope. Z-stack images were captured and analyzed with the LAS X software.

### Intracellular parasite TUNEL assay

Macrophages plated on coverslips were infected with *Leishmania* parasites for 5 h, before refeeding them with complete DMEM. The infected cells were incubated for 12 h from start of infection, fixed in 4% paraformaldehyde for 25 min, and washed with 1× PBS. TUNEL assay was performed on these cells as above, and intracellular fluorescein-labeled parasite nuclei scored by acquiring Z-stack images using the Leica TCS SP8 confocal microscope and analyzing the images with the LAS X software. Total intracellular parasite load was determined by scoring DAPI-stained parasite nuclei, and the percentage of parasite nuclei that were fluorescein-labeled by the TUNEL reaction were calculated. Around 100 to 125 macrophages were analyzed in each sample type. The experiment was performed thrice and average values are shown in the bar graphs, with error bars depicting standard deviation.

### Measurement of ROS in infected macrophages

The DCFDA assay was performed as described (64). Briefly, macrophages were plated in the wells of a 24-well culture dish at a density of 5 × 10^5^ cells per well in 500 μl complete DMEM, and incubated at 37 °C for 24 h in 5% CO_2_ atmosphere, followed by infection with 7.5 × 10^6^ metacyclic parasites (host cell: parasite ratio of 1:15) for 5 h. After removing the non-internalized parasites, the wells were rinsed with 1× PBS and the infected macrophages refed with fresh complete DMEM before continuing incubation at 37 °C for varying times thereafter. To perform the DCFDA assay, the wells carrying the infected macrophages were washed with 1× PBS, and fresh 1× PBS carrying the DCFDA dye (10 μM) added before continuing incubation at 37 °C for 45 min in the dark. The reaction mix was replaced with fresh 1× PBS before analyzing fluorescence at 529 nm (ex: 488 nm).

Reactions carried out without cells were set up at every time-point and these yielded “background” fluorescence values, which were subtracted from values of all reactions with cells. At every time-point reactions were carried out with uninfected macrophages, as well as macrophages infected with wild-type parasites, *set29*^−/−/+^ parasites, and *set7*^−/−^ parasites. The experiment was performed thrice, with two technical replicates in all biologicals, and average values of the three experiments are shown in the bar graphs, which represent fold increase in ROS accumulation upon infection with *Leishmania* parasites (wild type or *set* mutants). Indicated times are from start of infection. Error bars depict standard deviation. Statistical significance was determined with student’s two-tailed *t* test.

### Measurement of peroxidase activity in infected macrophages

Macrophages were plated in 100 μl complete DMEM at a density of 5 × 10^4^ cells per well in a 96-well dish, and incubated at 37 °C for 24 h in 5% CO_2_ atmosphere, followed by infection with 7 × 10^5^ metacyclic parasites (host cell: parasite ratio of 1:15) for 5 h. After removal of non-internalized parasites by aspiration the wells were rinsed with 1× PBS, 100 μl complete DMEM added, and incubation continued for varying times thereafter before performing the peroxidase assay. To perform the peroxidase assay, the cells were washed with 1× PBS and the Amplex Red assay reaction mix (1× PBS, 64 μM digitonin, protease inhibitors, 1 mM H_2_O_2_, 10 μM Amplex Red) added, followed by incubation at 37 °C in the dark for 30 min. Fluorescence was measured by excitation at 535 nm and monitoring emission at 590 nm.

Reactions without cells set up at every time point yielded “background” fluorescence values, which were deducted from the values of all reactions with cells. Reactions were carried out with uninfected macrophages, and macrophages infected with wild-type parasites, *set29*^−/−/+^ parasites, and *set7*^−/−^ parasites, at every time-point. The experiment was performed thrice, with two technical replicates in all biologicals, and average values of the three experiments are shown in the bar graphs, which represent fold increase in peroxidase activity upon infection with *Leishmania* parasites (wild type or *set* mutants). Indicated times are from start of infection. Error bars depict standard deviation. Statistical significance was determined with student’s two-tailed *t* test.

### Immunoprecipitations and proteomics analysis

Lysates were isolated from 1 × 10^9^ parasites in the mid-logarithmic phase of growth, using the M-PER reagent (to which protease inhibitors were added). The lysates were treated with DNase I prior to clarification by high-speed centrifugation (14,000*g* for 40 min at 4 °C), and these clarified lysates were used in immunoprecipitations. Immunoprecipitations (IPs) were carried out using anti-methyllysine antibodies (Invitrogen, Cat. no: PA5-77770). For each IP reaction, 5 μl antibodies were incubated with 50 μl of a 1:1 (v/v) slurry mix of Protein A-Sepharose and Sepharose CL-6B beads, for 1 hour on ice with intermittent mixing. Unbound antibodies were washed off, and the bead-bound antibodies were added to lysates harboring ∼2 mg total protein, followed by incubation at 4 °C for 5 h with inversion-mixing. After washing off unbound proteins (6 washes with 1XPBS-0.1% TritonX-100, each wash being carried out by inversion-mixing for 3 min at 4 °C followed by the collection of washed beads by low-speed centrifugation at 4 °C), the bead-bound IP mix was incubated with 70 μl SDS-PAGE sample loading buffer at 95 °C for 10 min with intermittent mixing. This was followed by high-speed centrifugation to separate the beads from the released proteins. The fraction of released proteins was loaded on SDS-PAGE (10%), and the gel piece carrying the loaded protein fraction was excised from the resolving gel as soon as the stacked proteins entered the resolving gel, using a pre-stained molecular weight marker as a guide for the extent of electrophoresis. The protein fraction in the excised gel piece was subjected to in-gel reduction using DTT (10 mM) in ammonium bicarbonate (25 mM), followed by in-gel alkylation using iodoacetamide (50 mM) in ammonium bicarbonate (25 mM). The gel piece was dehydrated in acetonitrile (100%), and this was followed by in-gel tryptic digestion with 1.5 to 2 μg trypsin (Pierce Trypsin Protease MS grade Cat no. 90059, Thermo Fisher) in 300 μl ammonium bicarbonate (25 mM) at 37 °C for 16 h. The peptides were extracted from the gel pieces using acetonitrile solution (80% acetonitrile, 0.1% formic acid) and dried using a vacuum concentrator, followed by sample clean-up using Pierce C18 spin columns (Thermo Scientific). The eluted peptides were dried by vacuum concentration. The samples were resuspended in an appropriate volume of 2% acetonitrile-0.1% formic acid and subjected to LC-MS analysis using Thermo Fisher Scientific’s Orbitrap mass analyzer system.

Samples of Biologicals 1, 2, and 3 were analyzed using the Thermo Scientific Easy-nLC 1200 system coupled to the Q Exactive Plus Orbitrap system (at the Central Instrumentation Facility, University of Delhi South Campus). Peptides were resolved on Thermo Scientific EASYSpray PepMap RSLC C18 column (Thermo Scientific) using a 5%-10%-45%-90% Solvent B gradient (Solvent A: 2% acetonitrile-0.1% formic acid; Solvent B: 80% acetonitrile-0.1% formic acid) with a flow rate of 300 nl/min. MS1 scans ranged over 350 to 2000 m/z, with AGC target 3.0e6, maximum ion transfer time 50 ms, resolution 70,000, and filter for minimum charge state two. MS2 scans ranged over 200 to 2000 m/z, with AGC target 1.0e5, maximum ion transfer time 120 ms. Analysis was performed using the Proteome Discoverer 2.4 software (label-free quantification), panning the data against the *L. donovani* proteome library available in TriTrypDB (TriTrpDB-68_LdonovaniBPK282A1_AnnotatedProteins.fasta). Peak lists were generated using the Sequest-HT algorithm. Parameters that were considered during analysis were as follows: precursor ion mass tolerance (MS), 10 ppm; fragment ion mass tolerance (MS/MS), 0.02 Da; up to two missed cleavages allowed; peptides shorter than six amino acids and longer than 144 amino acids excluded; cysteine carbamidomethylations as fixed modifications; False Discovery Rate (FDR) of 1% applied. The fold enrichment (log_2_) values were determined using the Proteome Discoverer application, which calculated the quantification abundances and ratios from the raw quantification values using the Pairwise Ratio approach.

Biological four samples were analyzed using the Thermo Scientific Easy-nLC 1200 system (at the Core Proteomics Facility, Centre for Cellular and Molecular Biology). Peptides were resolved on Thermo Scientific PepMap RSLC C18 column using a 0%-25%-45%-95% Solvent B gradient (Solvent A: 5% acetonitrile -0.2% formic acid; Solvent B: 90% acetonitrile -0.2% formic acid), coupled to the Orbitrap Exploris 240 system, in DDA mode. MS1 scans ranged over 400 to 1650 m/z, with AGC target “standard”, maximum ion injection time “auto”, resolution 60,000, and filter for minimum charge state two. Analysis was performed using the Proteome Discoverer 2.2.0.388 software (label-free quantification), panning the data against the *L. donovani* proteome library available in the TriTrypDB (TriTrpDB-68_LdonovaniBPK282A1_AnnotatedProteins.fasta). Peak lists were generated using the Sequest-HT algorithm as above. Parameters that were considered during analysis were as above, except that precursor ion mass tolerance (MS) of 5 ppm and fragment ion mass tolerance (MS/MS) of 0.06 Da was applied.

## Data availability

All relevant data are within the manuscript and its Supporting Information files. Ld1S *set29* sequence has been deposited in GenBank, Accession no: OR479703. All RNA-seq data have been deposited at the NCBI Gene Expression Omnibus (GEO) database: for the first experimental set with wild type and *set7* mutant: GSE267955 (https://www.ncbi.nlm.nih.gov/geo/query/acc.cgi?acc=GSE267955), and for the second experimental set with wild type and *set29* mutant: GSE267957 (https://www.ncbi.nlm.nih.gov/geo/query/acc.cgi?acc=GSE267957). All genome sequence data have been deposited at the European Nucleotide Archive (ENA) database: PRJEB79494. All proteomics data have been deposited at the EMBL Proteomics Identifications Database (PRIDE): PXD056970, PXD057513, PXD057514, PXD057511.

## Supporting information

This article contains supporting information.

## Conflict of interest

The authors declare that they have no conflicts of interest with the contents of this article.
