# Supplementary material for: The SET29 and SET7 proteins of Leishmania donovani exercise non-redundant convergent as well as collaborative functions in moderating the parasite’s response to oxidative stress
Source: J Biol Chem. 2025 Jan 20;301(3):108208. doi: 10.1016/j.jbc.2025.108208 (PMC11871502; doi:10.1016/j.jbc.2025.108208)
Supplement: Supporting Information Figures [file mmc1.pdf]

The SET29 and SET7 proteins of *Leishmania donovani* exercise non-redundant convergent as well as collaborative functions in moderating the parasite's response to oxidative stress

Varshni Sharma[1#], Jyoti Pal[1#], Vishal Dashora[1], Somdeb Chattopadhyay[2], Yogita Kapoor[3,4], Biplab Singha[2,3,5], G. Aneeshkumar Arimbasseri[2] and Swati Saha[1*]

[1]Department of Microbiology,
University of Delhi South Campus,
New Delhi, India

[2]National Institute of Immunology,
New Delhi, India.

[3]Centre for Cellular and Molecular Biology,
Hyderabad, India.

[4] Academy of Scientific and Innovative Research (AcSIR),
Ghaziabad, India.

[5] Current affiliation:
Department of Medicine and Biological Sciences,
Cedars-Sinai Samuel Oschin Comprehensive Cancer Institute,
Los Angeles, USA

# *These two authors contributed equally to the work*

****To whom correspondence may be addressed***

Running title: Role of *Leishmania donovani* SET29 and SET7 proteins

Keywords: *Leishmania donovani,* trypanosome, SET domain, SET proteins, SET29, SET7, oxidative stress, protozoan parasite, BiFC in *Leishmania*

Figure S1

A.

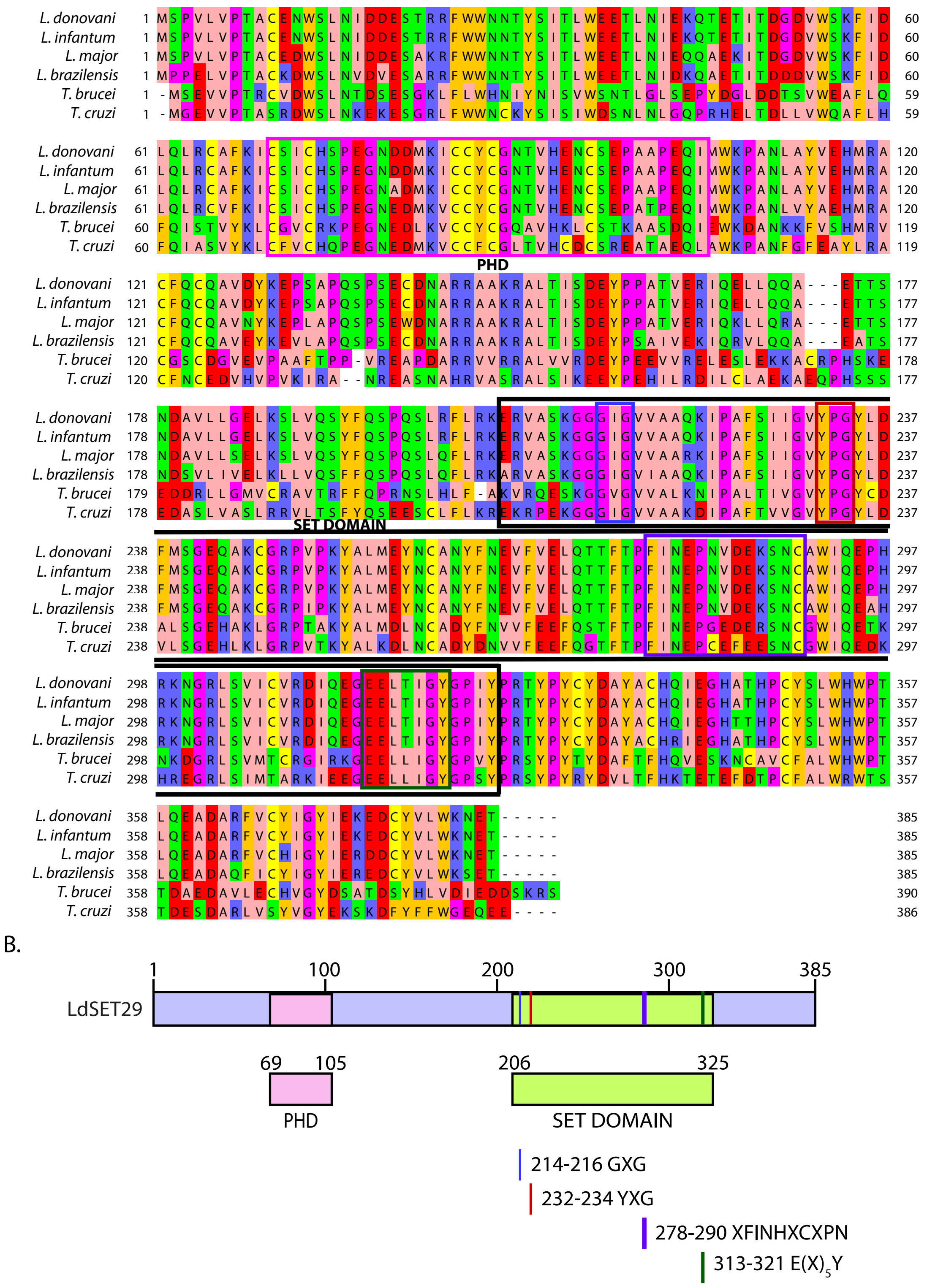

Figure S1: A. Comparison of LdSET29 amino acid sequence with amino acid sequences of other trypanosomatid SET29 proteins. Analysis carried out with Clustal Omega [24], viewed using Jalview multiple alignment editor. PHD and SET domains are demarcated with magenta and black boxes respectively. Colors indicate the physico-chemical properties of the amino acids. Pink- hydrophobic/aliphatic; red- acidic; purple- glycine / proline; yellow- cysteine; orange/ochre- aromatic; dark blue- basic; green- hydrophilic. **B.** Domain analysis of LdSET29 protein. The ~120 amino acid long SET domain carries the four motifs that are characteristically involved in AdoMet binding and catalysis (indicated in figure).

Figure S2

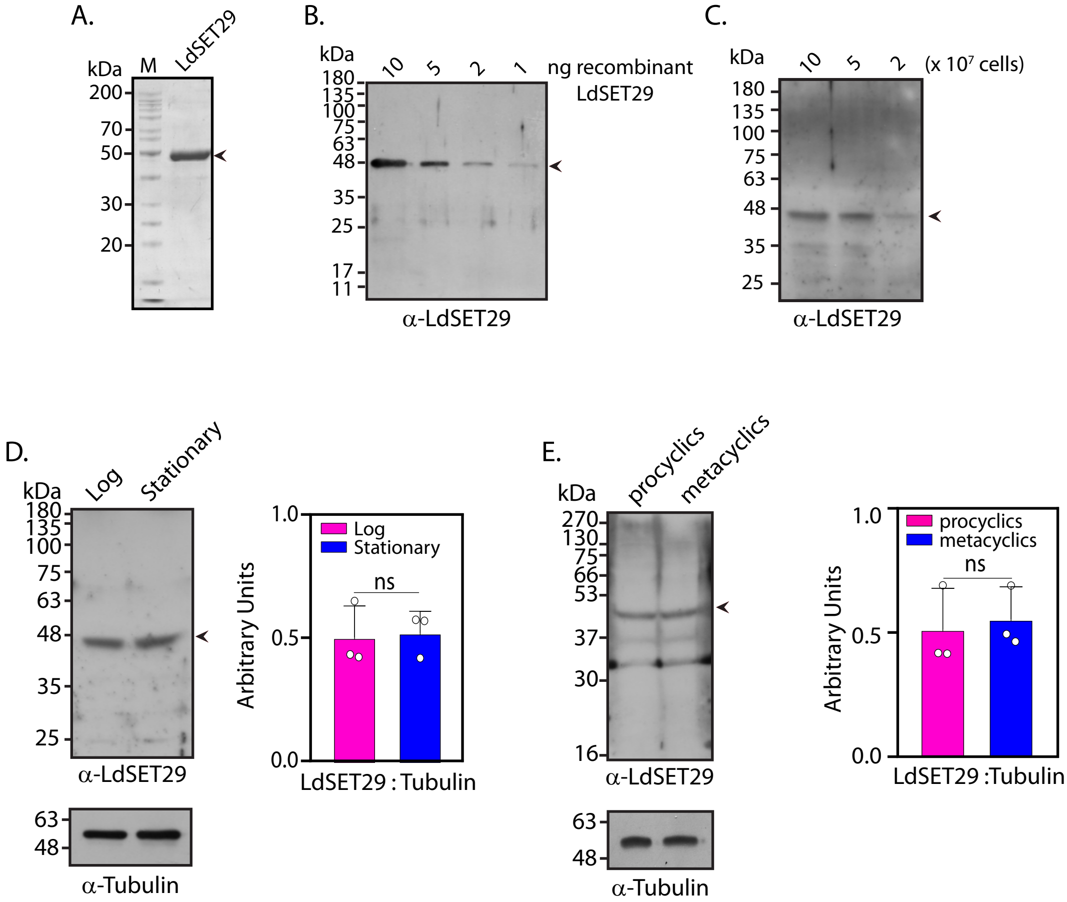

Figure S2: A. Coomassie stain of SDS-PAGE analysis of purified recombinant LdSET29 protein. **B.** Analysis of sensitivity of anti-LdSET29 antibodies (1:1000 dil), tested against varying amounts of recombinant LdSET29 in western blots. **C.** Analysis of specificity of anti-LdSET29 antibodies (1:1000 dil), tested against varying amounts of whole cell lysates isolated from logarithmically growing *Leishmania* promastigotes. **D.** Western blot analysis of expression of LdSET29 in $1x10^8$ logarithmically growing and stationary phase *Leishmania* promastigotes using anti-LdSET29 antibodies (1:1000 dil). Tubulin served as loading control. **E.** Western blot analysis of expression of LdSET29 in $1x10^8$ *Leishmania* procyclics and metacyclics using anti-LdSET29 antibodies (1:1000 dil). Tubulin served as loading control. The experiments in D. and E. were done thrice. ImageJ was used for quantitation and bar graphs represent average values, with open circles on bar graphs marking values of individual experiments. Error bars depict standard deviation. Statistical significance was determined using the two-tailed unpaired student's t-test. ns: not significant.

Figure S3

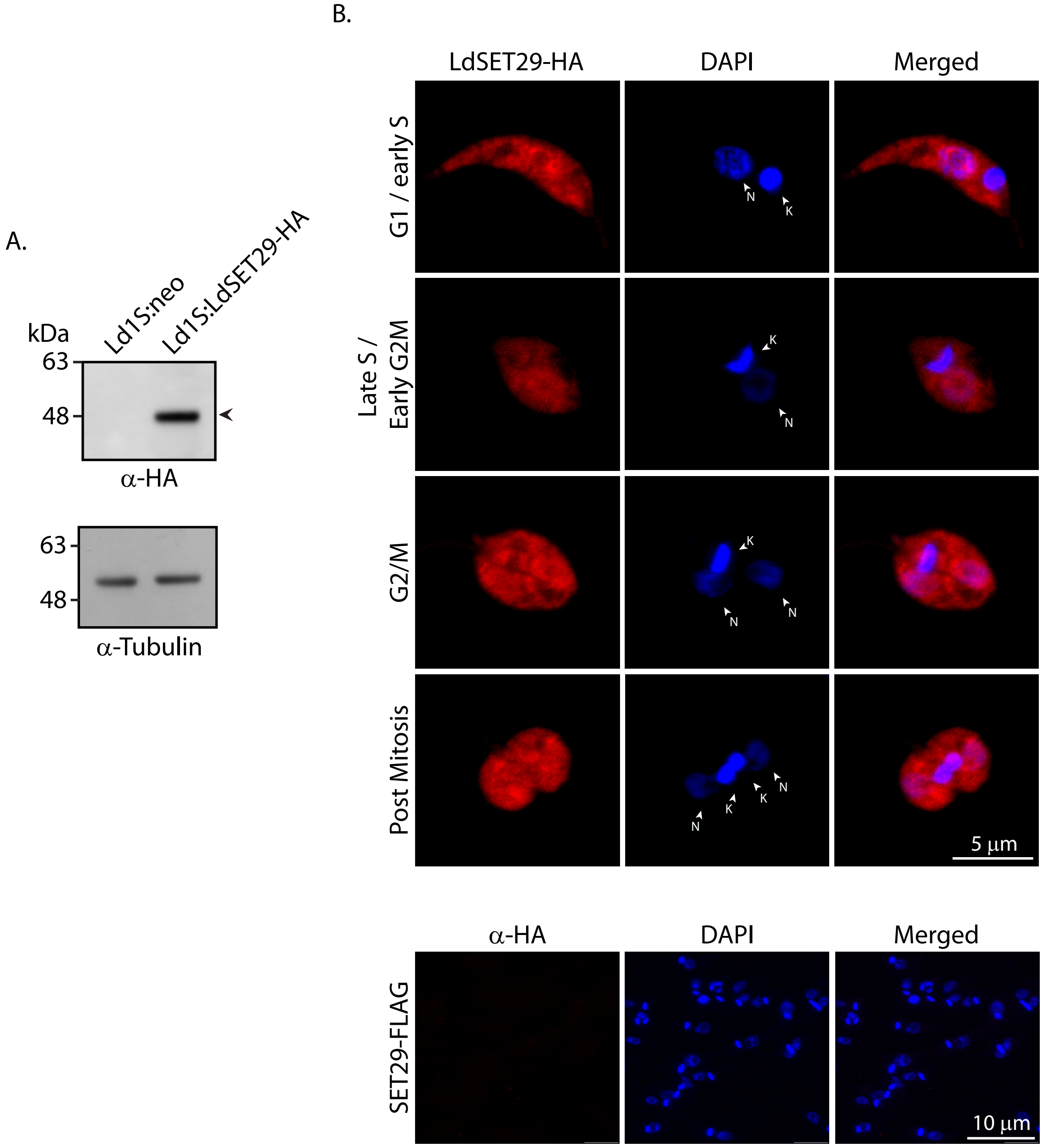

Figure S3: **Determination of the subcellular localization of LdSET29 in *Leishmania* promastigotes. A.** Analysis of whole cell extracts isolated from Ld1S transfectant promastigotes, by western blotting using anti-HA antibody (1:1000 dil; Cell Signaling Technology, USA; Catalog no: C29F4). Tubulin served as loading control. **B.** Upper panels: Analysis of LdSET29-HA localization at different cell cycle stages using immunofluorescence microscopy with anti-HA antibody (1:100 dilution). N: nucleus. K: kinetoplast. Cell cycle stage was determined in individual cells using kinetoplast morphology and segregation pattern as marker. G1/ early S phase: one roundish/short kinetoplast, one nucleus (1N1K). Late S/ early G2M phase: one elongated kinetoplast, one nucleus (1N1K). G2M phase: Two nuclei, one kinetoplast (2N1K). Post-mitosis: Two nuclei, two kinetoplasts (2N2K). Lower panels: Anti-HA antibody control. Cells expressing SET29-FLAG were stained with anti-HA antibody to rule out non-specific staining by anti-HA antibody. Cell sizes indicated using magnification bar.

Figure S4

A. *set29*$^{-/+}$::hyg

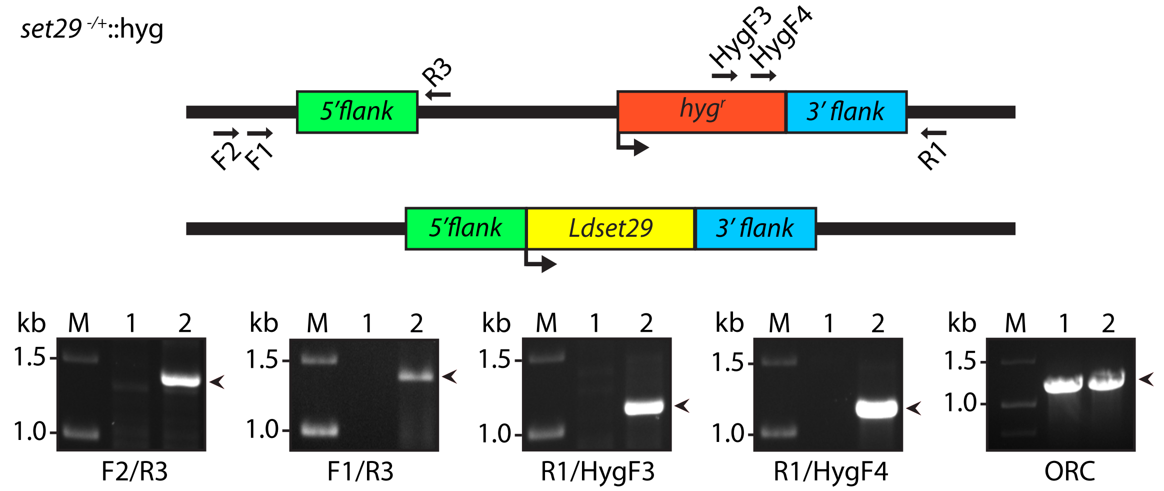

B. *set29*$^{-/+}$::neo

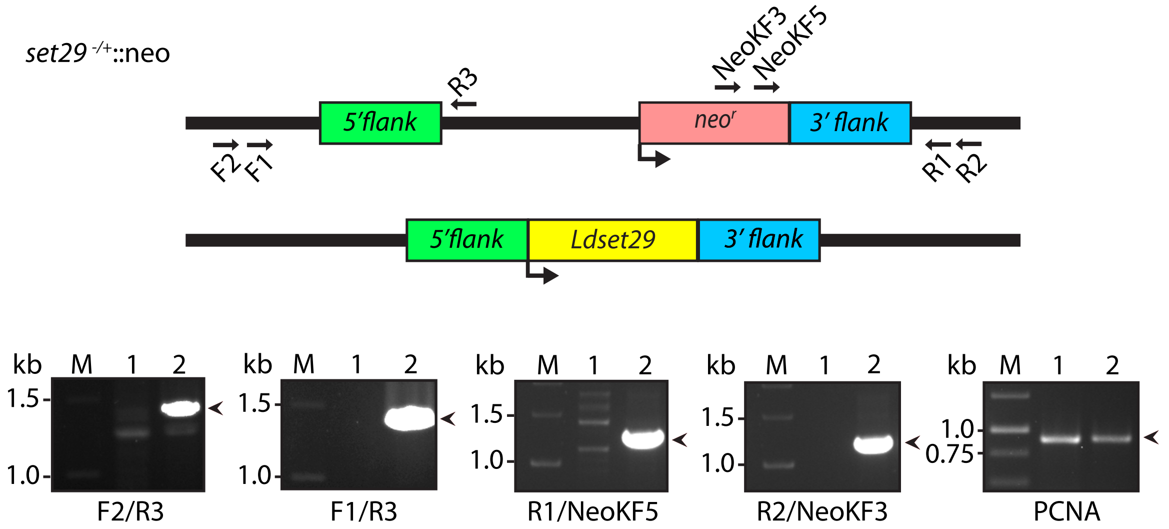

Figure S4: Creation of *set29*$^{-/+}$. **A.** Replacement of one *set29* genomic allele with hygromycin resistance cassette. **B.** Replacement of one set29 genomic allele with neomycin resistance cassette. PCRs were carried out across the deletion junctions to verify the authenticity of recombination at both ends. Labelled arrows on line diagrams indicate the primers used for PCRs. F1, F2, HygF3, HygF4, NeoKF3, NeoKF5: forward primers. R1, R2, R3: reverse primers. ORC and PCNA PCRs served as input template DNA control. Lanes 1: Ld1S genomic DNA template. Lanes 2: *set29*$^{-/+}$ genomic DNA template. M: DNA ladder.

Figure S5

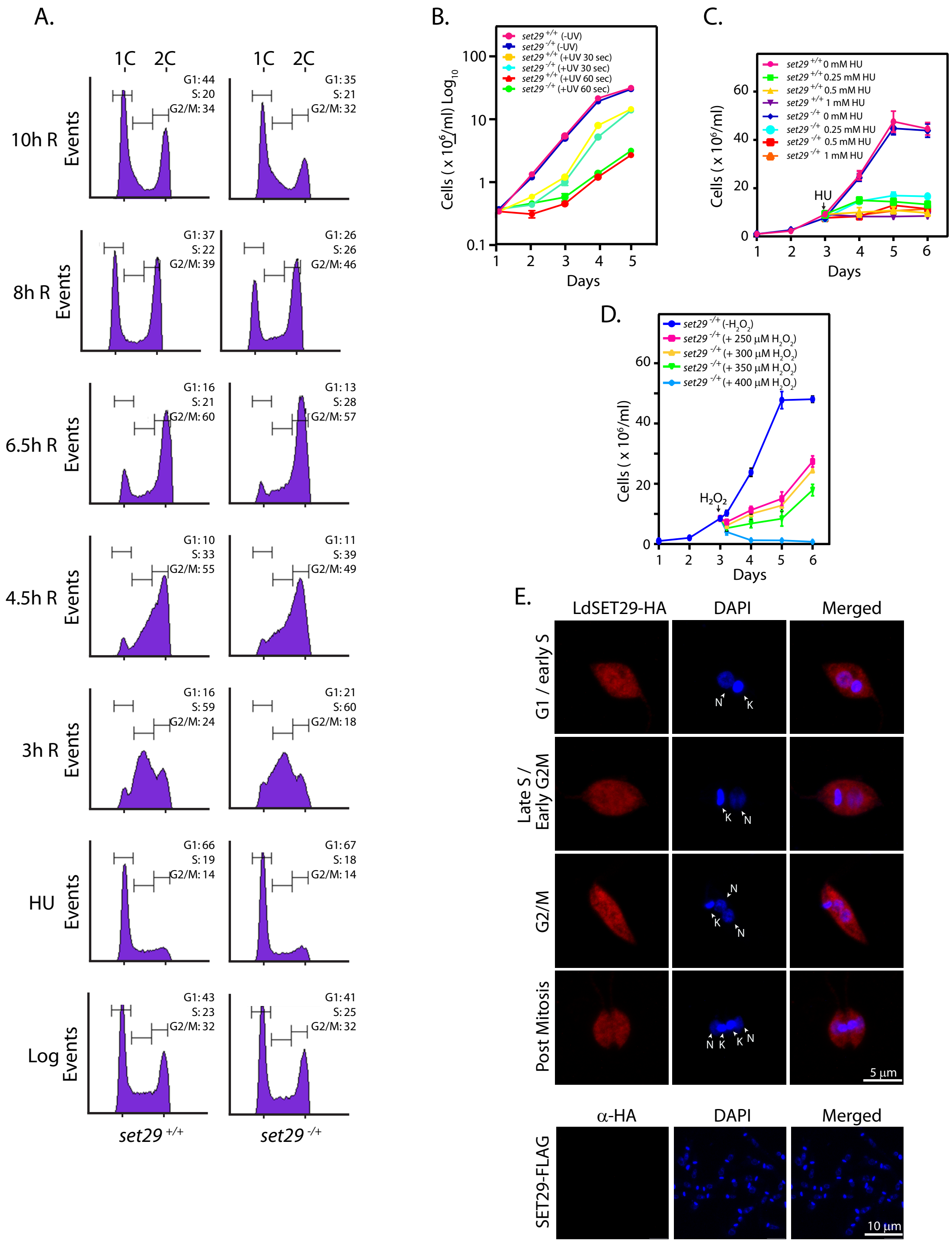

Figure S5: A. Analysis of cell cycle progression of HU-synchronized *set29*$^{-/+}$ parasites by flow cytometry. Sampling times are indicated on the left of the histograms (10h R indicates 10 hours after release from the HU block). At each sampling 25000-30000 events were analysed. G1, S and G2/M phases are represented by M1, M2 and M3 gating. The experiment was carried out three times, with similar results, and results of one experiment are shown. *set29*$^{+/+}$ cells: Ld1S::hyg cells. *set29*$^{-/+}$ cells: *set29*-heterozygous knockout cells with one allele replaced by hygromycin resistance cassette. **B.** Effect of UV irradiation (30 sec or 60 sec irradiations) on growth of *set29*$^{-/+}$ parasites in comparison with *set29*$^{+/+}$ cells. The experiment was performed thrice. Values shown are mean values of the three experiments, with error bars depicting standard deviation. **C.** Effect of HU-induced chronic stress on growth of *set29*$^{-/+}$ parasites in comparison with *set29*$^{+/+}$ cells. The experiment was performed thrice. Values shown are mean values of the three experiments, with error bars depicting standard deviation. D. Effect of exposure to higher concentrations of H_2O_2 on growth of *set29*$^{-/+}$ promastigotes. Cultures of *set29*$^{-/+}$ parasites were initiated from stationary phase promastigotes, with H_2O_2 being added on Day 3 (48 hours after initiation). The culture was divided into five parts at the time of addition of H_2O_2, with one part being continued as the untreated cells and the other parts receiving 250 µM to 400 µM H_2O_2 for 5h. The experiment was performed three times. Values shown are mean values of the three experiments, with error bars depicting standard deviation. **E.** Upper panels: Analysis of subcellular localization of LdSET29-HA after exposure to H_2O_2. Cultures were initiated at 1x10^6 cells/ml, and exposed to 100µM H_2O_2 for 5 hours on Day 3, before determining subcellular localization using immunofluorescence analysis. Kinetoplast morphology and segregation pattern served as cell cycle stage marker. Lower panels: Anti-HA antibody control. Cells expressing SET29-FLAG were stained with anti-HA antibody to rule out non-specific staining by anti-HA antibody. Cell sizes indicated using magnification bar**.**

Figure S6

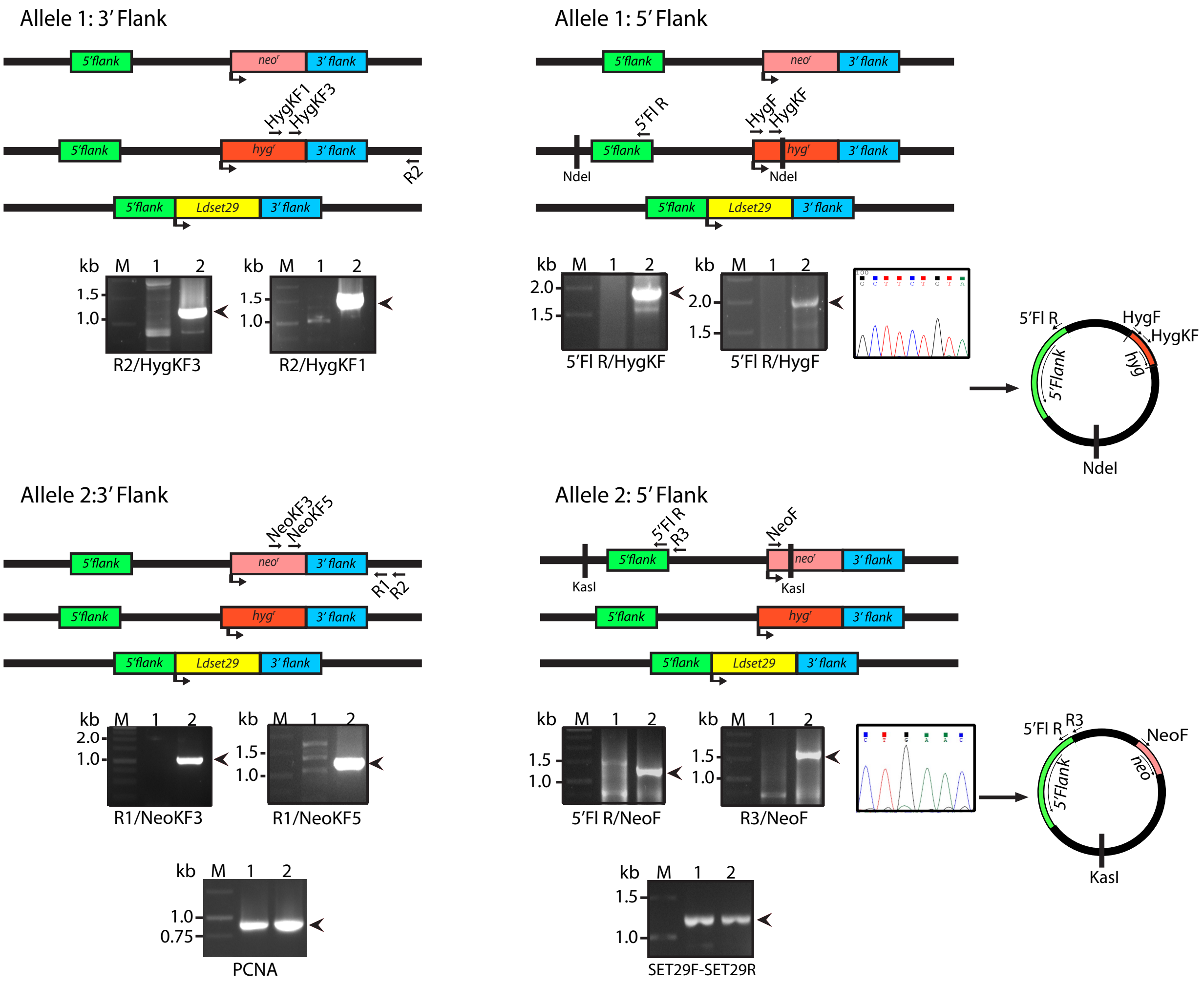

Figure S6: Creation of *set29*$^{-/-/+}$. PCRs were carried out across the deletion junctions to verify the authenticity of recombinations at the 3′ ends of both allelic replacements. The authenticity of recombinations at the 5′ ends of both allelic replacements were checked by inverse PCRs. To check integration of *hyg*^{r} cassette, genomic DNA was digested with NdeI enzyme (positions marked on the line diagram) and self-ligated. To check integration of *neo*^{r} cassette, genomic DNA was digested with KasI enzyme (positions marked on the line diagram) and self-ligated.

Labelled arrows on line diagrams indicate the primers used for PCRs. HygF, HygKF, HygKF1, HygKF3, NeoF, NeoKF3, NeoKF5: forward primers. R1, R2, R3, 5′Fl-R: reverse primers. SET29F-SET29R: set29 end primers. PCNA PCR served as input template DNA control. Lanes 1: Ld1S genomic DNA template. Lanes 2: *set29*$^{-/-/+}$ genomic DNA template. M: DNA ladder.

Figure S7

A. Step I: Construction of pLEXSY/YFPn

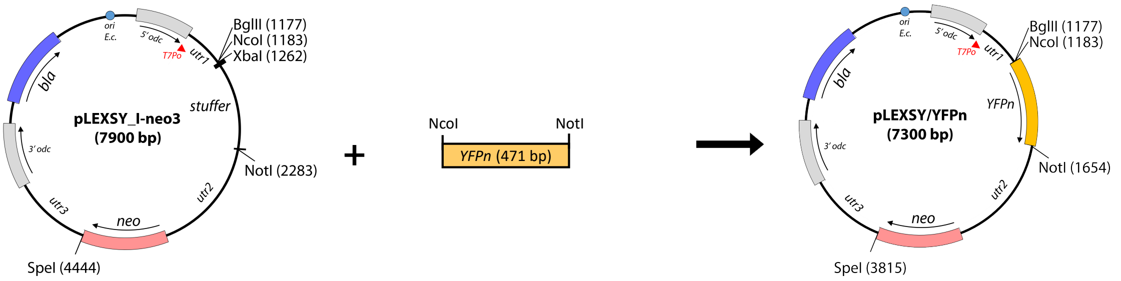

B. Step II: Construction of pXG/YFPc

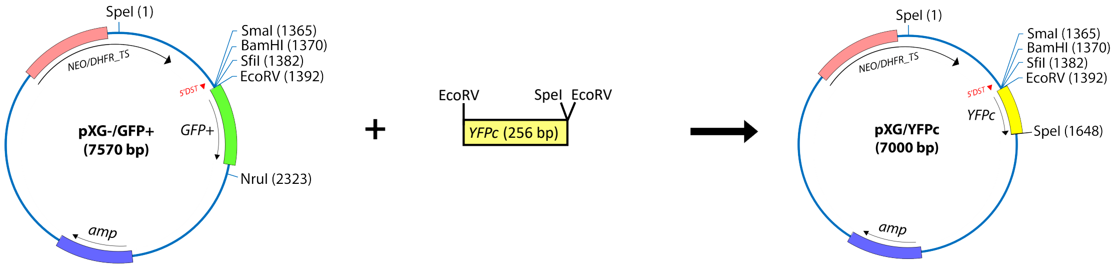

C. Step III: Construction of pLeish/YFPn/YFPc

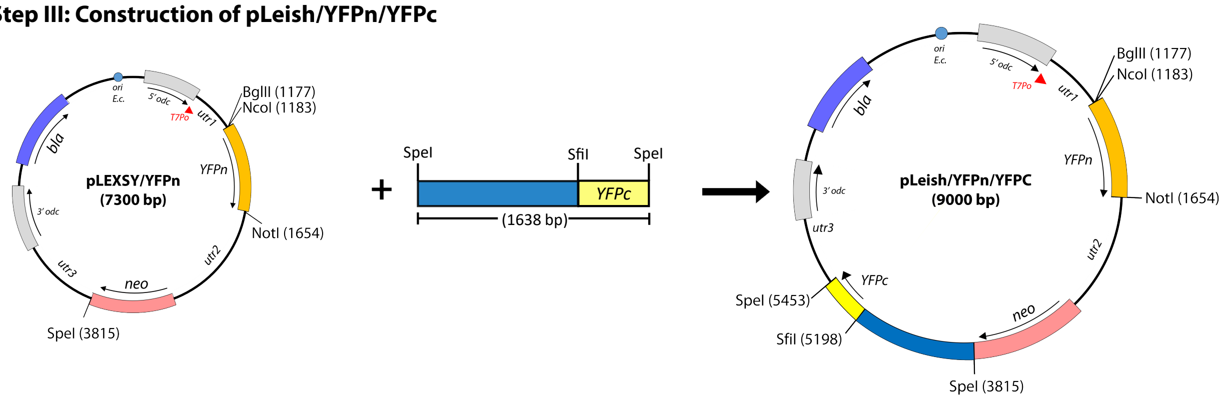

Figure S7: A-C: Schematic outline of construction of *Leishmania* BiFC vector. Maps of pLEXSY-derived vectors have been adapted from the map of pLEXSY_I-neo3 (www.jenabioscience.com). Map of pXG-derived vector has been adapted from the map of pXG-/GFP+ [53].

Figure S8

A.

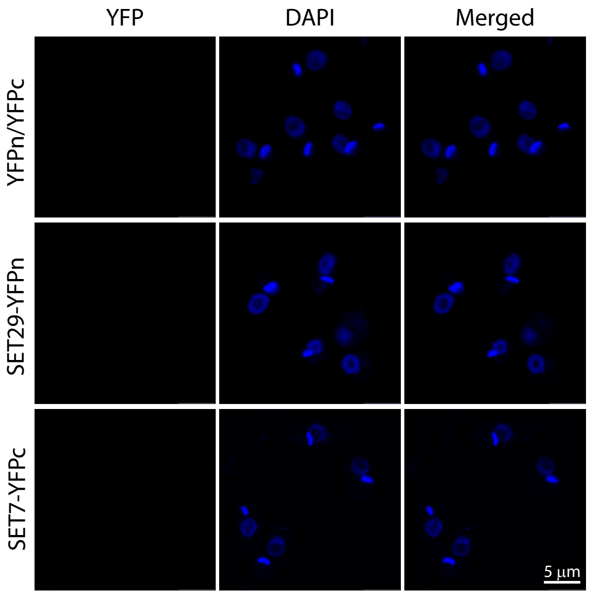

B.

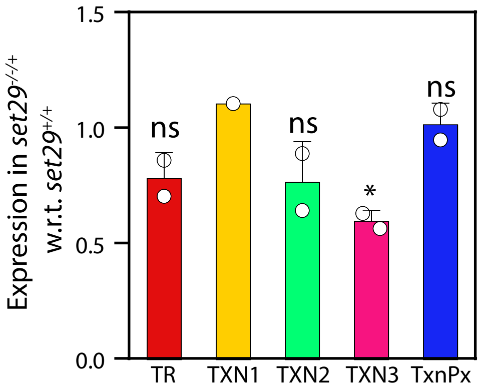

Figure S8: A. BiFC of control lines. YFPn/YFPc: Transfectants harboring pLeish/YFPn/YFPc. SET29-YFPn: Transfectants harboring the pLEXSY-YFPn vector with SET29 cloned in fusion with YFPn. SET7-YFPc: Transfectants harboring the vector with SET7 cloned in fusion with YFPc. Magnification bar: 5 µm **B.** Real time PCR analysis of genes of the trypanothione peroxidase pathway in *set29*$^{-/+}$ cells compared to *set29*$^{+/+}$ cells. TR: trypanothione reductase. TXN1: tryparedoxin 1 TXN2: tryparedoxin 2. TXN3: tryparedoxin 3. TxnPx: tryparedoxin peroxidase. Relative expression was determined using the using the $2^{-\Delta\Delta Ct}$ method, as earlier [21]. Average of two experiments are plotted, with open circles representing values of individual experiments. Statistical significance was determined using the two-tailed unpaired student's *t-test*. *: p value <0.05. ns: not significant.

Figure S9

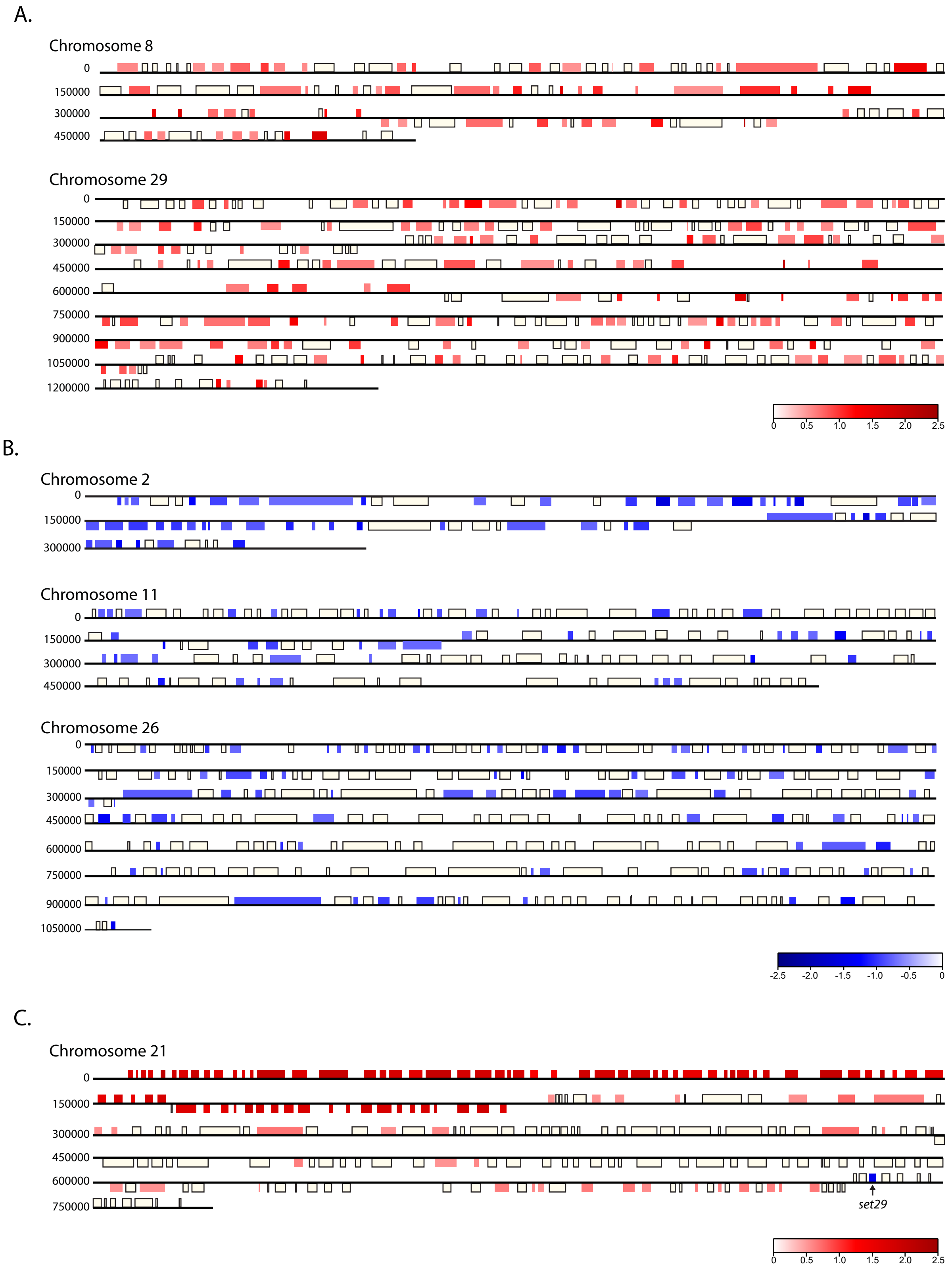

Figure S9: Chromosome-wide distribution of DEGs. A. Schematic representation of genes on chromosomes 8 and 29, carrying large numbers of upregulated genes in *set7* mutants. Color key indicates $\log_2$ fold changes **B.** Schematic representation of genes on chromosomes 2, 11 and 26, carrying large numbers of downregulated genes in *set7* mutants. Color key indicates $\log_2$ fold changes **C.** Schematic representation of genes on chromosome 21, carrying large numbers of upregulated genes in *set29* mutants. Color key indicates $\log_2$ fold changes. Chromosome maps have been adapted from genomic maps of *Leishmania donovani* BPK282A1 available on the TriTrypDB (www.tritrypdb.org).

Figure S10

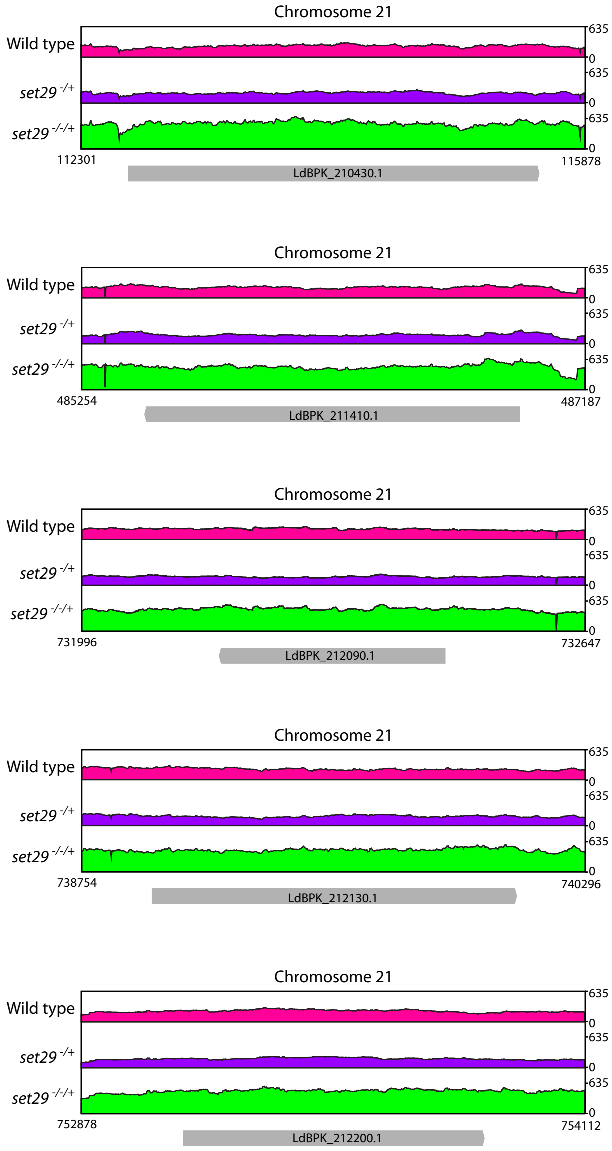

Figure S10: Sequencing tracks depicting various genes on chromosome 21 in wild type and *set29* mutant parasites. X axes: genome window defined by the indicated positions. Y axes: read depth
