# Supplementary material for: The SET29 and SET7 proteins of Leishmania donovani exercise non-redundant convergent as well as collaborative functions in moderating the parasite’s response to oxidative stress
Source: J Biol Chem. 2025 Jan 20;301(3):108208. doi: 10.1016/j.jbc.2025.108208 (PMC11871502; doi:10.1016/j.jbc.2025.108208)
Supplement: Supporting Information Tables S1-S3 [file mmc2.pdf]

The SET29 and SET7 proteins of *Leishmania donovani* exercise non-redundant convergent as well as collaborative functions in moderating the parasite's response to oxidative stress

Varshni Sharma[1#], Jyoti Pal[1#], Vishal Dashora[1], Somdeb Chattopadhyay[2], Yogita Kapoor[3,4], Biplab Singha[2,3,5], G. Aneeshkumar Arimbasseri[2] and Swati Saha[1*]

**[1]Department of Microbiology,
University of Delhi South Campus,
New Delhi, India**

**[2]National Institute of Immunology,
New Delhi, India.**

**[3]Centre for Cellular and Molecular Biology,
Hyderabad, India.**

**[4] Academy of Scientific and Innovative Research (AcSIR),
Ghaziabad, India.**

**[5] Current affiliation:
Department of Medicine and Biological Sciences,
Cedars-Sinai Samuel Oschin Comprehensive Cancer Institute,
Los Angeles, USA**

# *These two authors contributed equally to the work*

To whom correspondence may be addressed

Running title: Role of *Leishmania donovani* SET29 and SET7 proteins

Keywords: *Leishmania donovani*, trypanosome, SET domain, SET proteins, SET29, SET7, oxidative stress, protozoan parasite, BiFC in *Leishmania*

Table S1: DEGs in *set7* mutant parasites relative to wild type parasites (both untreated)						
**S.No**	**Gene ontology term**	**Gene ID**	**Product Description**	**Log_2 Fold change**	**Fold change**	**padj**
1	**Autophagy**	LdBPK_080410.1	hypothetical protein, conserved	0.69063926	1.613998525	3.4865E-24
2		LdBPK_090180.1	ATG8/AUT7/APG8/PAZ2, putative	1.313440669	2.48533559	1.5572E-32
3		LdBPK_090200.1	microtubule associated protein-like protein	1.223780561	2.335579513	1.39511E-07
4		LdBPK_190840.1	ATG8/AUT7/APG8/PAZ2, putative	2.18971423	4.562151099	2.1493E-100
5		LdBPK_191660.1	ATG8/AUT7/APG8/PAZ2, putative	0.643500285	1.562114587	5.50558E-07
6		LdBPK_291320.1	COPII coat assembly protein sec16, putative	0.682702229	1.605143443	3.60801E-23
7		LdBPK_300270.1	AUT2/APG4/ATG4 cysteine peptidase, putative	0.581551803	1.496458015	0.000164079
8	**Response to oxidative stress**	LdBPK_151120.1	tryparedoxin peroxidase	1.101387508	2.145609467	3.32071E-22
9		LdBPK_260780.1	glutathione peroxidase-like protein, putative	0.735101338	1.664514384	2.62783E-20
10		LdBPK_282860.1	SelR domain containing protein, putative	0.966488957	1.954079221	2.45211E-15
11		LdBPK_340070.1	ascorbate peroxidase, putative	0.573669406	1.488304171	1.11741E-16
12		LdBPK_366080.1	Nitroreductase family, putative	0.652591718	1.571989649	7.70626E-08
13	**Phosphorylation**	LdBPK_041230.1	casein kinase I, putative	0.903108767	1.870091379	7.50E-10
14		LdBPK_070330.1	protein kinase, putative	1.155781334	2.228049576	5.52E-43
15		LdBPK_080540.1	protein kinase , putative	0.559274528	1.473528054	6.99E-16
16		LdBPK_080670.1	protein kinase, putative	1.391469409	2.623457483	1.27E-53
17		LdBPK_081250.1	protein kinase, putative	1.685894752	3.217398758	2.16E-132
18		LdBPK_141380.1	protein kinase, putative	0.595542008	1.511040164	4.69E-11
19		LdBPK_141510.1	protein kinase, putative	0.728588084	1.657016633	6.63E-10
20		LdBPK_170440.1	protein kinase, putative	0.772444341	1.70816145	2.02E-10
21		LdBPK_191290.1	hypothetical protein, unknown function	1.252833352	2.383089859	3.14E-40
22		LdBPK_191480.1	mitogen-activated protein kinase 4	0.618818603	1.535617177	3.42E-14
23		LdBPK_220770.1	protein kinase, putative	0.623308949	1.540404186	4.69E-06
24		LdBPK_241500.1	protein kinase, putative	0.697931668	1.622177477	3.22E-13
25		LdBPK_271680.1	casein kinase I-like protein	1.097138281	2.139299215	6.89E-09
26		LdBPK_281880.1	protein kinase, putative	0.57134747	1.485910756	3.70E-13
27		LdBPK_290250.1	RIO1 family, putative	0.708439248	1.63403541	5.94E-18
28		LdBPK_290380.1	protein kinase-like protein	0.750121653	1.681934652	1.71E-18
29		LdBPK_291420.1	serine/threonine-protein kinase, putative	0.592576717	1.507937587	5.23E-16
30		LdBPK_292140.1	5'-AMP-activated protein kinase catalytic subunit alpha, putative	0.688239164	1.611315678	2.68E-11
31		LdBPK_292260.1	cdc2-related kinase 10, putative	0.685875458	1.608677865	1.88E-05
32		LdBPK_292430.1	mitogen-activated protein kinase kinase 1	0.868539182	1.825813215	7.90E-34
33		LdBPK_292600.1	protein kinase, putative	1.081625049	2.116418668	1.68E-25
34		LdBPK_292680.1	serine/threonine-protein kinase, putative	0.691382582	1.614830322	7.08E-27
35		LdBPK_292780.1	serine/threonine-protein kinase Nek, putative	0.868451558	1.825702325	1.01E-23
36		LdBPK_321900.1	protein kinase, putative	0.795860386	1.736112438	5.17E-06
37		LdBPK_332100.1	serine/threonine-protein kinase a, putative	1.046513575	2.065532234	3.22E-13
38		LdBPK_350480.1	Protein kinase domain containing protein, putative	0.696478386	1.620544217	4.93E-31
39		LdBPK_355330.1	protein kinase, putative	0.549502752	1.463581162	1.14E-05

40		LdBPK_360920.1	mitogen-activated protein kinase kinase 5	0.625590048	1.542841703	1.90E-06
41		LdBPK_361320.1	fructose-1,6-bisphosphate aldolase	0.703793565	1.628782047	1.02E-66
42		LdBPK_361590.1	protein kinase, putative	0.587885326	1.503042002	8.39E-07
43		LdBPK_362420.1	protein kinase, putative	0.682042442	1.604409531	0.033796307
44		LdBPK_364770.1	related to elongation factor-2 kinase efk-1b isoform-like protein	0.789055225	1.727942518	2.52E-10
45	**Lipid metabolic process**	LdBPK_080160.1	GPI-GlcNAc transferase complex, PIG-H component, putative	0.746789106	1.678053957	7.00E-19
46		LdBPK_080210.1	Inositol phosphosphingolipids phospholipase C	0.837384607	1.786807985	3.22E-20
47		LdBPK_130200.1	Lipase (class 3), putative	0.948854327	1.930339128	4.07E-23
48		LdBPK_140520.1	stearic acid desaturase, putative	1.07237112	2.102886696	2.60E-09
49		LdBPK_140650.1	hypothetical protein, unknown function	0.875311169	1.834403699	6.16E-07
50		LdBPK_140750.1	fatty acid elongase, putative	0.820431696	1.765934332	9.02E-14
51		LdBPK_141420.1	delta-6 fatty acid desaturase, putative	0.663304453	1.583705907	2.67E-22
52		LdBPK_191370.1	membrane-bound O-acyltransferase, putative	1.015609307	2.021756581	1.17E-49
53		LdBPK_210500.1	ubiquitin-conjugating enzyme-like protein	0.707465523	1.632932915	2.43E-20
54		LdBPK_240840.1	inositol polyphosphate phosphatase, putative	1.697523104	3.243436297	7.12E-122
55		LdBPK_291350.1	Wnt-binding factor required for Wnt secretion/Lipase (class 3), putative	0.866887414	1.823724005	2.98E-34
56		LdBPK_291370.1	lipase domain protein, putative	0.558208895	1.472440049	2.68E-20
57		LdBPK_292150.1	N-acetylglucosamyl transferase component GPI1, putative	0.607317735	1.523424217	1.16E-13
58		LdBPK_301840.1	phosphatidylinositol kinase, putative	1.014429732	2.020104229	8.37E-17
59		LdBPK_312540.1	lipase, putative	1.144336552	2.210444562	1.01E-23
60		LdBPK_351480.1	choline/ethanolamine kinase, putative	0.540282407	1.454257159	2.44E-10
61		LdBPK_354660.1	phosphatidylserine decarboxylase, putative	0.729822846	1.658435434	3.68E-10
62		LdBPK_355030.1	sphingomyelin/ceramide phosphorylethanolamine synthase, bifunctional	0.608542132	1.524717677	1.38E-09
63		LdBPK_362670.1	C-4 sterol methyl oxidase, putative	0.748354291	1.679875472	1.30E-10
64		LdBPK_362710.1	related to multifunctional cyclin-dependent kinase pho85-like protein	1.045294442	2.063787518	3.03E-41
65		LdBPK_367290.1	delta8 fatty acid desaturase-like protein	0.70362731	1.628594359	3.31E-18
66	**Response to stress**	LdBPK_071090.1	Domain of unknown function (DUF3437), putative	0.666066906	1.586741271	3.51108E-31
67		LdBPK_100030.1	Dos2-interacting transcription regulator of RNA-Pol-II, putative	0.561488188	1.47579076	2.3058E-15
68		LdBPK_211100.1	mis-match repair protein, putative	1.010825739	2.01506411	8.25643E-24
69		LdBPK_260780.1	glutathione peroxidase-like protein, putative	0.735101338	1.664514384	2.62783E-20
70		LdBPK_272300.1	Vesicle-associated membrane protein 7	0.593350698	1.508746787	0.000181314
71		LdBPK_282860.1	SelR domain containing protein, putative	0.966488957	1.954079221	2.45211E-15
72		LdBPK_291140.1	KU70 protein, putative	0.644827779	1.563552627	2.54684E-23
73		LdBPK_291700.1	XPC-binding domain containing protein, putative	0.558417211	1.472652675	4.29287E-16
74		LdBPK_291840.1	MutS-like protein	0.644596558	1.563302056	6.25838E-08
75		LdBPK_310030.1	Aquaglyceroporin 1	0.728516079	1.656933933	9.05522E-09

76		LdBPK_340070.1	ascorbate peroxidase, putative	0.573669406	1.488304171	1.11741E-16
77		LdBPK_352250.1	kinetoplastid membrane protein-11	1.234077552	2.352308945	8.94696E-11
78		LdBPK_354950.1	meiotic recombination protein DMC1, putative	0.606901754	1.522985023	0.000973328
79		LdBPK_361320.1	fructose-1,6-bisphosphate aldolase	0.703793565	1.628782047	1.0182E-66
80		LdBPK_366080.1	Nitroreductase family, putative	0.652591718	1.571989649	7.70626E-08
81		LdBPK_010590.1	tricarboxylate carrier, putative	-0.795358191	0.576200097	4.31E-22
82	**ion transport**	LdBPK_020420.1	voltage-dependent anion-selective channel, putative	-1.015069623	0.494804449	1.29E-43
83		LdBPK_020430.1	Mitochondrial outer membrane protein porin, putative	-1.351612959	0.391853704	1.05E-111
84		LdBPK_051140.1	V-type proton ATPase subunit D, putative	-0.748194723	0.595348064	1.43E-17
85		LdBPK_180560.1	vacuolar ATP synthase subunit c, putative	-0.806995892	0.571570795	1.26E-22
86		LdBPK_190200.1	ADP,ATP carrier protein 1, mitochondrial precursor, putative	-0.787222795	0.579458483	1.31E-67
87		LdBPK_210820.1	ATPase subunit 9, putative	-0.729842942	0.602969552	4.53E-17
88		LdBPK_212140.1	ATP synthase F1 subunit gamma protein, putative	-1.056834984	0.480685442	9.85E-76
89		LdBPK_282050.1	Zinc transporter 3, putative	-0.788290041	0.579029983	1.87E-10
90		LdBPK_303660.1	ATP synthase, epsilon chain, putative	-0.756900488	0.591766328	4.13E-34
91		LdBPK_313190.1	iron/zinc transporter protein-like protein	-0.783415523	0.580989694	6.12E-42
92		LdBPK_343460.1	vacuolar ATP synthase catalytic subunit A, putative	-0.692845238	0.618632598	1.31E-27
93		LdBPK_060010.1	histone H4	1.193793291	2.287534166	1.97E-178
94	**Others**	LdBPK_091410.1	histone H2B	1.121934376	2.176385884	7.94E-138
95		LdBPK_101050.1	histone H3	1.245385987	2.37081976	1.62E-35
96		LdBPK_150010.1	histone H4	0.805073895	1.747235283	1.82E-72
97		LdBPK_211160.1	histone H2A	0.736992315	1.666697536	4.55E-71
98		LdBPK_271070.1	histone H1, putative	0.878388975	1.838321342	1.13E-72
99		LdBPK_291250.1	tryparedoxin 1, putative	0.845110548	1.796402391	9.25E-51
100		LdBPK_060800.1	Double RNA binding domain protein 12	0.652441769	1.57182627	1.44E-09
101		LdBPK_291440.1	RNA-binding protein, putative	0.948630278	1.930039372	0.014396061
102		LdBPK_291500.1	RNA binding protein, putative	0.979904071	1.972334258	0.000520403
103		LdBPK_291510.1	RNA binding protein, putative	0.654316233	1.573869839	3.86E-21
104		LdBPK_302200.1	RNA-binding protein, putative	0.753470881	1.685843811	6.42E-07
105		LdBPK_331560.1	RNA-binding protein, putative	0.993897384	1.991557846	0.006853984

Table S1: DEGs in *set7* mutants relative to wild type parasites. The listed differentially regulated genes are grouped based on their GO terms.

Table S2: DEGs in *set29* mutant parasites treated with hydrogen peroxide relative to untreated set29 mutant parasites						
**S.No**	**Gene ontology term**	**Gene ID**	**Product Description**	**Log_2 Fold change**	**Fold change**	**padj**
1	**Ribosome Biogenesis**	LdBPK_070140.1	pre-rrna-processing protein tsr1 homolog	0.578394826	1.493186972	2.90E-10
2		LdBPK_110470.1	pumilio-repeat, RNA-binding protein, putative	0.621755103	1.538745996	1.36E-13
3		LdBPK_151530.1	ribosomal protein S6, putative	0.803607082	1.745459741	1.20E-36
4		LdBPK_171350.1	RNA cytidine acetyltransferase	0.572310276	1.486902733	1.01E-15
5		LdBPK_171480.1	NUC130/3NT domain/SDA1, putative	0.551541716	1.465651107	2.20E-08
6		LdBPK_191200.1	Fcf1, putative	0.554969268	1.469137338	0.001384
7		LdBPK_230060.1	cyclophilin, putative	0.701460237	1.626149882	9.03E-16
8		LdBPK_271900.1	FtsJ cell division protein, putative	0.581736331	1.496649432	2.19E-23
9		LdBPK_301880.1	Adenylate kinase, nuclear	0.660026485	1.580111632	5.52E-06
10		LdBPK_331430.1	hypothetical protein, conserved	0.701582728	1.626287955	5.39E-11
11		LdBPK_344120.1	nucleolar protein family a, putative (fragment)	0.597796931	1.513403756	2.42E-17
12		LdBPK_360950.1	Eukaryotic translation initiation factor 6 (eIF-6), putative	0.842776835	1.793498868	4.91E-16
13		LdBPK_363220.1	fibrillarin	0.724806226	1.652678644	3.33E-25
14		LdBPK_364880.1	60S acidic ribosomal protein, putative	0.724159288	1.651937711	9.22E-14
15		LdBPK_366680.1	40S ribosomal protein S8, putative	0.700698371	1.625291364	1.12E-11

Table S2: DEGs in H_2O_2-treated *set29* mutants relative to untreated *set29* mutants. The listed differentially regulated genes are grouped based on their GO terms

Table S3: Ratio 'r' of read depth of each gene to median chromosomal read depth					
**Accession number**	**Annotation**	**Wild type**	***set7*** $^{-/-}$	***set29*** $^{-/+}$	***set29*** $^{-/-/+}$
**LdBPK_212120.1**	SET 29	0.88	0.88	0.62	0.21
**LdBPK_210430.1**	Rtr1/RPAP2 family, putative	1.06	1.04	1.09	1.09
**LdBPK_211410.1**	surface antigen-like protein	0.99	0.90	0.93	0.97
**LdBPK_212090.1**	60S ribosomal protein L32	1	1.00	0.89	0.96
**LdBPK_212130.1**	centromere/microtubule binding protein cbf5, putative	0.97	0.86	0.98	0.91
**LdBPK_212200.1**	proteasome subunit alpha type-5, putative	1.08	1.04	1.02	0.99
**LdBPK_360230.1**	SET 7	1.05	0	0.99	1.08
**LdBPK_360220.1**	Mitochondrial inner membrane signal peptidase, putative	1.05	1.04	0.94	0.93
**LdBPK_360240.1**	Dolicholphosphate-mannose synthase	1	1.04	0.97	1.06
**LdBPK_360260.1**	Afadin- and alpha -actinin-Binding, putative	1.17	1.00	1.02	1.03
**LdBPK_365890.1**	Cyclin 12, L-type	0.99	1.09	1.03	1.05

Table S3: Ratio 'r' of read depth of particular genes to median chromosomal read depth.
Genes lying near *set29* (LdBPK_212090.1, LdBPK_212130.1, LdBPK_212200.1), as well as those lying distant from *set29* (LdBPK_210430.1, LdBPK_211410.1) on chromosome 21 were selected for the analysis. Similarly, genes lying near *set7* (LdBPK_360220.1, LdBPK_360240.1, LdBPK_360260.1), as well as those lying distant from *set7* (LdBPK_365890.1) on chromosome 36 were selected for the analysis.
